# Beyond local control: radiotherapy–immunotherapy combinations from *in situ* vaccination to systemic immune remodeling

**DOI:** 10.3389/fgene.2026.1927654

**Published:** 2026-09-17

**Authors:** Yulong Wen, Nannan Cheng, Sheng Zhou, Haixia Zhu, Qin Ge

**Affiliations:** 1 Department of Radiotherapy, Nantong University School of Medicine, Nantong, China; 2 Department of Radiotherapy, Nantong Tumor Hospital, Nantong, China

**Keywords:** abscopal effect, immune checkpoint, immunogenic cell death, immunotherapy, radiotherapy, tumor immune microenvironment

## Abstract

**Background:**

Radiotherapy (RT) is evolving from local cytotoxicity to systemic immunomodulation. Acting as an *in situ* vaccine, RT initiates antitumor immunity via immunogenic cell death and cGAS-STING activation. However, RT concurrently triggers counter-regulatory immunosuppression—upregulating PD-L1/TGF-β, expanding Tregs/MDSCs, and inducing T-cell exhaustion—limiting durable systemic control.

**Methods:**

We integrate preclinical mechanisms and key clinical evidence for radioimmunotherapy combinations. We analyze how radiation dose, fractionation, and delivery techniques remodel the tumor immune microenvironment and outline matching therapeutic strategies

**Key findings:**

Optimal combination requires balancing local tumor control with systemic immune activation while preserving lymphoid reserves. Consolidation durvalumab following concurrent chemoradiotherapy has been shown to improve survival in patients with unresectable stage III non-small cell lung cancer, and SBRT combined with PD-(L)1 blockade has demonstrated benefits in oligometastatic settings. Conversely, radioimmunotherapy combinations have yielded disappointing outcomes in extensive-stage small cell lung cancer and head and neck cancers. Low-dose RT and novel spatial-fractionation strategies offer emerging solutions to reverse immune exclusion.

**Conclusion:**

Efficacy hinges on converting RT-induced immunoadaptation into productive antitumor immunity. Future progress demands biomarker-driven patient selection (e.g., tumor mutational burden, chromosomal instability) and precise orchestration of multi-modality timing. Transitioning toward “immunologically guided precision radiotherapy” will transform RT from a static local therapy into a dynamic component of systemic cancer care.

## 1 Introduction

Radiotherapy (RT) is undergoing a paradigm shift: evolving from a local cytotoxic agent into a systemic immune modulator. As an “*in situ* vaccine,” RT triggers antitumor immunity by inducing immunogenic cell death and activating cGAS-STING pathways, thereby priming systemic T cell responses and abscopal effects against distant metastases (Delaney et al., 2005; Formenti and Demaria, 2012; Jaffray, 2012; Thariat et al., 2013; Herrera et al., 2017).

However, RT is a double-edged sword. It also upregulates immunosuppressive signals (PD-L1, TGF-β), expands regulatory T cells, and recruits myeloid-derived suppressor cells, creating an immunosuppressive microenvironment (Schaue et al., 2012; Gros et al., 2014; Vanpouille-Box et al., 2015; Kim K. J. et al., 2017; Muroyama et al., 2017; Gunderson et al., 2020; Sia et al., 2021; Yoon et al., 2022; Wang L. et al., 2024). This duality constitutes the central bottleneck in transforming RT into a reliably potent immunotherapeutic modality. The core challenge has shifted from demonstrating RT’s immunomodulatory potential to precisely calibrating its bidirectional effects.

This narrative review establishes an integrated framework to tackle this core challenge. We first dissect the opposing mechanisms of RT-triggered immune activation and immunosuppression. Next, we elaborate on combinatorial regimens pairing RT with immune checkpoint inhibitors, adoptive cell therapies, immune agonists, and other agents to harness radiation’s immunomodulatory capacity for long-term antitumor immune responses (Demaria et al., 2004; Dewan et al., 2009; Park et al., 2015; Dovedi et al., 2017; Gong et al., 2017; Morisada et al., 2018; Ngwa et al., 2018; Donlon et al., 2021). Lastly, we synthesize translational data and clinical hurdles across various malignancies, laying out future directions for immunology-guided precision radiotherapy.

## 2 Radiotherapy: a paradigm shift from local cytotoxic agent to systemic immunomodulator

The design of conventional radiotherapy has historically centered on dose escalation constrained by normal-tissue tolerance, target coverage and therapeutic ratio with the therapeutic goal restricted to directly eradicating tumor cells via radiation-induced DNA damage to achieve local control, functioning merely as a local cytotoxic modality (Thariat et al., 2013). With the elucidation of key biological mechanisms—including immunogenic cell death (ICD), the cGAS-STING innate immune pathway, and the release of damage-associated molecular patterns (DAMPs) (Formenti and Demaria, 2012; Deng et al., 2014b)—radiotherapy is now recognized to possess potent *in situ* vaccination capacity, capable of systemically remodeling the antitumor immune microenvironment. Driven by these mechanistic advances, the conceptual framework of radiotherapy has undergone a fundamental transformation: clinical practice no longer focuses solely on local tumor ablation but instead emphasizes striking an optimal balance among local tumor control, systemic immune activation, and lymphocyte preservation. Supported by decades of high-level clinical evidence for stereotactic body radiation therapy (SBRT) and early investigations into emerging strategies such as low-dose radiotherapy, radiotherapy has formally evolved from a simple local tumoricidal tool into a systemic modulator capable of regulating antitumor immune responses, representing a dual paradigm shift in both therapeutic role and clinical application (Herrera et al., 2017).

### 2.1 Mechanisms of immune activation

RT initiates and enhances antitumor immunity by converting local tissue damage into systemic immune signals (Figure 1). The core mechanisms encompass the induction of ICD, activation of the cGAS-STING pathway, release of key DAMPs, dose-dependent regulation of T-cell recruitment, and activation of natural killer (NK) cells.

**FIGURE 1:**
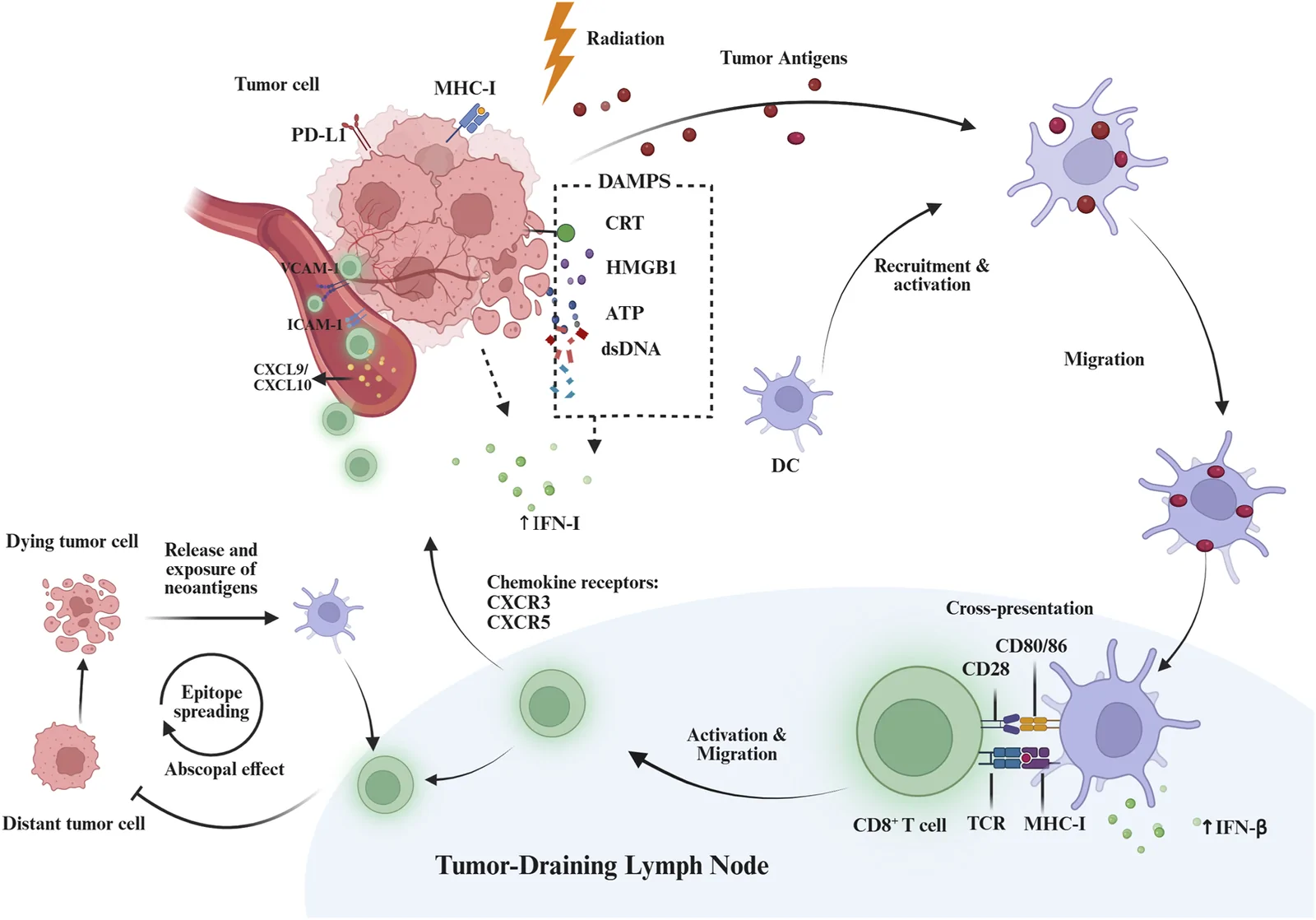
Radiotherapy as an *in situ* vaccine: from local injury to systemic antitumor immunity. Schematic illustration depicting the core process by which radiotherapy (RT) functions as an *in situ* vaccine to convert local damage signals into systemic antitumor immunity, based on the classical cancer-immunity cycle framework by Chi et al. (2023). The cascade follows a tripartite “release–prime–attack” paradigm: 1) Immunogenic cell death and antigen release – RT induces tumor cell death, releasing tumor (neo)antigens and damage-associated molecular patterns (DAMPs), with calreticulin (CRT) serving as an “eat me” signal, and HMGB1/ATP acting as “alarm”/“find me” signals; 2) Antigen presentation and T cell priming – Dendritic cells (DCs) capture these signals, become activated, migrate to tumor-draining lymph nodes, and cross-present antigens to prime tumor-specific CD4^+^ and CD8^+^ T cells; 3) Effector expansion and systemic attack – Activated cytotoxic T lymphocytes (CTLs) home to the primary tumor for local killing while circulating to eliminate distant metastases, thereby eliciting the “abscopal effect,” which can be amplified by antigen spread. The schematic illustrates the mechanistic basis by which RT converts immunologically “cold” tumors into “hot” ones and synergizes with immune checkpoint inhibitors.

#### 2.1.1 Induction of immunogenic cell death

ICD serves as the cornerstone of radiotherapy-induced antitumor immunity. Dying tumor cells release tumor-associated antigens (TAAs), double-stranded DNA (dsDNA), and DAMPs, collectively forming an *in situ* vaccine. These signals activate antigen-presenting cells (APCs), driving type I interferon (IFN-I) secretion. This promotes dendritic cell (DC) maturation and migration to tumor-draining lymph nodes (tdLNs), where they cross-present antigens via major histocompatibility complex (MHC) molecules, thereby initiating tumor-specific CD8^+^ and CD4^+^ T-cell clones (Woo et al., 2014; Demaria et al., 2015; Weichselbaum et al., 2017; Diamond et al., 2018; Rodríguez-Ruiz et al., 2018; McLaughlin et al., 2020). These activated clones not only mediate local tumoricidal effects but also enter the circulation to eliminate minimal residual disease, manifesting the systemic abscopal effect (Formenti and Demaria, 2012).

Activated T cells home to the tumor along chemokine gradients, undergoing the classic “roll, adhere, and extravasate” process to infiltrate the tumor parenchyma. Cytotoxic CD8^+^ T cells recognize MHC-I-antigen peptide complexes and release perforin and granzyme to directly lyse target cells; concurrently, activated CD4^+^ T cells (primarily the Th1 subset) potentiate CD8^+^ T-cell function via cytokines such as IFN-γ (Kim and Chen, 2016; Nagarsheth et al., 2017; Salomon et al., 2020; Donlon et al., 2021; Lhuillier et al., 2021; Chi and Nguyen, 2023).

Furthermore, radiotherapy enhances immunogenicity through non-ICD pathways. Radiation-induced cellular stress activates the mammalian target of rapamycin (mTOR) pathway, promoting neoantigen generation and upregulating antigen-processing machinery and MHC-I expression, thereby enhancing tumor antigen presentation efficiency (Reits et al., 2006; Lussier et al., 2021).

#### 2.1.2 Activation of the cGAS-STING pathway

Radiation-induced DNA double-strand breaks lead to the release of dsDNA into the cytoplasm. Cytosolic dsDNA is recognized by cyclic GMP-AMP synthase (cGAS), which catalyzes the synthesis of the second messenger cyclic GMP-AMP (cGAMP). cGAMP subsequently binds to and activates stimulator of interferon genes (STING), triggering TANK-binding kinase 1 (TBK1)-mediated phosphorylation and nuclear translocation of the transcription factors IRF3 and NF-κB. This culminates in the transcription of type I interferons (primarily IFN-β) and various pro-inflammatory cytokines (Widau et al., 2014; Rodríguez-Ruiz et al., 2018; Vanpouille-Box et al., 2018; Huang and Zhou, 2020; McLaughlin et al., 2020). IFN-β acts as a key type I interferon, providing a maturation license for DCs, enhancing co-stimulatory molecule expression and cross-presentation capacity, thereby robustly priming antigen-specific CD8^+^ T-cell responses (Deng et al., 2014b; Woo et al., 2014). This pathway is subject to negative feedback regulation by the DNA exonuclease Trex1, indicating a complex kinetic relationship between radiation dose and immune activation (Vanpouille-Box et al., 2017; Chi and Nguyen, 2023).

#### 2.1.3 Orchestrated release and combined effects of key DAMPs

During ICD, DAMPs constitute a potent adjuvant signal cascade. Calreticulin (CRT) acts as an “eat-me” signal, guiding DCs to recognize and phagocytose apoptotic cells (Golden et al., 2014; Hernandez et al., 2016). Adenosine triphosphate (ATP) serves as a “find-me” signal, activating the P2RX7 receptor to drive IL-1β secretion and facilitate T-cell cross-priming (Ben-Sasson et al., 2009; Ghiringhelli et al., 2009; Ben-Sasson et al., 2013; Guo et al., 2023). High mobility group box 1 (HMGB1) functions as an “alarmin,” providing a strong DC maturation stimulus via TLR4 (Apetoh et al., 2007; Lai et al., 2019). These three signals act sequentially to collectively amplify the antitumor immune response.

#### 2.1.4 Dose-dependent regulation of T cell recruitment

RT exhibits dose-dependent regulation of T-cell infiltration. Moderate-to-high dose fractionated irradiation upregulates CXCL9/CXCL10 and ICAM-1/VCAM-1, optimizing the classic T-cell homing pathway (Lugade et al., 2005; Matsumura et al., 2008; Matsumura and Demaria, 2010; Lai et al., 2019; Zhao et al., 2021). Conversely, single high-dose irradiation impedes T-cell infiltration due to severe vascular damage (Klug et al., 2013; Demaria et al., 2015; Kozin, 2022). In contrast, low-dose radiotherapy remodels the microenvironment—via macrophage polarization, iNOS induction, and IFN-γ-mediated effects—to establish alternative T-cell recruitment routes, effectively reversing the “cold tumor” immunosuppressive state (Klug et al., 2013; Herrera et al., 2022b). These dose-specific effects provide a theoretical basis for individualized radiotherapy regimens.

#### 2.1.5 Activation of natural killer cells

RT modulates NK-cell function via two primary routes: upregulating tumor NKG2D ligands through the cGAS-STING pathway, thereby enhancing NK-cell cytotoxicity; and inducing CXCL8 secretion via the NF-κB/mTOR axis to promote NK cell recruitment to the tumor microenvironment (Kim et al., 2006; Le Bert et al., 2014; Canter et al., 2017; Walle et al., 2022).

Summary: RT synergistically activates both innate and adaptive immunity through multifaceted mechanisms. Its dose-dependent effects lay the foundation for differentiated strategies in combination with immunotherapy.

### 2.2 Mechanisms of immune suppression

While activating antitumor immunity, radiotherapy concurrently remodels the tumor microenvironment and constrains its systemic therapeutic efficacy via diverse immunosuppressive pathways. The major mechanisms encompass TGF-β signaling activation, expansion of regulatory T cells (Tregs), inhibitory reprogramming of myeloid cells, and CD8^+^ T cell exhaustion (Figures 2A–C).

**FIGURE 2:**
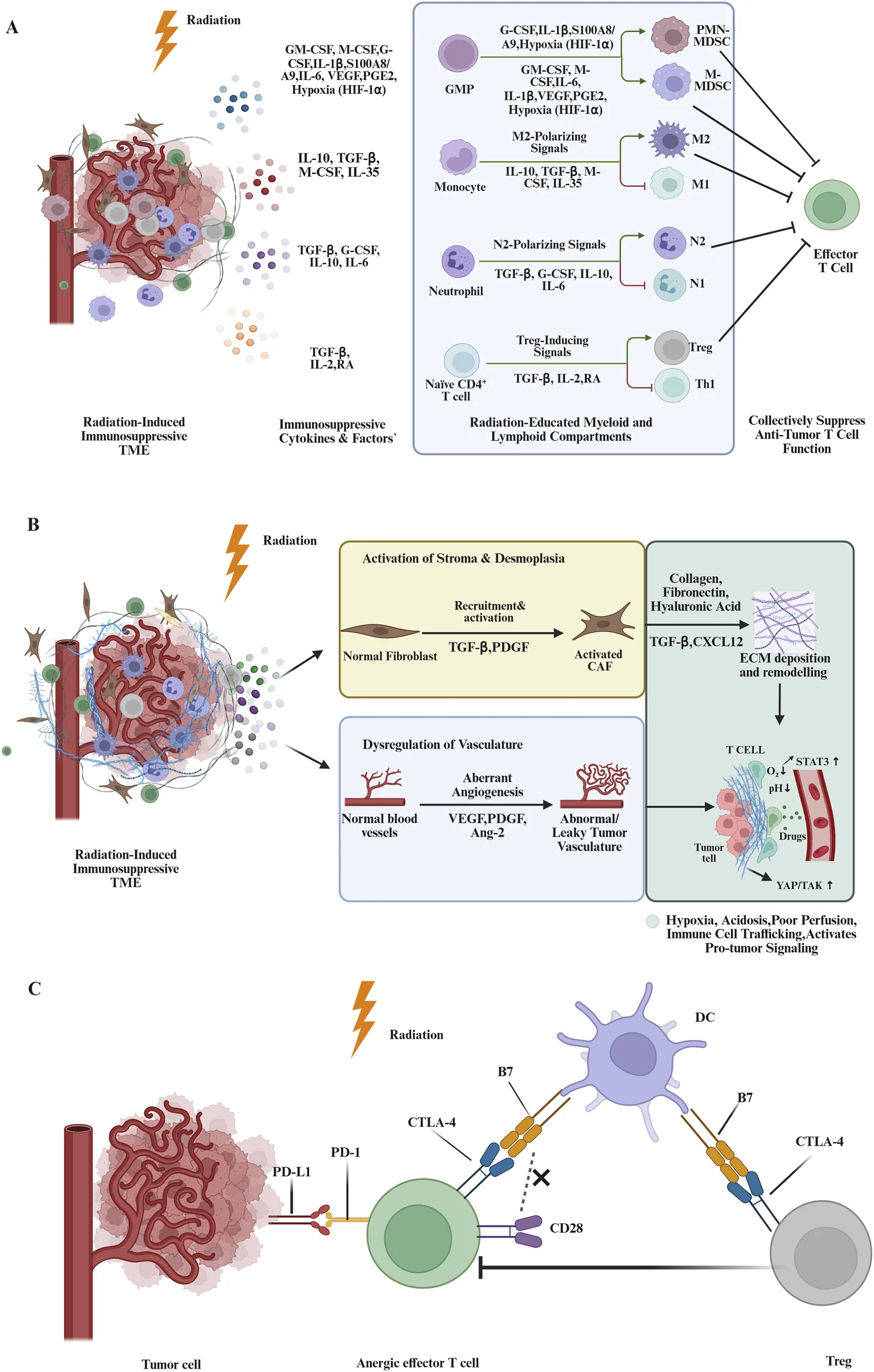
Mechanisms of radiation-induced immunosuppression in the tumor microenvironment. Radiotherapy (RT) remodels the TME into an immunosuppressive niche that fosters immune evasion through three interconnected axes, as synthesized from the literature. **(A)** Expansion of inhibitory myeloid and lymphoid populations. RT drives the expansion and functional synergy of MDSCs, M2-TAMs, N2-TANs, and Tregs, largely orchestrated by factors such as G-CSF and TGF-β, thereby suppressing effector T-cell cytotoxicity. **(B)** Stromal and vascular reprogramming. RT activates CAFs via TGF-β/PDGF signaling, promoting aberrant ECM deposition that forms a physical barrier impeding T-cell infiltration; concurrently, VEGF and Ang-2 drive disordered angiogenesis, exacerbating hypoxia, acidosis, and perfusion deficits. These physicochemical stresses sustain immunosuppression via STAT3/YAP/TAZ activation, establishing a feed-forward loop that perpetuates the hostile TME. **(C)** Checkpoint-driven T-cell dysfunction. RT imposes dual restraints on T-cell function: upregulating tumor PD-L1 to engage PD-1, and biasing antigen-presenting cells toward B7–CTLA-4 engagement over B7–CD28 co-stimulation, thereby silencing T-cell activation and inducing anergy.

#### 2.2.1 TGF-β–mediated immune suppression

Irradiation upregulates inhibitory factors such as TGF-β within the tumor microenvironment to foster an immunosuppressive network (Demaria et al., 2015; Weichselbaum et al., 2017; Rodríguez-Ruiz et al., 2018; McLaughlin et al., 2020; Charpentier et al., 2022). TGF-β directly impairs cytotoxicity of CD8^+^ T and NK cells, drives CD4^+^ T differentiation into Tregs, promotes tolerogenic polarization of DCs and macrophages, and amplifies the suppressive capacity of myeloid-derived suppressor cells (MDSCs) (Vanpouille-Box et al., 2015; Batlle and Massagué, 2019; Gunderson et al., 2020). Together with IL-10, TGF-β further facilitates Treg generation (Dennis et al., 2013; Hsu et al., 2015; Oweida et al., 2019).

Cooperating with vascular endothelial growth factor (VEGF), radiation-induced TGF-β triggers pathological stromal remodeling characterized by aberrant angiogenesis and cancer-associated fibroblast (CAF) activation, resulting in hypoxia and elevated interstitial pressure that physically and biochemically impede effector T cell (Teff) infiltration and function (Hovinga et al., 2005; Chen et al., 2014; Munn and Jain, 2019; Ware et al., 2020; Mao et al., 2021). As illustrated in Figure 2B, TGF-β and related cytokines drive pathological remodeling of stroma and vasculature.

#### 2.2.2 Expansion and immunosuppression of regulatory T cells

Radiation-mediated Treg regulation is dependent on dose and fractionation. Single high-dose irradiation markedly promotes intratumoral Treg expansion and activation, constituting a critical barrier limiting therapeutic responses (Schaue et al., 2012; Muroyama et al., 2017; Sia et al., 2021).

Tregs establish an immunosuppressive barrier via multiple routes: competitive blockade of costimulatory signals through abundant CTLA-4 expression (Muroyama et al., 2017; Nishikawa and Koyama, 2021; Sia et al., 2021); consumption of microenvironmental IL-2 to compromise Teff survival and activity (Pandiyan et al., 2007); secretion of suppressive cytokines including TGF-β and IL-10 (Flavell et al., 2010; Sawant et al., 2019); and production of immunosuppressive adenosine catalyzed by CD39/CD73 (Allard et al., 2017).

Accordingly, Treg-targeted intervention represents a promising approach to potentiate radiation-induced anti-tumor immunity.

#### 2.2.3 Inhibitory reprogramming of myeloid cells

Radiotherapy recruits and polarizes myeloid subsets toward suppressive phenotypes to build a robust immunosuppressive network (Figure 2A). It induces transcription of CCL2, CCL5 and stromal cell-derived factor-1α (SDF-1α) to recruit monocytes/macrophages and MDSCs via the CSF1/CSF1R axis (Kioi et al., 2010; Kozin et al., 2010; Xu et al., 2013; Connolly et al., 2016; Stafford et al., 2016; Kalbasi et al., 2017; Liang et al., 2017; Mondini et al., 2019).

Pathological accumulation of MDSCs post-radiation potently inhibits T and NK cell function through inhibitory cytokine secretion, metabolic substrate depletion, reactive oxygen species production and robust PD-L1 upregulation (Gabrilovich and Nagaraj, 2009; Vatner and Formenti, 2015; Veglia et al., 2021; Jiménez-Cortegana et al., 2022).

Radiation dose dynamically dictates tumor-associated macrophage (TAM) polarization: high-dose irradiation preferentially induces immunosuppressive M2-like TAMs, whereas low-dose radiation favors pro-inflammatory anti-tumor M1 polarization (Tsai et al., 2007; Klug et al., 2013; Pyonteck et al., 2013; Vatner and Formenti, 2015; Herrera et al., 2022b). Additionally, radiation drives tumor-associated neutrophils (TANs) toward pro-tumor N2 phenotype to reinforce immune suppression (Matsumoto et al., 2017; Keeley et al., 2019; Wisdom et al., 2019; Ancey et al., 2021; Faget et al., 2021). As shown in Figure 2A, radiation-derived G-CSF, GM-CSF, TGF-β and IL-10 instruct progenitor cells to differentiate into MDSCs, M2-TAMs, N2-TANs and Tregs, which collaboratively construct multi-layered immune suppression.

#### 2.2.4 Adaptive immune resistance: coordinated upregulation of immune checkpoints and progressive T cell exhaustion

Concurrent with T cell priming, radiotherapy systematically increases multiple co-inhibitory molecules to form an inhibitory molecular brake, establishing a vicious cycle coupled with progressive CD8^+^ T cell exhaustion (see Figure 2C for the main pathways).

PD-1/PD-L1 axis: Post-radiation activated CD8^+^ T cells upregulate PD-1; their secreted IFN-γ subsequently stimulates robust PD-L1 expression on tumor cells, MDSCs, TAMs and DCs, forming a self-amplifying negative feedback loop (Gros et al., 2014; Vanpouille-Box et al., 2015; Kim K. J. et al., 2017; Kordbacheh et al., 2018; Yoon et al., 2022; Chi and Nguyen, 2023).

Beyond PD-1/PD-L1, irradiation elevates alternative co-inhibitory receptors including CTLA-4, TIGIT, TIM-3 and LAG-3 in a cell-type-specific manner, collectively accelerating T cell exhaustion (Barsoumian et al., 2022; Guo et al., 2023).

Notably, circulating T cells are more radiosensitive than tissue-resident counterparts, a discrepancy closely linked to elevated TGF-β levels (Arina et al., 2019; Cytlak et al., 2022).

Brief summary: by engaging multiple co-inhibitory axes, radiotherapy renders the microenvironment susceptible to checkpoint blockade while pushing effector T cells toward irreversible exhaustion. Disrupting such adaptive resistance is essential to unlock the full immunotherapeutic potential of radiotherapy.

Table 1 systematically summarizes the dual immunomodulatory effects of radiotherapy, covering both the inhibitory cascades described above and key immune-activating events, providing mechanistic insights for subsequent combinatorial treatment development.

**TABLE 1:** Dual immunomodulatory effects of radiotherapy: Key mechanisms, regulatory features, and clinical implications.

Regulatory axis	Core mechanism/Event	Regulatory factors/Key features	Key effectors/Mediators	Primary effects	Clinical/Translational implications
Immune Activation	1. Induction of ICD	Sequential release of key DAMPs (CRT, ATP, HMGB1) synergistically activating APCs; Strong dose-dependence, more potent with SBRT	CRT, ATP, HMGB1, TAAs, dsDNA	Provides “*in situ* vaccine” antigens and adjuvant signals, priming adaptive immunity	Cornerstone of combination therapy; Assessment of ICD intensity may predict therapeutic response
	2. Activation of cGAS-STING pathway	Dynamically regulated by radiation dose and Trex1 expression levels	dsDNA → micronuclei → cGAS → STING → IFN-I	Drives potent type I interferon response; Key amplifier of DC maturation and T cell priming	Combine with STING/TLR agonists for enhanced efficacy; Optimize RT regimens to circumvent Trex1-mediated negative feedback
	3. Differential regulation of T cell recruitment	Strong dose-dependence: Intermediate/high doses upregulate chemokines/adhesion molecules optimizing homing; LDRT induces M1 macrophage polarization, providing an alternative pathway for T cell recruitment	CXCL9/10/11, ICAM-1/VCAM-1; iNOS + M1 macrophages (LDRT)	Shapes chemokine gradients and physical channels for T cell infiltration; LDRT can reverse “cold” TME	SBRT regimens (e.g., 8 Gy × 3) optimize this effect; LDRT offers a sensitization strategy for immunotherapy-insensitive tumors
	4. Activation of NK cells	Dual activation pathways: DNA damage sensing (STING) and tumor-derived chemokines (CXCL8)	NKG2D ligands (STING-dependent), CXCL8 (via NF-κB and mTOR)	Enhances innate tumor cell killing, complementing T cell responses	Provides rationale for combining RT with ICIs or adoptive NK cell therapy
	5. Enhancement of antigen processing and presentation	Upregulates antigen peptide generation via proteasome/mTOR pathways and MHC-I expression	mTOR pathway, MHC-I molecules	Increases tumor cell immunogenicity and susceptibility to T cell recognition	Inherent compatibility with therapies requiring tumor antigen recognition (e.g., ACT)
Immune uppression	1. Upregulation of immune checkpoints	Hallmark of adaptive immune resistance: Feedback-driven upregulation of classical checkpoints (PD-1/PD-L1, CTLA-4) and next-generation checkpoints (TIM-3, TIGIT, VISTA) by RT-induced IFN-γ and other inflammatory signals, with spatiotemporally dynamic expression patterns	PD-1/PD-L1,CTLA-4/B7,TIM-3,TIGIT, VISTA	Directly induces effector T cell exhaustion, blocking activation and cytotoxicity	ICIs targeting PD-1/PD-L1 and CTLA-4 have become the standard combination partners for radiotherapy; inhibitors targeting next-generation checkpoints (TIM-3, TIGIT, VISTA) are in clinical development, with preclinical studies demonstrating Combinatorial potential with RT.
​	2. TGF-β-mediated broad immunosuppression	Master immunosuppressive cytokine mediating pleiotropic downstream effects: Suppresses Teff, induces Tregs, promotes CAF activation, etc.	TGF-β	Directly suppresses CD8^+^ T cell function; Drives fibrosis and formation of suppressive microenvironment	Key therapeutic target (e.g., TGF-β inhibitors); Levels may serve as prognostic biomarker
	3. Expansion and activation of regulatory T cells	Significantly expanded after high-dose RT; Multifaceted suppression via CTLA-4-mediated competition for costimulatory signals, secretion of inhibitory factors, etc.	Tregs, CTLA-4, IL-10, TGF-β, CD39/CD73	Suppresses effector T cell and NK cell function; Depletes IL-2; Generates immunosuppressive adenosine	Targeted depletion (anti-CTLA-4) or functional inhibition (CD73 inhibitors)
	4. Pathological remodeling of myeloid suppressor networks	Comprises multiple lineages including MDSCs (PMN-, M-), M2-TAMs, N2-TANs; Recruited and polarized by various factors (CCL2, CSF1, etc.); Polarization of TAMs/TANs exhibits clear dose-dependence: Low-dose favors anti-tumor phenotypes (M1, N1), while high-dose may promote pro-tumor phenotypes (M2, N2)	MDSCs, TAMs, TANs; CCL2/CCR2, CSF1/CSF1R, Arg1, iNOS, ROS	Establishes profound immunosuppressive microenvironment; Suppresses immunity via amino acid depletion, ROS/RNS production, PD-L1 expression, etc.	Requires combination with myeloid-targeting agents (e.g., CSF-1R inhibitors, CCR2 antagonists) to deplete or reprogram these cells
	5. Exacerbation of CD8^+^ T cell exhaustion	Hallmark of adaptive immune resistance: Activated Teff secrete IFN-γ, feedback-upregulating PD-L1 on tumor cells, forming a suppressive loop	PD-1, PD-L1, IFN-γ, JAK-STAT pathway	Leads to functional impairment of tumor-specific T cells, loss of proliferation and killing capacity	ICIs (particularly anti-PD-1/PD-L1) are central to reversing this process; Emphasizes importance of concurrent or sequential ICI administration
	6. Stromal fibrosis and aberrant vascular remodeling	Synergistically driven by TGF-β and VEGF, leading to CAF activation, ECM deposition, vascular leakage, and hypoxia	CAFs, Collagen, VEGF, Hypoxia, HIF-1α	Creates physical and metabolic barriers, hindering immune cell infiltration and drug delivery	Combine with anti-angiogenic agents (e.g., bevacizumab) and stroma-targeting drugs (e.g., FAK inhibitors) to remodel the microenvironment

Abbreviations: ACT, adoptive cell therapy; APC, antigen-presenting cell; ATP, adenosine triphosphate; CAF, cancer-associated fibroblast; cGAS, cyclic GMP-AMP synthase; CRT, calreticulin; DC, dendritic cell; dsDNA, double-stranded DNA; ECM, extracellular matrix; HIF-1α, hypoxia-inducible factor 1α; HMGB1, high-mobility group box 1; ICD, immunogenic cell death; ICI, immune checkpoint inhibitor; IFN-I, type I interferon; LDRT, low-dose radiotherapy; MDSC, myeloid-derived suppressor cell; MHC-I, major histocompatibility complex class I; mTOR, mammalian target of rapamycin; NF-κB, nuclear factor kappa-B; NK, natural killer cell; SBRT, stereotactic body radiotherapy; STING, stimulator of interferon genes; TAA, tumor-associated antigen; TAM, tumor-associated macrophage; TAN, tumor-associated neutrophil; Teff, effector T cell; Treg, regulatory T cell; VEGF, vascular endothelial growth factor.

This table summarizes the dual immunomodulatory mechanisms induced by radiotherapy (RT). The specific effects and their magnitude may be influenced by RT, parameters (e.g., dose, fractionation, timing) and tumor microenvironment heterogeneity. The “Clinical/Translational Implications” column proposes rational combination strategies based on the mechanistic insights outlined above.

## 3 Combinatorial strategies: channeling radiotherapy’s “double-edged sword” toward immune activation

RT serves as an *in situ* vaccine to initiate tumor-specific immune responses, while immunotherapy “clears the obstacles” and “enhances the effect” by relieving suppression or providing costimulatory signals. Figure 3 summarizes four major immune suppression bottlenecks induced by RT and the corresponding combinatorial intervention matrices. This section systematically elucidates combination strategies centered on immune checkpoint inhibitors (ICIs) and discusses principles for optimizing RT parameters.

**FIGURE 3:**
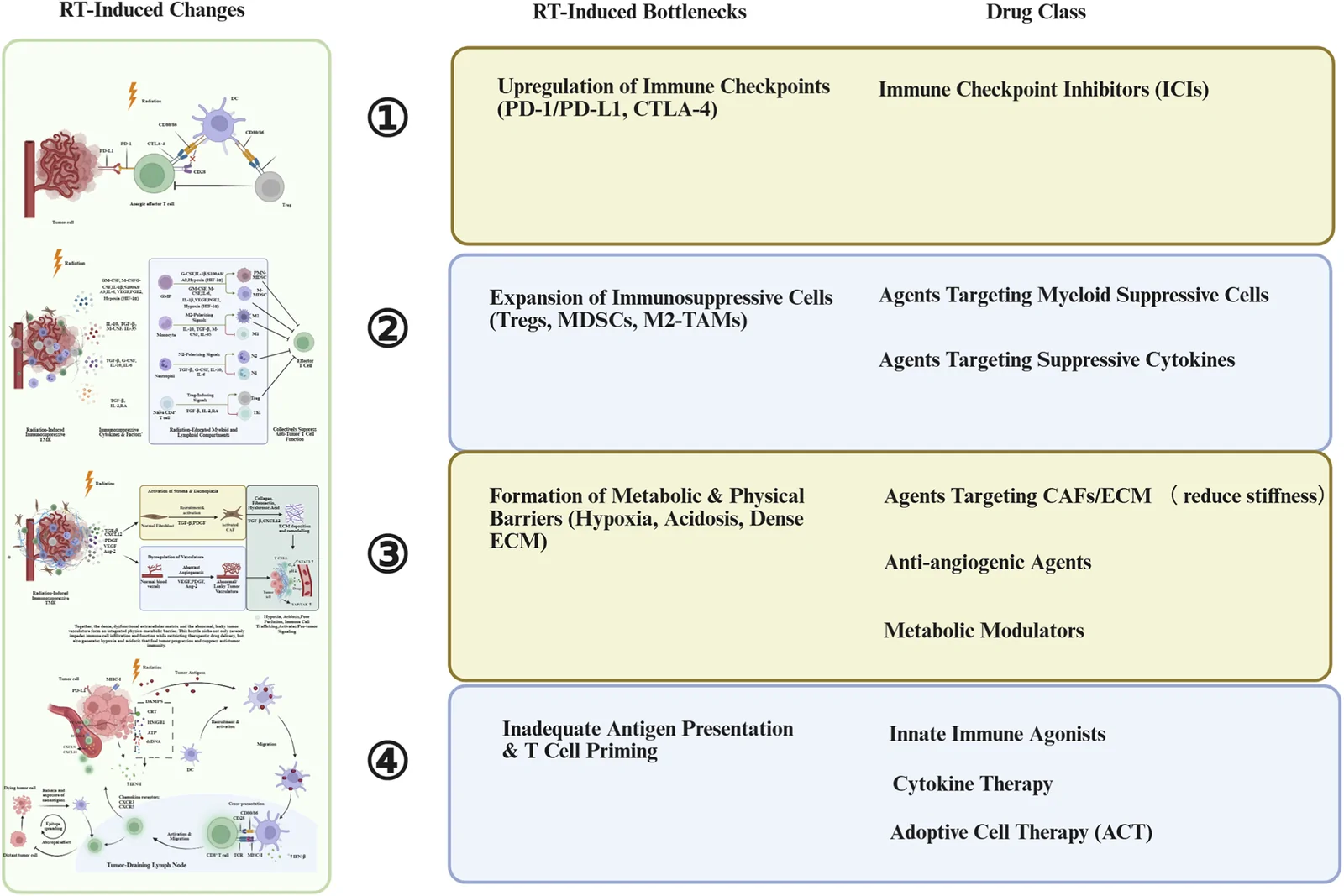
Key bottlenecks in radiotherapy-remodeled immune microenvironment and matrix of combinatorial intervention strategies. This schematic outlines four core immunosuppressive barriers induced by radiotherapy and their corresponding broad intervention categories. The left panel depicts four progressive immunosuppressive barriers triggered by Radiotherapy (RT) within the tumor microenvironment: elevated immune checkpoints, accumulation of suppressive immune cells, metabolic/physical stromal barriers, and impaired antigen presentation and T cell priming. The right panel lists major therapeutic classes; full information on representative drugs, targets and mechanisms is provided in Table 2. These combinatorial regimens reverse RT-mediated immunosuppression and strengthen systemic antitumor immunity.

### 3.1 Combination with immune checkpoint inhibitors

ICIs relieve T-cell functional suppression by blocking inhibitory pathways such as PD-1/PD-L1 and CTLA-4. When combined with RT, radiation-induced ICD releases antigens and danger signals to initiate DC-mediated T-cell responses, while ICIs block inhibitory signals during T-cell activation or effector phases, resulting in a combined benefit for antitumor immunity (Weichselbaum et al., 2017; Rodríguez-Ruiz et al., 2018; McLaughlin et al., 2020). This combined effect drives interferon-γ release, promotes vascular normalization, inhibits Tregs, and polarizes tumor-associated macrophages toward the M1 phenotype, thereby remodeling the tumor immune microenvironment and inducing abscopal effects (Castro et al., 2018).

#### 3.1.1 Combination with anti-PD-(L)1 inhibitors

RT dynamically upregulates PD-L1 expression on tumor and immune cells, thereby “sensitizing” the tumor microenvironment and enhancing T-cell responsiveness to PD-(L)1 blockade, creating favorable conditions for immunotherapy (Dovedi et al., 2014; Postow et al., 2015; Gong et al., 2017; Donlon et al., 2021; Ross et al., 2024). This combination synergizes through dual mechanisms: attenuating the suppressive function of Tregs and MDSCs while promoting clonal expansion and functional activation of tumor antigen-specific CD8^+^ T cells (Deng et al., 2014a; Park et al., 2015; Sharabi et al., 2015; Dovedi et al., 2017; Gong et al., 2017; Lan et al., 2018; Zhang and Niedermann, 2018). The RT regimen is a critical variable—hypofractionated or ablative RT (e.g., SBRT) generally outperforms conventional fractionation in augmenting immune responses, as the latter may impair efficacy by inducing MDSC accumulation (Dovedi et al., 2017; Lan et al., 2018).

Treatment outcomes exhibit significant heterogeneity, constrained by both tumor molecular features and the host microenvironment. For instance, in KRAS G12D mutant models, combination therapy activates specific lung-resident immune cells to enhance antitumor responses; however, co-mutation of KRAS with STK11/LKB1 may trigger stronger T-cell exhaustion programs, compromising therapeutic efficacy (Herter-Sprie et al., 2016; Ban et al., 2021). Beyond KRAS/STK11, KEAP1 mutations, frequently co-occurring with STK11/LKB1 loss, are associated with an immunosuppressive tumor microenvironment and poor outcomes following immune checkpoint blockade (Wang et al., 2025). PTEN loss drives constitutive PI3K-AKT pathway activation, which promotes immune evasion through reduced T-cell infiltration and increased PD-L1 expression (Peng et al., 2016). Defects in the antigen presentation machinery, including B2M mutations and HLA class I loss, constitute a major mechanism of primary resistance to immunotherapy by impairing CD8^+^ T cell recognition of tumor neoantigens (Gettinger et al., 2017; McGranahan et al., 2017). JAK1/2 mutations disrupt IFN-γ signaling, thereby abrogating the critical effector phase of antitumor immunity (Sucker et al., 2017). Furthermore, aberrant activation of the WNT/β-catenin pathway correlates with exclusion of T cells from the tumor microenvironment, representing a well-established “cold tumor” signature that limits response to both immunotherapy and radiotherapy (Huang X. et al., 2025). These findings underscore the importance of integrating a broader panel of genomic biomarkers into future predictive models for patient stratification in precision radioimmunotherapy.

#### 3.1.2 Combination with anti-CTLA-4 inhibitors

CTLA-4 is highly expressed on activated T cells and Tregs, inhibiting costimulatory signal transduction by competitively binding B7 molecules on APCs (Rudd et al., 2009; Topalian et al., 2016; Morad et al., 2021; Tekguc et al., 2021; Li G. et al., 2025). The combined benefit between RT and anti-CTLA-4 therapy manifests in three aspects: RT increases tumor-infiltrating lymphocyte numbers, providing more targets for the antibody (Formenti and Demaria, 2013); RT activates APCs and promotes costimulatory molecule expression, partially counteracting CTLA-4 inhibition (Blair et al., 2020; Storozynsky and Hitt, 2020); and RT-recruited myeloid cells can clear intratumoral Tregs via antibody-dependent cellular phagocytosis (Canter et al., 2017; Ha et al., 2019; Martineau et al., 2024). Preclinical studies indicate that the abscopal efficacy of this combination is fractionation-dependent, with regimens like 8 Gy × 3 fractions often superior to others (Dewan et al., 2009; Rudqvist et al., 2018; Yamamoto et al., 2022).

#### 3.1.3 Multiple immune checkpoint blockade

In melanoma models resistant to RT plus anti-CTLA-4 therapy, adding anti-PD-1/PD-L1 antibodies to form a triple combination reverses T-cell exhaustion and elevates complete remission rates to 80% in treatment-naïve mice (Twyman-Saint Victor et al., 2015). Furthermore, targeting other checkpoints such as TIM-3, TIGIT, and VISTA is under exploration (Kim J. E. et al., 2017; Grapin et al., 2019; Pilones et al., 2020; Patin et al., 2022). RT dynamically regulates TIGIT expression—hypofractionation (8 Gy × 3) upregulates TIGIT, whereas conventional fractionation (2 Gy × 18) downregulates it, providing a rationale for optimizing combination timing and dosing (Grapin et al., 2019).

### 3.2 Combination with alternative immunomodulatory modalities

Beyond ICIs, combinatorial regimens incorporating immune agonists, DNA damage response (DDR)-targeted agents, and multi-targeted TME modulators are under active preclinical and early-phase clinical investigation. Robust high-level clinical evidence remains lacking to validate their therapeutic efficacy; hence, these approaches are briefly summarized herein.

Building on the *in situ* vaccination effect of RT, concurrent immune agonist administration enables precise amplification of systemic antitumor immunity. Innate immune agonists targeting STING/TLR pathways and cytokines including IL-2 and IL-12 synergistically boost type I interferon responses and establish a proinflammatory TME to prime antitumor immunity (Wu et al., 2018; Olivo Pimentel et al., 2021; Pieper et al., 2021; Jagodinsky et al., 2022; Lu et al., 2024). CD40 agonists strengthen costimulatory signaling in APCs, facilitating efficient T-cell priming against RT-released tumor antigens (Liu et al., 2021). Costimulatory agonists of T cells (OX40, 4-1BB) further promote clonal expansion, effector differentiation, and long-term persistence of antigen-specific T lymphocytes (Han et al., 2022; Martin et al., 2023). Additionally, DDR inhibition exemplified by ATR blockade synergizes with RT to augment ICD and neoantigen release via cGAS-STING pathway activation (Sheng et al., 2020; Patin et al., 2022). Multi-targeted TME reprogramming strategies, including TGF-β, VEGF, CD73, IDO1, and CD47 inhibition, are also being explored for combinatorial RT regimens (Holmgaard et al., 2013; Xu et al., 2013; Lan et al., 2018; Pilones et al., 2020; Wennerberg et al., 2020; Chen et al., 2021; Han et al., 2021; Lan et al., 2021; Nishiga et al., 2022; He et al., 2023).

### 3.3 Combination with adoptive cell therapy

RT remodels the immunosuppressive TME to facilitate tumor homing, interstitial infiltration, and effector function of adoptively transferred T cells (Klug et al., 2013; Rohaan et al., 2019; Cheng et al., 2021; Qin et al., 2021). High-dose RT potentiates the susceptibility of malignant cells to adoptive T-cell-mediated killing by triggering endogenous host immune activation and upregulating death receptor expression on tumor cells (Chakraborty et al., 2003; Lai et al., 2019; Hauth et al., 2021).

Conversely, low-dose RT optimizes intratumoral T-cell delivery through normalization of aberrant vasculature and polarization of macrophages toward an antitumoral M1 phenotype (Klug et al., 2013; Laurent et al., 2023). Preclinical data demonstrate that pre-infusion low-dose total-body irradiation improves the therapeutic potency of CAR-T cells (DeSelm et al., 2018; Sugita et al., 2023; Talebi et al., 2025), whereas relevant clinical corroboration is still limited.

Table 2 provides a systematic overview of the aforementioned combinatorial RT regimens, categorized according to key immune dysregulation bottlenecks—encompassing both immunosuppression and insufficient immune activation—induced by RT. It details representative agents and their core mechanistic profiles to offer a theoretical basis for future preclinical research and clinical translation.

**TABLE 2:** Radiotherapy combined with immunotherapy: targeted strategies and representative drugs.

RT-Induced immune suppression/Activation deficit	Therapeutic target/Strategy	Combination strategy category	Representative drugs/Therapies	Primary mechanism of action
1. Upregulation of immune checkpoints	Classical immune checkpoints	Immune checkpoint inhibitors	pembrolizumab (anti-PD-1), nivolumab (anti-PD-1), durvalumab (anti-PD-L1), ipilimumab (anti-CTLA-4)	Blockade of PD-1/PD-L1 or CTLA-4 signaling, releasing T cell inhibition
	Next-generation immune checkpoints	​	cobolimab (anti-TIM-3), tiragolumab (anti-TIGIT), HMBD-002 (anti-VISTA)	Targeting coinhibitory molecules expressed on T cells or myeloid cells, synergizing with RT to enhance antitumor immunity; under clinical investigation
2. Expansion and recruitment of suppressive cells	Suppressive B cells	B cell-targeting agents	rituximab (anti-CD20)	Depletion of CD20^+^ suppressive B cells, alleviating immunosuppression mediated by IL-10, TGF-β, etc., and remodeling the tumor immune microenvironment
	Tregs	Treg-targeting agents	low-dose cyclophosphamide, ipilimumab (anti-CTLA-4, with Treg-depleting function)	Selective depletion or inhibition of Tregs, relieving their suppression on effector T cells; Ipilimumab mediates Treg clearance via ADCC
	MDSCs	MDSC-targeting agents	pexidartinib (CSF-1R inhibitor), BMS-813160 (CCR2/5 inhibitor), tivozanib (VEGFR inhibitor, with additional MDSC-inhibitory function)	Inhibition of MDSC recruitment, expansion, and immunosuppressive function
	M2-TAMs	TAM-targeting agents	pexidartinib (CSF-1R inhibitor, with dual functions of inhibiting MDSCs and promoting TAM reprogramming toward M1 phenotype); magrolimab (anti-CD47, activating macrophage phagocytosis)	Depletion of M2-TAMs or reprogramming them toward antitumor M1 phenotype
	Suppressive cytokines	Cytokine-targeting agents	galunisertib (TGF-β inhibitor), siltuximab (anti-IL-6), canakinumab (anti-IL-1β)	Blockade of TGF-β, IL-6, and other immunosuppressive signals, reversing suppressive cell polarization and function
	Adenosine pathway	CD73/adenosine-targeting agents	oleclumab (anti-CD73)	Blockade of CD73-mediated conversion of AMP to adenosine, relieving adenosine-induced suppression of T cell and NK cell function
3. Metabolic/physical barriers	Tumor vascular abnormalities	Anti-angiogenic agents	bevacizumab (anti-VEGF), Ramucirumab (anti-VEGFR2), cabozantinib (multitarget TKI)	Promotion of vascular normalization, improved perfusion, reduced hypoxia, and enhanced immune cell infiltration
	Tumor stroma/ECM	CAF/ECM-targeting agents	defactinib (FAK inhibitor), simtuzumab (LOXL2 inhibitor), PEGPH20 (hyaluronidase)	Degradation of ECM or inhibition of its crosslinking, reducing tissue stiffness and interstitial pressure, facilitating immune cell and drug infiltration
	Tumor metabolic abnormalities	Metabolic modulators	metformin (AMPK agonist), AqB013 (aquaporin inhibitor), epacadostat (IDO1 inhibitor)	Modulation of tumor metabolism, ameliorating suppressive metabolic conditions such as acidosis and amino acid depletion
4. Inadequate antigen presentation and T cell priming	Innate immune activation	Innate immune agonists	ADU-S100 (STING agonist), Poly (I:C) (TLR3 agonist), SD-101 (TLR9 agonist), Imiquimod (TLR7 agonist)	Mimicking ICD effects, activating DCs and type I interferon responses, enhancing antigen presentation
	Antigen presentation enhancement	APC-targeting agents	Selicrelumab (anti-CD40 agonist), Flt3L (Flt3 ligand)	Activation of DCs, promoting their maturation and antigen cross-presentation capacity
	T cell support and replacement	T cell costimulatory agonists	BMS-986178 (OX40 agonist), INCAGN01876 (GITR agonist)	Provision of costimulatory signals promoting antigen-specific T cell expansion, survival, and effector differentiation; GITR agonists also attenuate Treg suppressive activity
	​	Cytokine therapy	IL-2 (Aldesleukin), IL-15, IFN-α	Provision of growth and survival signals for T/NK cells, enhancing effector function
	​	Adoptive cell therapy	CAR-T, TCR-T, TIL	Direct infusion of tumor-specific T cells, bypassing host immune activation and tolerance barriers

Abbreviations: ADCC, antibody-dependent cell-mediated cytotoxicity; AMPK, AMP-activated protein kinase; APC, antigen-presenting cell; CAF, cancer-associated fibroblast; CAR-T, chimeric antigen receptor T cell; CCR, chemokine receptor; CD, cluster of differentiation; CSF-1R, colony-stimulating factor 1 receptor; DC, dendritic cell; ECM, extracellular matrix; FAK, focal adhesion kinase; ICD, immunogenic cell death; IDO1, indoleamine 2,3-dioxygenase 1; IL, interleukin; LOXL2, lysyl oxidase-like 2; MDSCs, myeloid-derived suppressor cells; NK, cell, natural killer cell; STING, stimulator of interferon genes; TAMs, tumor-associated macrophages; TCR-T, T cell receptor-engineered T cell; TIL, tumor-infiltrating lymphocyte; TKI, tyrosine kinase inhibitor; TLR, Toll-like receptor; TGF-β, transforming growth factor-β; Tregs, regulatory T cells; VEGF, vascular endothelial growth factor; VEGFR, vascular endothelial growth factor receptor.

This table systematically summarizes combination therapeutic strategies targeting radiotherapy-induced immunosuppression and activation deficits. Representative drugs/therapies encompass multiple development stages ranging from preclinical to clinical application. The listed agents are intended to illustrate strategic directions and do not represent a comprehensive inventory. Selection of specific combination regimens should consider tumor type, immune microenvironment characteristics, and radiotherapy parameters.

### 3.4 Optimization of radiotherapy dose, fractionation, and delivery techniques

Immunomodulatory effects of RT are tightly dependent on administered dose and fractionation schedules. Optimal parameter selection requires dynamic balancing of three core endpoints: direct tumoricidal cytotoxicity and antigen liberation, TME remodeling, and preservation of host lymphocytes, with individualized adjustment tailored to predefined therapeutic goals.

#### 3.4.1 Rational selection of dose and fractionation regimens

Conventional fractionated RT remains the clinical standard of care, yet it elicits modest systemic antitumor immunity and frequently drives intratumoral accumulation of immunosuppressive MDSCs and Tregs (Kachikwu et al., 2011; Dovedi et al., 2017). Nevertheless, tumor-specific T-cell responses have been detectable in patients with prostate and colorectal malignancies following conventional RT (Schaue et al., 2008; Tabi et al., 2010). Meta-analyses have validated improved local disease control and survival outcomes with altered fractionation schemes (hyperfractionation/accelerated fractionation) in head and neck squamous cell carcinoma, laying a clinical foundation for combining these regimens with immunotherapy (Lacas et al., 2017).

Ablative high-dose stereotactic body radiotherapy (SBRT) functions as a potent *in situ* tumor vaccine (Formenti and Demaria, 2012). Preclinically, SBRT enhances T-cell priming and promotes CD8^+^ T-cell infiltration as well as tumor regression in breast cancer, lung cancer and melanoma models (Lee et al., 2009; Verbrugge et al., 2012), and such effects are dose-dependent: single high-dose irradiation (e.g., 15 Gy) facilitates antigen-specific T-cell response and recruitment (Lugade et al., 2005), while prominent and sustained upregulation of MHC class I is observed at 8–10 Gy (Reits et al., 2006). Excessively escalated doses counteract immunostimulation by upregulating Trex1 expression, recruiting immunosuppressive immune cells including MDSCs and Tregs, and triggering systemic lymphotoxicity beyond a critical threshold (Connolly et al., 2016; Vanpouille-Box et al., 2017; Mattes et al., 2021). Preclinical comparisons confirm that 3 × 8 Gy hypofractionation outperforms single 20 Gy ablative dosing when combined with anti-CTLA-4, yielding superior T-cell infiltration and abscopal antitumor effects (Dewan et al., 2009). These findings underscore the primacy of an optimal therapeutic dose window (balancing immune activation versus immunosuppression) over unrestricted dose escalation for pure tumoricidal ablation.

Low-dose RT is preferentially applied for selective TME reprogramming: mild DNA damage initiates damage-associated molecular pattern signaling, shifts macrophage polarization toward the M1 phenotype, diminishes inhibitory cytokine secretion, and recruits effector lymphocytes to convert immunologically “cold” tumors into inflamed, immunocompetent lesions (Klug et al., 2013; Barsoumian et al., 2020; Yin et al., 2020; Herrera et al., 2022a; Herrera et al., 2022b). Phase I RACIN trials employing biweekly ≤1 Gy irradiation achieve durable functional T-cell infiltration and overcome acquired therapeutic resistance (Herrera et al., 2022b); ultra-low-dose total-body irradiation (0.075 Gy) induces proinflammatory cytokine release alongside NK and T-cell activation (Zhou et al., 2018). A multicenter phase II trial further establishes the safety and efficacy of low-dose RT plus atezolizumab and chemotherapy for extensive-stage small-cell lung cancer (Wang H. et al., 2024).

Collectively, the RT dose paradigm has shifted from tumoricidal dose design solely guided by normal-tissue tolerance toward immune-optimized dosing, which prioritizes equilibrium between tumor eradication and sparing of circulating lymphocytes and those residing within draining lymph nodes (Li X. et al., 2025). The guiding principle for immunoradiotherapy combination is “sufficient but not excessive” irradiation.

#### 3.4.2 Novel rationally designed radiotherapy combinations (predominantly at proof-of-concept or early exploratory stages)

Three innovative dose/spatial heterogeneity-guided RT strategies have emerged to boost immunotherapeutic efficacy, with available evidence predominantly derived from preclinical studies and phase I clinical trials.

RadScopal™: This regimen delivers ablative high-dose irradiation to index lesions while concurrently delivering low-dose RT to immunologically refractory metastatic sites, thereby converting “cold” tumors into immunocompetent lesions. Early-phase clinical trial NCT02710253 reports a 53% objective response rate among low-dose-irradiated lesions (Patel et al., 2021); preclinical triple combination of RadScopal™ plus anti-TIGIT and anti-PD-1 antibodies prolongs animal survival and suppresses metastatic dissemination (Barsoumian et al., 2022).

Spatially Fractionated Radiotherapy (SFRT)/Microbeam Radiotherapy: Heterogeneous peak-and-valley intratumoral dose distribution is constructed within target volumes to enhance antigen presentation and CD8^+^ T-cell activation, enabling synergistic abscopal disease control with ICIs (Trappetti et al., 2022; Laurent et al., 2025). When paired with checkpoint blockade, microbeam radiotherapy elevates long-term survival from 0% to 40% in preclinical tumor models (Trappetti et al., 2022).

Intratumoral Dose-Gradient RT: The same neoplasm is split into discrete low- and high-dose irradiated subregions (e.g., 2 Gy low-dose compartment versus 16 Gy high-dose compartment). Crosstalk between differentially dosed tumor compartments reprograms intratumoral CD8^+^ T cells toward highly cytotoxic, interferon-competent phenotypes. Concurrent CXCR2 antagonism abrogates RT-induced protumorigenic neutrophil infiltration and combines with anti-PD-1 to achieve a triple therapeutic benefit, substantially improving local tumor control and animal survival (Bergeron et al., 2024).

Despite robust proof-of-concept data validating their mechanistic merits, rigorous large-scale controlled clinical trials are still mandatory prior to routine implementation.

## 4 From theory to practice: clinical application and efficacy evaluation

The combinatorial mechanisms and multi-dimensional strategies outlined above provide a mechanistic basis for the clinical translation of radiotherapy combined with immunotherapy. Current evidence from phase III trials, meta-analyses, and clinical practice guidelines has established this combination as a mainstream treatment across multiple cancer types, whereas most emerging strategies are only supported by smaller-scale preliminary studies. Accordingly, this chapter focuses on regimens with well-characterized mechanisms and substantial clinical data, while briefly discussing early exploratory approaches. Key clinical studies are summarized in Supplementary Table S1.

### 4.1 Clinical evidence of abscopal effects induced by SBRT plus ICIs

SBRT combined with ICIs relies on the synergism between *in situ* vaccination and immune checkpoint blockade. Locally delivered radiotherapy elicits tumor-specific immune responses, while ICIs reverse T cell-mediated immune suppression. This combinatorial approach serves as a core clinical strategy to induce abscopal effects and achieve systemic disease control.

The clinical feasibility of this strategy was initially reported in case studies. Among patients with metastatic melanoma or non-small cell lung cancer (NSCLC) who progressed after multiple lines of systemic therapy, irradiation of isolated progressive lesions with hypofractionated radiotherapy or SBRT resulted in abscopal responses. Such phenomena were observed in both patients receiving radiotherapy alone following ICI failure and those treated with concurrent radioimmunotherapy after chemotherapy progression (Postow et al., 2012; Golden et al., 2013; Yuan et al., 2017). Subsequent studies further validated these findings. In patients with advanced melanoma progressing on ipilimumab, hypofractionated radiotherapy yielded an abscopal response rate (ARR) of 52%. Patients with abscopal responses achieved a significantly longer median overall survival (OS) than controls (22.4 vs. 8.3 months, p = 0.002) (Grimaldi et al., 2014).

Subsequent investigations revealed that for patients with metastatic melanoma or mixed solid tumors resistant to immunotherapy, the safety profile and ARR were comparable between sequential and concurrent administration of SBRT and ICIs (Tang et al., 2017; Luke et al., 2018; Sundahl et al., 2018). A phase I study reported an abscopal objective response rate (ORR) of 18% in metastatic melanoma treated with SBRT plus ipilimumab. PD-L1 upregulation after radiotherapy was identified as a major resistance mechanism, which led to the proposal of a triple regimen combining radiotherapy with anti-CTLA-4 and anti-PD-1 agents (Twyman-Saint Victor et al., 2015). A prospective cohort study enrolled patients with refractory advanced melanoma. When hypofractionated radiotherapy was administered either early or late during anti-PD-1 therapy, the ORR of both irradiated and distant non-irradiated lesions reached 36% (Roger et al., 2018).

Mechanistically, abscopal effects were positively correlated with elevated expression of IFN-γ-related genes in the tumor microenvironment, accompanied by expanded peripheral CD8^+^ T cells and upregulated activation markers such as 4-1BB (Tang et al., 2017; Luke et al., 2018). Irradiation site also influenced the magnitude of systemic immune activation, with liver irradiation inducing stronger systemic immunity than lung irradiation (Tang et al., 2017).

Accumulating evidence has validated the systemic antitumor immunity triggered by SBRT plus ICIs in metastatic NSCLC (Formenti et al., 2018; Bauml et al., 2019; Theelen et al., 2019; Chen et al., 2020; Qin et al., 2020; Welsh J. et al., 2020; Theelen et al., 2021; Bestvina et al., 2022). Multiple studies have reported an ARR ranging from 33% to 50% when SBRT targeting one or two lesions was combined with ICIs (Formenti et al., 2018; Welsh J. et al., 2020; Theelen et al., 2021). A pooled analysis of the PEMBRO-RT and MDACC trials confirmed that the combination of SBRT and pembrolizumab significantly improved ARR (41.7% vs. 19.7%), as well as progression-free survival (PFS) and OS, compared with single-agent pembrolizumab (Theelen et al., 2021). A randomized phase I/II trial demonstrated that concurrent SBRT plus pembrolizumab achieved an abscopal ORR of 38%, substantially higher than 10% in the conventional radiotherapy group. Subgroup analysis showed prominent survival benefits in patients with low PD-L1 expression (1%–49%), with median PFS prolonged from 4.6 months to 20.8 months (p = 0.004) (Welsh J. et al., 2020).

Mechanistic studies confirmed that abscopal effects were closely associated with robust expansion of neoantigen-specific CD8^+^ T cell clones in peripheral blood and tumor tissues, providing cellular evidence for the *in situ* vaccination hypothesis (Formenti et al., 2018).

Beyond the canonical T cell-dependent pathway, preclinical studies have uncovered diverse mechanisms underlying abscopal responses. For instance, combined radiotherapy and CD47 blockade induced partial T cell-independent abscopal effects via macrophage-mediated phagocytosis. In colorectal cancer models, this regimen exerted triple synergistic activity when combined with PD-1 inhibitors (Nishiga et al., 2022).

Summary: clinical evidence demonstrates that SBRT combined with ICIs can induce abscopal effects in a subset of patients across various advanced solid tumors, correlating with improved patient survival. The magnitude of abscopal responses appears to be influenced by multiple factors, including tumor type, treatment line, irradiated lesion selection, disease burden, and the patient’s immune status, underscoring the importance of precise patient stratification in clinical practice.

### 4.2 Clinical efficacy of radiotherapy–immunotherapy combinations across tumor types

The clinical efficacy of radioimmunotherapy varies considerably across tumor types. The strongest evidence base and broadest clinical experience have been accumulated in NSCLC. Gastrointestinal cancers and melanoma brain metastases have also shown promising results in clinical studies, whereas outcomes in head and neck squamous cell carcinoma (HNSCC) and lymphoma remain less definitive and require further investigation. This section therefore focuses primarily on regimens with robust supporting data from phase III trials and established clinical guidelines.

#### 4.2.1 Non-small cell lung cancer

NSCLC represents the most extensively studied disease setting for radioimmunotherapy, with effective regimens spanning the entire disease continuum. Multiple combinatorial strategies have been endorsed as standard treatments by the European Society for Medical Oncology (ESMO) and National Comprehensive Cancer Network (NCCN).

##### 4.2.1.1 Locally advanced NSCLC

The treatment landscape of unresectable stage III NSCLC has been transformed by the PACIFIC trial, which demonstrated that durvalumab consolidation after definitive concurrent chemoradiotherapy (cCRT) provides durable survival benefits in patients without disease progression. Median PFS was extended from 5.6 months to 16.9 months (HR = 0.55), and median OS from 29.1 months to 47.5 months. At 5-year follow-up, the 5-year PFS and OS rates reached 33.1% and 42.9%, respectively (Pujol, 2022; Spigel et al., 2022). This regimen has a favorable safety profile without compromising quality of life, and has been established as a global standard of care. Other PD-(L)1 inhibitors have shown comparable benefits (Durm et al., 2020; Zhou et al., 2022). Multiple studies have verified that cCRT is superior to sequential chemoradiotherapy (sCRT) and serves as the cornerstone of treatment for this patient population (Furuse et al., 1999; Aupérin et al., 2010; Liang et al., 2010; Curran et al., 2011; Postmus et al., 2017; Park et al., 2020; Daly et al., 2022). For patients intolerant to cCRT, sCRT remains a reasonable alternative (Brunelli et al., 2009; Postmus et al., 2017; Daly et al., 2022; Garassino et al., 2022).

##### 4.2.1.2 Metastatic NSCLC

In oligometastatic NSCLC, local ablative radiotherapy represented by SBRT combined with immunotherapy has shown clinical benefits. In patients receiving pembrolizumab after local ablation, median PFS reached 19.1 months and median OS 41.6 months (Bauml et al., 2019). These outcomes were further supported by the pooled analysis of the PEMBRO-RT and MDACC trials, which showed that SBRT plus pembrolizumab increased ARR and improved both PFS and OS (Theelen et al., 2021).

Tumor molecular features substantially influence the efficacy of combination regimens. Spurr et al. (Spurr et al., 2022) demonstrated that in highly aneuploid, immunologically “desert” tumors, concurrent radiotherapy plus immune checkpoint blockade achieved superior local and distant responses compared with sequential administration, translating into a survival benefit. Conversely, Huang et al. (Huang J. et al., 2025) showed that even with sequential scheduling, SBRT remodels the immune microenvironment of immunologically “cold” tumors—characterized by low tumor mutational burden (TMB), negative PD-L1 expression, or Wnt pathway mutations—through upregulation of interferon signaling and antigen processing/presentation gene sets, with oligoclonal T-cell expansion observed in long-term survivors. These findings suggest that beyond treatment sequencing, the interplay between radiation-induced immune remodeling and intrinsic genomic instability shapes therapeutic outcomes, positioning genomic instability as a predictive biomarker for radioimmunotherapy responses and providing a mechanistic rationale for precision intervention to overcome primary resistance in immunologically “desert” tumors.

##### 4.2.1.3 Early-stage NSCLC

Innovative Neoadjuvant StrategiesRadioimmunotherapy has been extended to resectable early-stage NSCLC. A single-center randomized phase II trial revealed that neoadjuvant SBRT (8 Gy × 3 fractions) plus durvalumab achieved a markedly higher major pathological response (MPR) rate than single-agent durvalumab (53.3% vs. 6.7%, OR = 16, p < 0.0001), with a complete pathological response (pCR) rate of 50% in the combination arm (Altorki et al., 2021). These findings support further investigation of this neoadjuvant approach.

#### 4.2.2 Other solid tumors

Radioimmunotherapy exhibits distinct efficacy disparities across solid malignancies, highlighting the critical roles of tumor microenvironment, disease stage and treatment sequencing.

For small cell lung cancer (SCLC), treatment strategies differ substantially between limited-stage SCLC (LS-SCLC) and extensive-stage SCLC (ES-SCLC) (Welsh J. W. et al., 2020; Perez et al., 2021; Cheng et al., 2024).

For LS-SCLC, durvalumab consolidation following cCRT has been established as a global standard of care. The phase III ADRIATIC trial conducted by Cheng et al. demonstrated that this strategy prolonged median OS to 55.9 months versus 33.4 months in the control group, and median PFS to 16.6 months versus 9.2 months (Cheng et al., 2024). This landmark improvement markedly increased long-term survival and transformed the treatment paradigm for LS-SCLC. As an early exploratory attempt, the study by Welsh et al. administered pembrolizumab concurrently with cCRT. Although an ORR of 79% and median OS of 39.5 months were observed, the regimen was associated with substantial toxicities: 41 grade 3 adverse events, with grade 4 toxicities and grade ≥3 pneumonitis each occurring in 7.5% (3/40) of patients. These findings suggest a narrower safety window for upfront concurrent radioimmunotherapy compared with post-CRT immunotherapy consolidation (Welsh J. W. et al., 2020).

By contrast, positive evidence remains limited for radioimmunotherapy in ES-SCLC. A phase II trial by Perez et al. administered thoracic radiotherapy followed by dual immunotherapy (ipilimumab plus nivolumab) in patients without progression after chemotherapy (Perez et al., 2021). While the 12-month OS rate reached 48%, grade ≥3 immune-related adverse events (irAEs) occurred in 52.4% of patients, and no improvement in 6-month PFS was observed. The unfavorable risk–benefit ratio precludes its routine clinical use, indicating that optimal timing of radiotherapy and patient selection require further refinement in ES-SCLC.

For stage II–III esophageal and gastroesophageal junction cancer, the phase III CheckMate 577 trial demonstrated that postoperative nivolumab consolidation significantly prolonged median disease-free survival (DFS) from 11.0 months to 22.4 months in patients with residual disease after neoadjuvant chemoradiotherapy. This finding confirms the value of adjuvant immunotherapy for eliminating minimal residual disease and reducing recurrence risk (Kelly et al., 2021).

Substantial evidencesupports radioimmunotherapy for melanoma brain metastases (MBM). A large-scale meta-analysis including 44 studies and 2,498 patients showed that radiotherapy combined with ICIs improved OS in patients with MBM, without increasing the incidence of grade ≥3 neurological toxicities or radiation necrosis (Anvari et al., 2023). Mechanistically, radiotherapy induces immunogenic cell death (ICD) to initiate local immune responses, and ICIs further remodel the immune microenvironment of the central nervous system, thereby eliciting systemic antitumor activity. The consistency between mechanistic research and clinical outcomes provides a solid rationale for intracranial disease control and abscopal response induction (Chandra et al., 2015; Ngiow et al., 2015; Dovedi et al., 2017; Roger et al., 2018).

In HNSCC, no consistent added benefits have been observed when combining PD-(L)1 inhibitors with either SBRT or conventional radiotherapy (Lee et al., 2021; McBride et al., 2021). These negative results indicate that the profoundly immunosuppressive microenvironment of HNSCC creates intrinsic therapeutic resistance, which cannot be overcome merely by radiation-mediated immune priming. Further mechanistic investigations and novel combinatorial approaches are urgently needed.

#### 4.2.3 Lymphoma

Individualized sequencing defines optimal radioimmunotherapy for lymphoma. ICI can be delivered concurrently with limited-field irradiation, whereas sequential immunotherapy is required for extended radiation fields to avert severe lymphodepletion. Neoadjuvant immunotherapy facilitates radiation field reduction yet blunts radiation-induced immune activation; concurrent radioimmunotherapy yields robust immunogenicity but is hampered by radiation-mediated immune suppression; adjuvant immunotherapy confers favorable safety but is compromised by heterogeneous immune reconstitution (Strati and Spiotto, 2023).

Clinical validation supports the efficacy of radioimmunotherapy across lymphoma subtypes (Witzig et al., 2002; Frank et al., 2018; Lucchini et al., 2021; Sun et al., 2022). The robust evidence comes from relapsed/refractory indolent B-cell lymphoma, where radioimmunotherapy with ^90^Y-ibritumomab tiuxetan yields superior ORR versus targeted monotherapy (80% vs. 56%) and represents an established treatment option (Witzig et al., 2002). Two retrospective cohorts further validate ICI-radiation combinations: PD-1 inhibitors combined with local radiotherapy (median 30 Gy) produce an ORR of 100% and facilitate transplant bridging in relapsed/refractory classical Hodgkin lymphoma (Lucchini et al., 2021); the sandwich chemoimmunoradiotherapy regimen achieves promising disease control for localized extranodal NK/T-cell lymphoma (Sun et al., 2022).

Collectively, optimal antitumor activity depends on precise optimization of radiation dose, target volume and treatment sequence. Multiple ongoing clinical trials (e.g., NCT05967416, NCT04827862) are underway to refine combinatorial strategies and identify predictive biomarkers.

### 4.3 Multimodal precision integrated therapy for oligometastatic disease

The treatment paradigm for oligometastatic tumors has evolved considerably. Combined local ablative radiotherapy and systemic therapy is currently the mainstream approach recommended by clinical guidelines. Radiotherapy exerts dual effects: direct local tumor ablation and immune activation via *in situ* vaccination. This strategy may help overcome tumor heterogeneity and mitigate therapeutic resistance, prolonging survival while preserving patients’ quality of life.

Based on clinical evidence, this integrated approach is categorized into two standard clinical scenarios.

### 4.4 Consolidative therapy for oligoprogression

For patients developing limited disease progression during systemic treatment, additional ablative SBRT significantly prolongs PFS. A phase II randomized controlled trial by Tsai et al. (Tsai et al., 2024) reported that adding SBRT to ongoing systemic therapy improved median PFS from 3.2 months to 7.2 months (HR = 0.53, p = 0.003). Subgroup analyses revealed marked tumor-type heterogeneity: metastatic NSCLC patients derived substantial survival benefits (10.0 months vs. 2.2 months, HR = 0.41, p = 0.004), whereas no meaningful PFS improvement was observed in patients with breast cancer (4.4 months vs. 4.2 months, HR = 0.78, p = 0.20).

### 4.5 Curative combination therapy for *de novo* oligometastatic disease

For newly diagnosed oligometastatic tumors, treatment aims shift from disease control to potential cure. The standard approach consists of curative-intent SBRT targeting all detectable metastatic lesions combined with potent systemic therapy. The European Thoracic Oncology Platform (ETOP) CHESS trial adopted a regimen of SBRT plus dual immunotherapy and chemotherapy followed by maintenance immunotherapy for treatment-naive oligometastatic NSCLC. Although final OS data remain unpublished, the encouraging ORR and PFS outcomes have demonstrated transformative therapeutic potential (Guckenberger et al., 2025).

Real-world data further support the generalizability of these strategies. The large-scale OligoCARE study confirmed that individualized radiotherapy tailored to primary tumor type, metastatic sites and prior systemic therapy has become standard clinical practice worldwide (Christ et al., 2024). This milestone indicates that the management of oligometastatic disease has entered an era of precise multimodal integration.

Summary: radioimmunotherapy has been endorsed by numerous phase III trials, meta-analyses and authoritative guidelines. It features well-understood mechanisms, robust clinical evidence and broad application in NSCLC, LS-SCLC, esophageal and gastroesophageal cancer, MBM and oligometastatic disease. Radiotherapy acts as both a local tumoricidal modality and a systemic immune modulator. Future development focusing on biomarker-driven individualized regimens will further optimize therapeutic efficacy, safety and patient quality of life.

## 5 Challenges and optimized strategies for clinical translation

Radiotherapy combined with immunotherapy is effective, yet challenges remain in safety control, administration scheduling, target delineation, and radiotherapy modality selection, necessitating evidence-based optimization.

### 5.1 Safety management: identification and balance of toxicity risks

In unresectable stage III NSCLC, phase II studies (Jabbour et al., 2021; Peters et al., 2021) demonstrated that concurrent PD-1 inhibitors plus cCRT achieved an objective response rate of approximately 70%, but were associated with a markedly higher incidence of severe and fatal adverse events compared with the PACIFIC sequential regimen; the safety of the latter has been validated in phase III trials (Antonia et al., 2017; Antonia et al., 2018). The large-scale phase III trial ECOG-ACRIN EA5181 is ongoing to compare these risk-benefit profiles (Varlotto et al., 2022). The EORTC-ESTRO OligoCare consensus (Kroeze et al., 2023) indicated that SBRT combined with dual immunotherapy or anti-angiogenic agents increases overlapping thoracic and gastrointestinal toxicities, requiring individualized assessment. The consensus recommended that definitive radiotherapy doses should not be reduced for toxicity mitigation, and adjusting the administration schedule of immunological agents is the preferred strategy for toxicity control.

### 5.2 Refined management of treatment timing

The OligoCare consensus provides recommendations for administration sequencing (Kroeze et al., 2023). Based on toxicity profiles: anti-angiogenic agents and dual immunotherapy should not be administered on the same day as radiotherapy; multikinase and BRAF/MEK inhibitors should be held for no more than 2 weeks around SBRT; a washout period of at least 1 week is required for nivolumab plus ipilimumab and anti-VEGF/EGFR monoclonal antibodies; PD-(L)1 monotherapy and anti-HER2 monoclonal antibodies can be administered concurrently with the entire radiotherapy course. Detailed recommendations are provided in Tables 3, 4.

**TABLE 3:** Summary of evidence on severe (≥Grade 3) in-field toxicity Risk with combination therapies.

Drug ID	Drug class/Specific agent	Irradiated Site/Severe toxicity incidence (≥Grade 3)	Risk level	Evidence level (OCEBM)	Key clinical implications (Evidence-based interpretation)
A1	Anti-CTLA-4 (ipilimumab)	Thoracic (12%), Abdominal (10%)	Moderate	2b	Combination with SBRT carries clear organ-specific toxicity risks (e.g., pneumonitis, hepatitis); enhanced monitoring of corresponding organ function is warranted during treatment
B1	Anti-PD-1/PD-L1 (monotherapy)	Thoracic (6%)	Low	2a	Current data demonstrate a favorable safety profile, providing a reliable safety foundation for combination with SBRT.
C1	nivolumab + ipilimumab (Dual Immunotherapy)	Thoracic (26%)	High	2a	Significant synergistic toxicity observed, indicating high risk with this combination regimen; extreme caution is required
D1	Anti-VEGF Antibody (bevacizumab)	Abdominal (12%)	Moderate	2b	Non-negligible toxicity risk exists with abdominal irradiation, primarily concerning vascular injury-related complications
E1	Anti-EGFR Antibody (cetuximab)	Head and Neck (15%)	Moderate	2a	Moderate toxicity risk present with head and neck irradiation, but data support its use under close monitoring
F1	Anti-HER2 Antibody (trastuzumab/pertuzumab)	Not reported	To be determined	–	Among available limited data, no clear signals indicate significant synergistic toxicity risk
G1	Multikinase Inhibitors (sorafenib, etc.)	Abdominal (22%)	High	2b	Data demonstrate extremely high synergistic toxicity risk; this represents one of the highest-risk combination categories
H1	BRAF and MEK Inhibitors	Bone metastases (limited data, specific incidence not reported)	Low	2b	Available limited data show no increased severe toxicity risk with SBRT combined with BRAF inhibitors for bone metastases

Abbreviations: CTLA-4, cytotoxic T-lymphocyte-associated protein 4; EGFR, epidermal growth factor receptor; HER2, human epidermal growth factor receptor 2; PD-1/PD-L1, programmed cell death protein-1/programmed death-ligand 1; SBRT, stereotactic body radiotherapy; VEGF, vascular endothelial growth factor.

1 Data source: Data in this table are based on and summarized from the systematic literature review conducted by Kroeze, et al. (2023).

2 Core definitions: “Severe in-field toxicity” refers to grade ≥3 adverse events occurring within the SBRT, irradiated field, as classified by the National Cancer Institute Common Terminology Criteria for Adverse Events. “Risk level” is defined according to incidence rates as: Low risk (0%–10%), Moderate risk (11%–20%), High risk (>20%).

3 Level of evidence: Evidence levels are assessed using the Oxford Centre for Evidence-Based Medicine (OCEBM) criteria.

4 Table value: This table systematically summarizes objective toxicity evidence for different combination regimens, serving as the scientific foundation for the subsequent expert consensus (Table 4). Drug IDs (A1-H1) provide unique identifiers linking to the corresponding consensus recommendations.

**TABLE 4:** Delphi consensus-based recommendations for timing management of combination therapies.

Drug ID	Systemic therapy class/Representative agent	Consensus on timing (Day of SBRT)	Consensus on treatment interruption	Consensus on radiotherapy dose/Fractionation adjustment
A1	Anti-CTLA-4 Inhibitors (ipilimumab)	No consensus reached	No consensus reached	No adjustment
B1	Anti-PD-1/PD-L1 Inhibitors (monotherapy)	No consensus reached	No interruption required	No adjustment
C1	nivolumab + ipilimumab	Avoid same-day administration	SBRT should not be performed within 1 week after administration of nivolumab + ipilimumab	No adjustment
D1	Anti-VEGF Antibodies (bevacizumab)	Avoid same-day administration	At least one cycle should be interrupted when combining with SBRT, and SBRT should not be performed within 1 week after anti-VEGF antibody administration	No adjustment
E1	Anti-EGFR Antibodies (cetuximab)	Avoid same-day administration	SBRT should not be performed within 1 week after anti-EGFR antibody administration	No adjustment
F1	Anti-HER2 Antibodies (trastuzumab/pertuzumab)	Same-day administration permitted	No interruption required	No adjustment
G1	Multikinase Inhibitors	Avoid same-day administration	Sunitinib and sorafenib should be interrupted for up to 2 weeks before and after SBRT	No adjustment
H1	BRAF and MEK Inhibitors	Avoid same-day administration	Interruption for up to 2 weeks before and after SBRT	No adjustment

Abbreviations: CTLA-4, cytotoxic T-lymphocyte-associated protein 4; EGFR, epidermal growth factor receptor; HER2, human epidermal growth factor receptor 2; PD-1/PD-L1, programmed cell death protein-1/programmed death-ligand 1; SBRT, stereotactic body radiotherapy; VEGF, vascular endothelial growth factor.

1 Consensus source: These recommendations are based on the international Delphi consensus reported by Kroeze, et al. (2023). The consensus process involved 28 radiation oncology and clinical oncology experts and aimed to provide operational guidance for the safe implementation of combination therapies.

2 Consensus definition: Consensus was defined as ≥75% agreement among participating experts. Items not meeting this threshold are marked as “No consensus reached.”

3 Core strategy interpretation.

Timing of administration: “Avoid same-day administration” aims to mitigate acute additive effects of treatments; “Same-day administration permitted” indicates that current evidence supports its safety.

Interruption cycle: “Interrupt one cycle” refers to omitting one planned dose of the systemic agent during the SBRT, course.

Time interval: “SBRT, should not be performed within 1 week after administration” represents a more flexible interval strategy, emphasizing maintaining at least a 1-week “washout period” between SBRT, and drug administration, without necessarily requiring omission of an entire treatment cycle.

Dose adjustment: Strong consensus (≥86% agreement) supports that SBRT, should not be dose-reduced or fractionation-modified for any drug combination. This establishes the core principle of “managing risk by adjusting systemic therapy rather than compromising radiotherapy.”

4 Table linkage: This table provides clinical operational recommendations based on the evidence presented in Table 3. Drug IDs (A1-H1) strictly correspond to those in Table 3, ensuring logical coherence from “evidence” to “recommendation.”

### 5.3 Optimization of radiotherapy targets and modalities

Multiple randomized NSCLC studies have confirmed that involved-field irradiation improves survival without compromising local control compared with elective nodal irradiation (Yuan et al., 2007; Fernandes et al., 2010; Chen et al., 2013; Yamashita et al., 2015). This preserves T cell reservoirs within uninvolved lymph nodes, sustaining systemic immune responses (Zhang and Niedermann, 2018; Zientara et al., 2022). Photon radiotherapy remains the clinical mainstream (Laurent et al., 2025). Proton radiotherapy is primarily used in pediatric and head and neck malignancies (Hu et al., 2020; Zientara et al., 2022). Carbon ion radiotherapy shows potent immunostimulatory effects preclinically (Hartmann et al., 2022) but remains investigational due to the paucity of large-scale clinical evidence (Tinganelli et al., 2015; Huang et al., 2020).

### 5.4 Future perspectives: biomarkers and precision radiotherapy

Building on phase II/III evidence, TMB, chromosomal aneuploidy, KRAS/STK11 mutations, PD-L1 expression, and peripheral IFN-γ serve as biomarkers for patient selection, while novel markers like ctDNA require further validation. In line with the OligoCare consensus, SBRT, low-dose radiotherapy, or RadScopal should be selected based on individual molecular and immune profiles. Future efforts must prioritize validating biomarker-driven strategies to transition radioimmunotherapy from empirical combination to precision medicine.

## 6 Conclusion

The combination of RT and immunotherapy has reshaped the therapeutic landscape in oncology, shifting treatment goals from local tumor ablation to systemic immune remodeling. RT acts as an *in situ* vaccine to trigger antitumor immunity via ICD, cGAS-STING pathway activation, release of danger signals, and recruitment of T and NK cells. Meanwhile, RT also induces multiple immunosuppressive events including upregulated immune checkpoints, elevated TGF-β, expansion of Tregs, recruitment of MDSCs and CD8^+^ T-cell exhaustion, which may limit durable therapeutic responses. Immunotherapy can counteract RT-induced immune suppression and maximize its immunomodulatory potential.

SBRT combined with ICIs has demonstrated robust clinical benefits such as abscopal effects and prolonged survival in multiple solid tumors including NSCLC and melanoma. The PACIFIC trial has established ICI consolidation as the standard regimen for stage III NSCLC after concurrent chemoradiotherapy. Nevertheless, treatment efficacy varies considerably due to tumor genomic features, tumor-immune microenvironment, and host immune status.

Future research should advance mechanism-guided precision combination therapy in three aspects: clarifying the immunomodulatory rules of different RT parameters, developing biomarker-based predictive models for patient stratification, and standardizing treatment schedules, radiation regimens and toxicity management via prospective trials.

RT is evolving into a versatile immune modulator. Rational combination strategies may help convert immune “cold” tumors into “hot” ones and mitigate therapeutic resistance. Ultimately, immunology-guided personalized radiotherapy holds promise for improving long-term tumor control and advancing cancer treatment.

## References

[B1] Allard B. Longhi M. S. Robson S. C. Stagg J. (2017). The ectonucleotidases CD39 and CD73: novel checkpoint inhibitor targets. Immunol. Rev. 276 (1), 121–144. 10.1111/imr.12528 28258700 PMC5338647

[B2] Altorki N. K. McGraw T. E. Borczuk A. C. Saxena A. Port J. L. Stiles B. M. (2021). Neoadjuvant durvalumab with or without stereotactic body radiotherapy in patients with early-stage non-small-cell lung cancer: a single-centre, randomised phase 2 trial. Lancet Oncol. 22 (6), 824–835. 10.1016/s1470-2045(21)00149-2 34015311

[B3] Ancey P. B. Contat C. Boivin G. Sabatino S. Pascual J. Zangger N. (2021). GLUT1 expression in tumor-associated neutrophils promotes lung cancer growth and resistance to radiotherapy. Cancer Res. 81 (9), 2345–2357. 10.1158/0008-5472.Can-20-2870 33753374 PMC8137580

[B4] Antonia S. J. Villegas A. Daniel D. Vicente D. Murakami S. Hui R. (2017). Durvalumab after chemoradiotherapy in stage III non-small-cell lung cancer. N. Engl. J. Med. 377 (20), 1919–1929. 10.1056/NEJMoa1709937 28885881

[B5] Antonia S. J. Villegas A. Daniel D. Vicente D. Murakami S. Hui R. (2018). Overall survival with durvalumab after chemoradiotherapy in stage III NSCLC. N. Engl. J. Med. 379 (24), 2342–2350. 10.1056/NEJMoa1809697 30280658

[B6] Anvari A. Sasanpour P. Kheradmardi M. R. (2023). Radiotherapy and immunotherapy in Melanoma brain metastases. Hematol. Oncol. Stem Cell Ther. 16 (1), 1–20. 10.1016/j.hemonc.2021.11.001 36634277

[B7] Apetoh L. Ghiringhelli F. Tesniere A. Obeid M. Ortiz C. Criollo A. (2007). Toll-like receptor 4-dependent contribution of the immune system to anticancer chemotherapy and radiotherapy. Nat. Med. 13 (9), 1050–1059. 10.1038/nm1622 17704786

[B8] Arina A. Beckett M. Fernandez C. Zheng W. Pitroda S. Chmura S. J. (2019). Tumor-reprogrammed resident T cells resist radiation to control tumors. Nat. Commun. 10 (1), 3959. 10.1038/s41467-019-11906-2 31477729 PMC6718618

[B9] Aupérin A. Le Péchoux C. Rolland E. Curran W. J. Furuse K. Fournel P. (2010). Meta-analysis of concomitant *versus* sequential radiochemotherapy in locally advanced non-small-cell lung cancer. J. Clin. Oncol. 28 (13), 2181–2190. 10.1200/jco.2009.26.2543 20351327

[B10] Ban Y. Markowitz G. J. Zou Y. Ramchandani D. Kraynak J. Sheng J. (2021). Radiation-activated secretory proteins of Scgb1a1(+) club cells increase the efficacy of immune checkpoint blockade in lung cancer. Nat. Cancer 2 (9), 919–931. 10.1038/s43018-021-00245-1 34917944 PMC8670735

[B11] Barsoumian H. B. Ramapriyan R. Younes A. I. Caetano M. S. Menon H. Comeaux N. I. (2020). Low-dose radiation treatment enhances systemic antitumor immune responses by overcoming the inhibitory stroma. J. Immunother. Cancer 8 (2), e000537. 10.1136/jitc-2020-000537 33106386 PMC7592253

[B12] Barsoumian H. B. Sezen D. Menon H. Younes A. I. Hu Y. He K. (2022). High plus low dose radiation strategy in combination with TIGIT and PD1 blockade to promote systemic antitumor responses. Cancers (Basel) 14 (1), 221. 10.3390/cancers14010221 35008385 PMC8750272

[B13] Batlle E. Massagué J. (2019). Transforming growth Factor-β signaling in immunity and cancer. Immunity 50 (4), 924–940. 10.1016/j.immuni.2019.03.024 30995507 PMC7507121

[B14] Bauml J. M. Mick R. Ciunci C. Aggarwal C. Davis C. Evans T. (2019). Pembrolizumab after completion of locally ablative therapy for oligometastatic non-small cell lung cancer: a phase 2 trial. JAMA Oncol. 5 (9), 1283–1290. 10.1001/jamaoncol.2019.1449 31294762 PMC6624820

[B15] Ben-Sasson S. Z. Hu-Li J. Quiel J. Cauchetaux S. Ratner M. Shapira I. (2009). IL-1 acts directly on CD4 T cells to enhance their antigen-driven expansion and differentiation. Proc. Natl. Acad. Sci. U. S. A. 106 (17), 7119–7124. 10.1073/pnas.0902745106 19359475 PMC2678417

[B16] Ben-Sasson S. Z. Hogg A. Hu-Li J. Wingfield P. Chen X. Crank M. (2013). IL-1 enhances expansion, effector function, tissue localization, and memory response of antigen-specific CD8 T cells. J. Exp. Med. 210 (3), 491–502. 10.1084/jem.20122006 23460726 PMC3600912

[B17] Bergeron P. Dos Santos M. Sitterle L. Tarlet G. Lavigne J. Liu W. (2024). Non-homogenous intratumor ionizing radiation doses synergize with PD1 and CXCR2 blockade. Nat. Commun. 15 (1), 8845. 10.1038/s41467-024-53015-9 39397001 PMC11471822

[B18] Bestvina C. M. Pointer K. B. Karrison T. Al-Hallaq H. Hoffman P. C. Jelinek M. J. (2022). A phase 1 trial of concurrent or sequential ipilimumab, nivolumab, and stereotactic body radiotherapy in patients with stage IV NSCLC study. J. Thorac. Oncol. 17 (1), 130–140. 10.1016/j.jtho.2021.08.019 34500113

[B19] Blair T. C. Bambina S. Alice A. F. Kramer G. F. Medler T. R. Baird J. R. (2020). Dendritic cell maturation defines immunological responsiveness of tumors to radiation therapy. J. Immunol. 204 (12), 3416–3424. 10.4049/jimmunol.2000194 32341058 PMC7571541

[B20] Brunelli A. Charloux A. Bolliger C. T. Rocco G. Sculier J. P. Varela G. (2009). ERS/ESTS clinical guidelines on fitness for radical therapy in lung cancer patients (surgery and chemo-radiotherapy). Eur. Respir. J. 34 (1), 17–41. 10.1183/09031936.00184308 19567600

[B21] Canter R. J. Grossenbacher S. K. Foltz J. A. Sturgill I. R. Park J. S. Luna J. I. (2017). Radiotherapy enhances natural killer cell cytotoxicity and localization in pre-clinical canine sarcomas and first-in-dog clinical trial. J. Immunother. Cancer 5 (1), 98. 10.1186/s40425-017-0305-7 29254507 PMC5735903

[B22] Castro F. Cardoso A. P. Gonçalves R. M. Serre K. Oliveira M. J. (2018). Interferon-gamma at the crossroads of tumor immune surveillance or evasion. Front. Immunol. 9, 847. 10.3389/fimmu.2018.00847 29780381 PMC5945880

[B23] Chakraborty M. Abrams S. I. Camphausen K. Liu K. Scott T. Coleman C. N. (2003). Irradiation of tumor cells up-regulates fas and enhances CTL lytic activity and CTL adoptive immunotherapy. J. Immunol. 170 (12), 6338–6347. 10.4049/jimmunol.170.12.6338 12794167

[B24] Chandra R. A. Wilhite T. J. Balboni T. A. Alexander B. M. Spektor A. Ott P. A. (2015). A systematic evaluation of abscopal responses following radiotherapy in patients with metastatic melanoma treated with ipilimumab. Oncoimmunology 4 (11), e1046028. 10.1080/2162402x.2015.1046028 26451318 PMC4589983

[B25] Charpentier M. Spada S. Van Nest S. J. Demaria S. (2022). Radiation therapy-induced remodeling of the tumor immune microenvironment. Semin. Cancer Biol. 86 (Pt 2), 737–747. 10.1016/j.semcancer.2022.04.003 35405340

[B26] Chen M. Bao Y. Ma H. L. Hu X. Wang J. Wang Y. (2013). Involved-field radiotherapy *versus* elective nodal irradiation in combination with concurrent chemotherapy for locally advanced non-small cell lung cancer: a prospective randomized study. Biomed Res. Int. 2013, 371819. 10.1155/2013/371819 23762840 PMC3666303

[B27] Chen Y. H. Pan S. L. Wang J. C. Kuo S. H. Cheng J. C. Teng C. M. (2014). Radiation-induced VEGF-C expression and endothelial cell proliferation in lung cancer. Strahlenther Onkol. 190 (12), 1154–1162. 10.1007/s00066-014-0708-z 24989178 PMC4240909

[B28] Chen D. Menon H. Verma V. Guo C. Ramapriyan R. Barsoumian H. (2020). Response and outcomes after anti-CTLA4 *versus* anti-PD1 combined with stereotactic body radiation therapy for metastatic non-small cell lung cancer: retrospective analysis of two single-institution prospective trials. J. Immunother. Cancer 8 (1), e000492. 10.1136/jitc-2019-000492 31996395 PMC7057428

[B29] Chen J. L. Pan C. K. Huang Y. S. Tsai C. Y. Wang C. W. Lin Y. L. (2021). Evaluation of antitumor immunity by a combination treatment of high-dose irradiation, anti-PDL1, and anti-angiogenic therapy in murine lung tumors. Cancer Immunol. Immunother. 70 (2), 391–404. 10.1007/s00262-020-02690-w 32761424 PMC10991177

[B30] Cheng J. N. Luo W. Sun C. Jin Z. Zeng X. Alexander P. B. (2021). Radiation-induced eosinophils improve cytotoxic T lymphocyte recruitment and response to immunotherapy. Sci. Adv. 7 (5), eabc7609. 10.1126/sciadv.abc7609 33514544 PMC7846170

[B31] Cheng Y. Spigel D. R. Cho B. C. Laktionov K. K. Fang J. Chen Y. (2024). Durvalumab after chemoradiotherapy in limited-stage small-cell lung cancer. N. Engl. J. Med. 391 (14), 1313–1327. 10.1056/NEJMoa2404873 39268857

[B32] Chi A. Nguyen N. P. (2023). Mechanistic rationales for combining immunotherapy with radiotherapy. Front. Immunol. 14, 1125905. 10.3389/fimmu.2023.1125905 37377970 PMC10291094

[B33] Christ S. M. Alongi F. Ricardi U. Scorsetti M. Livi L. Balermpas P. (2024). Cancer-specific dose and fractionation schedules in stereotactic body radiotherapy for oligometastatic disease: an interim analysis of the EORTC-ESTRO E(2)-RADIatE OligoCare study. Radiother. Oncol. 195, 110235. 10.1016/j.radonc.2024.110235 38508239

[B34] Connolly K. A. Belt B. A. Figueroa N. M. Murthy A. Patel A. Kim M. (2016). Increasing the efficacy of radiotherapy by modulating the CCR2/CCR5 chemokine axes. Oncotarget 7 (52), 86522–86535. 10.18632/oncotarget.13287 27852031 PMC5349932

[B35] Curran W. J. Jr. Paulus R. Langer C. J. Komaki R. Lee J. S. Hauser S. (2011). Sequential vs. concurrent chemoradiation for stage III non-small cell lung cancer: randomized phase III trial RTOG 9410. J. Natl. Cancer Inst. 103 (19), 1452–1460. 10.1093/jnci/djr325 21903745 PMC3186782

[B36] Cytlak U. M. Dyer D. P. Honeychurch J. Williams K. J. Travis M. A. Illidge T. M. (2022). Immunomodulation by radiotherapy in tumour control and normal tissue toxicity. Nat. Rev. Immunol. 22 (2), 124–138. 10.1038/s41577-021-00568-1 34211187

[B37] Daly M. E. Singh N. Ismaila N. Antonoff M. B. Arenberg D. A. Bradley J. (2022). Management of stage III non-small-cell lung cancer: ASCO guideline. J. Clin. Oncol. 40 (12), 1356–1384. 10.1200/jco.21.02528 34936470

[B38] Delaney G. Jacob S. Featherstone C. Barton M. (2005). The role of radiotherapy in cancer treatment: estimating optimal utilization from a review of evidence-based clinical guidelines. Cancer 104 (6), 1129–1137. 10.1002/cncr.21324 16080176

[B39] Demaria S. Ng B. Devitt M. L. Babb J. S. Kawashima N. Liebes L. (2004). Ionizing radiation inhibition of distant untreated tumors (abscopal effect) is immune mediated. Int. J. Radiat. Oncol. Biol. Phys. 58 (3), 862–870. 10.1016/j.ijrobp.2003.09.012 14967443

[B40] Demaria S. Golden E. B. Formenti S. C. (2015). Role of local radiation therapy in cancer immunotherapy. JAMA Oncol. 1 (9), 1325–1332. 10.1001/jamaoncol.2015.2756 26270858

[B41] Deng L. Liang H. Burnette B. Beckett M. Darga T. Weichselbaum R. R. (2014a). Irradiation and anti-PD-L1 treatment synergistically promote antitumor immunity in mice. J. Clin. Invest 124 (2), 687–695. 10.1172/jci67313 24382348 PMC3904601

[B42] Deng L. Liang H. Xu M. Yang X. Burnette B. Arina A. (2014b). STING-dependent cytosolic DNA sensing promotes radiation-induced type I interferon-dependent antitumor immunity in immunogenic tumors. Immunity 41 (5), 843–852. 10.1016/j.immuni.2014.10.019 25517616 PMC5155593

[B43] Dennis K. L. Blatner N. R. Gounari F. Khazaie K. (2013). Current status of interleukin-10 and regulatory T-cells in cancer. Curr. Opin. Oncol. 25 (6), 637–645. 10.1097/cco.0000000000000006 24076584 PMC4322764

[B44] DeSelm C. Palomba M. L. Yahalom J. Hamieh M. Eyquem J. Rajasekhar V. K. (2018). Low-dose radiation conditioning enables CAR T cells to mitigate antigen escape. Mol. Ther. 26 (11), 2542–2552. 10.1016/j.ymthe.2018.09.008 30415658 PMC6225039

[B45] Dewan M. Z. Galloway A. E. Kawashima N. Dewyngaert J. K. Babb J. S. Formenti S. C. (2009). Fractionated but not single-dose radiotherapy induces an immune-mediated abscopal effect when combined with anti-CTLA-4 antibody. Clin. Cancer Res. 15 (17), 5379–5388. 10.1158/1078-0432.Ccr-09-0265 19706802 PMC2746048

[B46] Diamond J. M. Vanpouille-Box C. Spada S. Rudqvist N. P. Chapman J. R. Ueberheide B. M. (2018). Exosomes shuttle TREX1-Sensitive IFN-stimulatory dsDNA from irradiated cancer cells to DCs. Cancer Immunol. Res. 6 (8), 910–920. 10.1158/2326-6066.Cir-17-0581 29907693 PMC6072562

[B47] Donlon N. E. Power R. Hayes C. Reynolds J. V. Lysaght J. (2021). Radiotherapy, immunotherapy, and the tumour microenvironment: turning an immunosuppressive milieu into a therapeutic opportunity. Cancer Lett. 502, 84–96. 10.1016/j.canlet.2020.12.045 33450360

[B48] Dovedi S. J. Adlard A. L. Lipowska-Bhalla G. McKenna C. Jones S. Cheadle E. J. (2014). Acquired resistance to fractionated radiotherapy can be overcome by concurrent PD-L1 blockade. Cancer Res. 74 (19), 5458–5468. 10.1158/0008-5472.Can-14-1258 25274032

[B49] Dovedi S. J. Cheadle E. J. Popple A. L. Poon E. Morrow M. Stewart R. (2017). Fractionated radiation therapy stimulates antitumor immunity mediated by both resident and infiltrating polyclonal T-cell populations when combined with PD-1 blockade. Clin. Cancer Res. 23 (18), 5514–5526. 10.1158/1078-0432.Ccr-16-1673 28533222

[B50] Durm G. A. Jabbour S. K. Althouse S. K. Liu Z. Sadiq A. A. Zon R. T. (2020). A phase 2 trial of consolidation pembrolizumab following concurrent chemoradiation for patients with unresectable stage III non-small cell lung cancer: Hoosier cancer research network LUN 14-179. Cancer 126 (19), 4353–4361. 10.1002/cncr.33083 32697352 PMC10865991

[B51] Faget J. Peters S. Quantin X. Meylan E. Bonnefoy N. (2021). Neutrophils in the era of immune checkpoint blockade. J. Immunother. Cancer 9 (7), e002242. 10.1136/jitc-2020-002242 34301813 PMC8728357

[B52] Fernandes A. T. Shen J. Finlay J. Mitra N. Evans T. Stevenson J. (2010). Elective nodal irradiation (ENI) vs. involved field radiotherapy (IFRT) for locally advanced non-small cell lung cancer (NSCLC): a comparative analysis of toxicities and clinical outcomes. Radiother. Oncol. 95 (2), 178–184. 10.1016/j.radonc.2010.02.007 20356642

[B53] Flavell R. A. Sanjabi S. Wrzesinski S. H. Licona-Limón P. (2010). The polarization of immune cells in the tumour environment by TGFbeta. Nat. Rev. Immunol. 10 (8), 554–567. 10.1038/nri2808 20616810 PMC3885992

[B54] Formenti S. C. Demaria S. (2012). Radiation therapy to convert the tumor into an *in situ* vaccine. Int. J. Radiat. Oncol. Biol. Phys. 84 (4), 879–880. 10.1016/j.ijrobp.2012.06.020 23078897 PMC3811126

[B55] Formenti S. C. Demaria S. (2013). Combining radiotherapy and cancer immunotherapy: a paradigm shift. J. Natl. Cancer Inst. 105 (4), 256–265. 10.1093/jnci/djs629 23291374 PMC3576324

[B56] Formenti S. C. Rudqvist N. P. Golden E. Cooper B. Wennerberg E. Lhuillier C. (2018). Radiotherapy induces responses of lung cancer to CTLA-4 blockade. Nat. Med. 24 (12), 1845–1851. 10.1038/s41591-018-0232-2 30397353 PMC6286242

[B57] Frank M. J. Reagan P. M. Bartlett N. L. Gordon L. I. Friedberg J. W. Czerwinski D. K. (2018). *In situ* vaccination with a TLR9 agonist and local low-dose radiation induces systemic responses in untreated indolent lymphoma. Cancer Discov. 8 (10), 1258–1269. 10.1158/2159-8290.Cd-18-0743 30154192 PMC6171524

[B58] Furuse K. Fukuoka M. Kawahara M. Nishikawa H. Takada Y. Kudoh S. (1999). Phase III study of concurrent *versus* sequential thoracic radiotherapy in combination with mitomycin, vindesine, and cisplatin in unresectable stage III non-small-cell lung cancer. J. Clin. Oncol. 17 (9), 2692–2699. 10.1200/jco.1999.17.9.2692 10561343

[B59] Gabrilovich D. I. Nagaraj S. (2009). Myeloid-derived suppressor cells as regulators of the immune system. Nat. Rev. Immunol. 9 (3), 162–174. 10.1038/nri2506 19197294 PMC2828349

[B60] Garassino M. C. Mazieres J. Reck M. Chouaid C. Bischoff H. Reinmuth N. (2022). Durvalumab after sequential chemoradiotherapy in stage III, unresectable NSCLC: the phase 2 PACIFIC-6 trial. J. Thorac. Oncol. 17 (12), 1415–1427. 10.1016/j.jtho.2022.07.1148 35961520

[B61] Gettinger S. Choi J. Hastings K. Truini A. Datar I. Sowell R. (2017). Impaired HLA class I antigen processing and presentation as a mechanism of acquired resistance to immune checkpoint inhibitors in lung cancer. Cancer Discov. 7 (12), 1420–1435. 10.1158/2159-8290.Cd-17-0593 29025772 PMC5718941

[B62] Ghiringhelli F. Apetoh L. Tesniere A. Aymeric L. Ma Y. Ortiz C. (2009). Activation of the NLRP3 inflammasome in dendritic cells induces IL-1beta-dependent adaptive immunity against tumors. Nat. Med. 15 (10), 1170–1178. 10.1038/nm.2028 19767732

[B63] Golden E. B. Demaria S. Schiff P. B. Chachoua A. Formenti S. C. (2013). An abscopal response to radiation and ipilimumab in a patient with metastatic non-small cell lung cancer. Cancer Immunol. Res. 1 (6), 365–372. 10.1158/2326-6066.Cir-13-0115 24563870 PMC3930458

[B64] Golden E. B. Frances D. Pellicciotta I. Demaria S. Helen Barcellos-Hoff M. Formenti S. C. (2014). Radiation fosters dose-dependent and chemotherapy-induced immunogenic cell death. Oncoimmunology 3, e28518. 10.4161/onci.28518 25071979 PMC4106151

[B65] Gong X. Li X. Jiang T. Xie H. Zhu Z. Zhou F. (2017). Combined radiotherapy and Anti-PD-L1 antibody synergistically enhances antitumor effect in non-small cell lung cancer. J. Thorac. Oncol. 12 (7), 1085–1097. 10.1016/j.jtho.2017.04.014 28478231

[B66] Grapin M. Richard C. Limagne E. Boidot R. Morgand V. Bertaut A. (2019). Optimized fractionated radiotherapy with anti-PD-L1 and anti-TIGIT: a promising new combination. J. Immunother. Cancer 7 (1), 160. 10.1186/s40425-019-0634-9 31238970 PMC6593525

[B67] Grimaldi A. M. Simeone E. Giannarelli D. Muto P. Falivene S. Borzillo V. (2014). Abscopal effects of radiotherapy on advanced melanoma patients who progressed after ipilimumab immunotherapy. Oncoimmunology 3, e28780. 10.4161/onci.28780 25083318 PMC4106166

[B68] Gros A. Robbins P. F. Yao X. Li Y. F. Turcotte S. Tran E. (2014). PD-1 identifies the patient-specific CD8^+^ tumor-reactive repertoire infiltrating human tumors. J. Clin. Invest 124 (5), 2246–2259. 10.1172/jci73639 24667641 PMC4001555

[B69] Guckenberger M. Opitz I. Dellaporta T. Curioni-Fontecedro A. Frauenfelder T. Ribi K. (2025). Multimodality treatment in synchronous oligometastatic NSCLC: analysis of the ETOP CHESS trial. Lung Cancer 204, 108553. 10.1016/j.lungcan.2025.108553 40311307

[B70] Gunderson A. J. Yamazaki T. McCarty K. Fox N. Phillips M. Alice A. (2020). TGFβ suppresses CD8(+) T cell expression of CXCR3 and tumor trafficking. Nat. Commun. 11 (1), 1749. 10.1038/s41467-020-15404-8 32273499 PMC7145847

[B71] Guo S. Yao Y. Tang Y. Xin Z. Wu D. Ni C. (2023). Radiation-induced tumor immune microenvironments and potential targets for combination therapy. Signal Transduct. Target. Ther. 8 (1), 205. 10.1038/s41392-023-01462-z PMC10199044 37208386

[B72] Ha D. Tanaka A. Kibayashi T. Tanemura A. Sugiyama D. Wing J. B. (2019). Differential control of human treg and effector T cells in tumor immunity by Fc-engineered anti-CTLA-4 antibody. Proc. Natl. Acad. Sci. U. S. A. 116 (2), 609–618. 10.1073/pnas.1812186116 30587582 PMC6329979

[B73] Han M. G. Jang B. S. Kang M. H. Na D. Kim I. A. (2021). PI3Kγδ inhibitor plus radiation enhances the antitumour immune effect of PD-1 blockade in syngenic murine breast cancer and humanised patient-derived xenograft model. Eur. J. Cancer 157, 450–463. 10.1016/j.ejca.2021.08.029 34601286

[B74] Han M. G. Wee C. W. Kang M. H. Kim M. J. Jeon S. H. Kim I. A. (2022). Combination of OX40 co-stimulation, radiotherapy, and PD-1 inhibition in a syngeneic murine triple-negative breast cancer model. Cancers (Basel) 14 (11), 2692. 10.3390/cancers14112692 35681672 PMC9179485

[B75] Hartmann L. Osen W. Eichmüller O. L. Kordaß T. Furkel J. Dickes E. (2022). Carbon ion irradiation plus CTLA4 blockade elicits therapeutic immune responses in a Murine tumor model. Cancer Lett. 550, 215928. 10.1016/j.canlet.2022.215928 36183858

[B76] Hauth F. Ho A. Y. Ferrone S. Duda D. G. (2021). Radiotherapy to enhance chimeric antigen receptor T-Cell therapeutic efficacy in solid tumors: a narrative review. JAMA Oncol. 7 (7), 1051–1059. 10.1001/jamaoncol.2021.0168 33885725 PMC8668197

[B77] He K. Barsoumian H. B. Puebla-Osorio N. Hu Y. Sezen D. Wasley M. D. (2023). Inhibition of STAT6 with antisense oligonucleotides enhances the systemic antitumor effects of radiotherapy and Anti-PD-1 in metastatic non-small cell lung cancer. Cancer Immunol. Res. 11 (4), 486–500. 10.1158/2326-6066.Cir-22-0547 36700864 PMC10099280

[B78] Hernandez C. Huebener P. Schwabe R. F. (2016). Damage-associated molecular patterns in cancer: a double-edged sword. Oncogene 35 (46), 5931–5941. 10.1038/onc.2016.104 27086930 PMC5119456

[B79] Herrera F. G. Bourhis J. Coukos G. (2017). Radiotherapy combination opportunities leveraging immunity for the next oncology practice. CA Cancer J. Clin. 67 (1), 65–85. 10.3322/caac.21358 27570942

[B80] Herrera F. G. Romero P. Coukos G. (2022a). Lighting up the tumor fire with low-dose irradiation. Trends Immunol. 43 (3), 173–179. 10.1016/j.it.2022.01.006 35105519

[B81] Herrera F. G. Ronet C. Ochoa de Olza M. Barras D. Crespo I. Andreatta M. (2022b). Low-dose radiotherapy reverses tumor immune desertification and resistance to immunotherapy. Cancer Discov. 12 (1), 108–133. 10.1158/2159-8290.Cd-21-0003 34479871 PMC9401506

[B82] Herter-Sprie G. S. Koyama S. Korideck H. Hai J. Deng J. Li Y. Y. (2016). Synergy of radiotherapy and PD-1 blockade in Kras-mutant lung cancer. JCI Insight 1 (9), e87415. 10.1172/jci.insight.87415 27699275 PMC5033933

[B83] Holmgaard R. B. Zamarin D. Munn D. H. Wolchok J. D. Allison J. P. (2013). Indoleamine 2,3-dioxygenase is a critical resistance mechanism in antitumor T cell immunotherapy targeting CTLA-4. J. Exp. Med. 210 (7), 1389–1402. 10.1084/jem.20130066 23752227 PMC3698523

[B84] Hovinga K. E. Stalpers L. J. van Bree C. Donker M. Verhoeff J. J. Rodermond H. M. (2005). Radiation-enhanced vascular endothelial growth factor (VEGF) secretion in glioblastoma multiforme cell lines-a clue to radioresistance? J. Neurooncol 74 (2), 99–103. 10.1007/s11060-004-4204-7 16193379

[B85] Hsu P. Santner-Nanan B. Hu M. Skarratt K. Lee C. H. Stormon M. (2015). IL-10 potentiates differentiation of human induced regulatory T cells *via* STAT3 and Foxo1. J. Immunol. 195 (8), 3665–3674. 10.4049/jimmunol.1402898 26363058

[B86] Hu J. Huang Q. Gao J. Guan X. Hu W. Yang J. (2020). Clinical outcomes of carbon-ion radiotherapy for patients with locoregionally recurrent nasopharyngeal carcinoma. Cancer 126 (23), 5173–5183. 10.1002/cncr.33197 32931035 PMC7693227

[B87] Huang R. X. Zhou P. K. (2020). DNA damage response signaling pathways and targets for radiotherapy sensitization in cancer. Signal Transduct. Target Ther. 5 (1), 60. 10.1038/s41392-020-0150-x 32355263 PMC7192953

[B88] Huang Y. Huang Q. Zhao J. Dong Y. Zhang L. Fang X. (2020). The impacts of different types of radiation on the CRT and PDL1 expression in tumor cells under normoxia and hypoxia. Front. Oncol. 10, 1610. 10.3389/fonc.2020.01610 32974200 PMC7466457

[B89] Huang J. Theelen W. Belcaid Z. Najjar M. van der Geest D. Singh D. (2025a). Combination of pembrolizumab and radiotherapy induces systemic antitumor immune responses in immunologically cold non-small cell lung cancer. Nat. Cancer 6 (10), 1676–1692. 10.1038/s43018-025-01018-w 40696153 PMC12559004

[B90] Huang X. Gu R. Li Z. Wang F. (2025b). Igniting cold tumors: multi-Omics-driven strategies to overcome immune evasion and restore immune surveillance. Oncol. Res. 33 (10), 2857–2902. 10.32604/or.2025.066805 41050070 PMC12494119

[B91] Jabbour S. K. Lee K. H. Frost N. Breder V. Kowalski D. M. Pollock T. (2021). Pembrolizumab plus concurrent chemoradiation therapy in patients with unresectable, locally advanced, stage III non-small cell lung cancer: the phase 2 KEYNOTE-799 nonrandomized trial. JAMA Oncol. 7 (9), 1–9. 10.1001/jamaoncol.2021.2301 34086039 PMC8446818

[B92] Jaffray D. A. (2012). Image-guided radiotherapy: from current concept to future perspectives. Nat. Rev. Clin. Oncol. 9 (12), 688–699. 10.1038/nrclinonc.2012.194 23165124

[B93] Jagodinsky J. C. Bates A. M. Clark P. A. Sriramaneni R. N. Havighurst T. C. Chakravarty I. (2022). Local TLR4 stimulation augments *in situ* vaccination induced *via* local radiation and anti-CTLA-4 checkpoint blockade through induction of CD8 T-cell independent Th1 polarization. J. Immunother. Cancer 10 (10), e005103. 10.1136/jitc-2022-005103 36192087 PMC9535200

[B94] Jiménez-Cortegana C. Galassi C. Klapp V. Gabrilovich D. I. Galluzzi L. (2022). Myeloid-derived suppressor cells and radiotherapy. Cancer Immunol. Res. 10 (5), 545–557. 10.1158/2326-6066.Cir-21-1105 35426936

[B95] Kachikwu E. L. Iwamoto K. S. Liao Y. P. DeMarco J. J. Agazaryan N. Economou J. S. (2011). Radiation enhances regulatory T cell representation. Int. J. Radiat. Oncol. Biol. Phys. 81 (4), 1128–1135. 10.1016/j.ijrobp.2010.09.034 21093169 PMC3117954

[B96] Kalbasi A. Komar C. Tooker G. M. Liu M. Lee J. W. Gladney W. L. (2017). Tumor-derived CCL2 mediates resistance to radiotherapy in pancreatic ductal adenocarcinoma. Clin. Cancer Res. 23 (1), 137–148. 10.1158/1078-0432.Ccr-16-0870 27354473 PMC5195913

[B97] Keeley T. Costanzo-Garvey D. L. Cook L. M. (2019). Unmasking the many faces of tumor-associated neutrophils and macrophages: considerations for targeting innate immune cells in cancer. Trends Cancer 5 (12), 789–798. 10.1016/j.trecan.2019.10.013 31813456

[B98] Kelly R. J. Ajani J. A. Kuzdzal J. Zander T. Van Cutsem E. Piessen G. (2021). Adjuvant nivolumab in resected esophageal or gastroesophageal junction cancer. N. Engl. J. Med. 384 (13), 1191–1203. 10.1056/NEJMoa2032125 33789008

[B99] Kim J. M. Chen D. S. (2016). Immune escape to PD-L1/PD-1 blockade: seven steps to success (or failure). Ann. Oncol. 27 (8), 1492–1504. 10.1093/annonc/mdw217 27207108

[B100] Kim J. Y. Son Y. O. Park S. W. Bae J. H. Chung J. S. Kim H. H. (2006). Increase of NKG2D ligands and sensitivity to NK cell-mediated cytotoxicity of tumor cells by heat shock and ionizing radiation. Exp. Mol. Med. 38 (5), 474–484. 10.1038/emm.2006.56 17079863

[B101] Kim J. E. Patel M. A. Mangraviti A. Kim E. S. Theodros D. Velarde E. (2017a). Combination therapy with Anti-PD-1, Anti-TIM-3, and focal radiation results in regression of murine gliomas. Clin. Cancer Res. 23 (1), 124–136. 10.1158/1078-0432.Ccr-15-1535 27358487 PMC5735836

[B102] Kim K. J. Kim J. H. Lee S. J. Lee E. J. Shin E. C. Seong J. (2017b). Radiation improves antitumor effect of immune checkpoint inhibitor in murine hepatocellular carcinoma model. Oncotarget 8 (25), 41242–41255. 10.18632/oncotarget.17168 28465485 PMC5522235

[B103] Kioi M. Vogel H. Schultz G. Hoffman R. M. Harsh G. R. Brown J. M. (2010). Inhibition of vasculogenesis, but not angiogenesis, prevents the recurrence of glioblastoma after irradiation in mice. J. Clin. Invest 120 (3), 694–705. 10.1172/jci40283 20179352 PMC2827954

[B104] Klug F. Prakash H. Huber P. E. Seibel T. Bender N. Halama N. (2013). Low-dose irradiation programs macrophage differentiation to an iNOS^+^/M1 phenotype that orchestrates effective T cell immunotherapy. Cancer Cell 24 (5), 589–602. 10.1016/j.ccr.2013.09.014 24209604

[B105] Kordbacheh T. Honeychurch J. Blackhall F. Faivre-Finn C. Illidge T. (2018). Radiotherapy and anti-PD-1/PD-L1 combinations in lung cancer: building better translational research platforms. Ann. Oncol. 29 (2), 301–310. 10.1093/annonc/mdx790 29309540

[B106] Kozin S. V. (2022). Vascular damage in tumors: a key player in stereotactic radiation therapy? Trends Cancer 8 (10), 806–819. 10.1016/j.trecan.2022.06.002 35835699

[B107] Kozin S. V. Kamoun W. S. Huang Y. Dawson M. R. Jain R. K. Duda D. G. (2010). Recruitment of myeloid but not endothelial precursor cells facilitates tumor regrowth after local irradiation. Cancer Res. 70 (14), 5679–5685. 10.1158/0008-5472.Can-09-4446 20631066 PMC2918387

[B108] Kroeze S. G. C. Pavic M. Stellamans K. Lievens Y. Becherini C. Scorsetti M. (2023). Metastases-directed stereotactic body radiotherapy in combination with targeted therapy or immunotherapy: systematic review and consensus recommendations by the EORTC-ESTRO OligoCare consortium. Lancet Oncol. 24 (3), e121–e132. 10.1016/s1470-2045(22)00752-5 36858728

[B109] Lacas B. Bourhis J. Overgaard J. Zhang Q. Grégoire V. Nankivell M. (2017). Role of radiotherapy fractionation in head and neck cancers (MARCH): an updated meta-analysis. Lancet Oncol. 18 (9), 1221–1237. 10.1016/s1470-2045(17)30458-8 28757375 PMC5737765

[B110] Lai J. Z. Zhu Y. Y. Ruan M. Chen L. Zhang Q. Y. (2019). Local irradiation sensitized tumors to adoptive T cell therapy *via* enhancing the cross-priming, homing, and cytotoxicity of antigen-specific CD8 T cells. Front. Immunol. 10, 2857. 10.3389/fimmu.2019.02857 31921127 PMC6919196

[B111] Lan J. Li R. Yin L. M. Deng L. Gui J. Chen B. Q. (2018). Targeting myeloid-derived suppressor cells and programmed death ligand 1 confers therapeutic advantage of ablative hypofractionated radiation therapy compared with conventional fractionated radiation therapy. Int. J. Radiat. Oncol. Biol. Phys. 101 (1), 74–87. 10.1016/j.ijrobp.2018.01.071 29619980

[B112] Lan Y. Moustafa M. Knoll M. Xu C. Furkel J. Lazorchak A. (2021). Simultaneous targeting of TGF-β/PD-L1 synergizes with radiotherapy by reprogramming the tumor microenvironment to overcome immune evasion. Cancer Cell 39 (10), 1388–1403.e1310. 10.1016/j.ccell.2021.08.008 34506739

[B113] Laurent P. A. Morel D. Meziani L. Depil S. Deutsch E. (2023). Radiotherapy as a means to increase the efficacy of T-cell therapy in solid tumors. Oncoimmunology 12 (1), 2158013. 10.1080/2162402x.2022.2158013 36567802 PMC9788698

[B114] Laurent P. A. André F. Bobard A. Deandreis D. Demaria S. Depil S. (2025). Pushing the boundaries of radiotherapy-immunotherapy combinations: highlights from the 7(th) immunorad conference. Oncoimmunology 14 (1), 2432726. 10.1080/2162402x.2024.2432726 39696783 PMC11660406

[B115] Le Bert N. Lam A. R. Ho S. S. Shen Y. J. Liu M. M. Gasser S. (2014). STING-dependent cytosolic DNA sensor pathways regulate NKG2D ligand expression. Oncoimmunology 3, e29259. 10.4161/onci.29259 25114832 PMC4126837

[B116] Lee Y. J. Auh S. L. Wang Y. G. Burnette B. Wang Y. Meng Y. R. (2009). Therapeutic effects of ablative radiation on local tumor require CD8^+^ T cells: changing strategies for cancer treatment. Blood 114 (3), 589–595. 10.1182/blood-2009-02-206870 19349616 PMC2713472

[B117] Lee N. Y. Ferris R. L. Psyrri A. Haddad R. I. Tahara M. Bourhis J. (2021). Avelumab plus standard-of-care chemoradiotherapy *versus* chemoradiotherapy alone in patients with locally advanced squamous cell carcinoma of the head and neck: a randomised, double-blind, placebo-controlled, multicentre, phase 3 trial. Lancet Oncol. 22 (4), 450–462. 10.1016/S1470-2045(20)30737-3 33794205

[B118] Lhuillier C. Rudqvist N. P. Yamazaki T. Zhang T. Charpentier M. Galluzzi L. (2021). Radiotherapy-exposed CD8+ and CD4+ neoantigens enhance tumor control. J. Clin. Invest 131 (5), e138740. 10.1172/jci138740 33476307 PMC7919731

[B119] Li G. Li S. Jiang Y. Chen T. An Z. (2025a). Unleashing the power of immune checkpoints: a new strategy for enhancing treg cells depletion to boost antitumor immunity. Int. Immunopharmacol. 147, 113952. 10.1016/j.intimp.2024.113952 39764997

[B120] Li X. Wang C. Yang H. Xue W. Ding Y. Wu N. (2025b). Immunotherapy and radiotherapy: an effective combination in cancer treatment. Chin. Med. J. Engl. 138 (20), 2527–2539. 10.1097/cm9.0000000000003609 40764050 PMC12537194

[B121] Liang H. Y. Zhou H. Li X. L. Yin Z. H. Guan P. Zhou B. S. (2010). Chemo-radiotherapy for advanced non-small cell lung cancer: concurrent or sequential? It's no longer the question: a systematic review. Int. J. Cancer 127 (3), 718–728. 10.1002/ijc.25087 19957329

[B122] Liang H. Deng L. Hou Y. Meng X. Huang X. Rao E. (2017). Host STING-dependent MDSC mobilization drives extrinsic radiation resistance. Nat. Commun. 8 (1), 1736. 10.1038/s41467-017-01566-5 29170400 PMC5701019

[B123] Liu H. C. Viswanath D. I. Pesaresi F. Xu Y. Zhang L. Di Trani N. (2021). Potentiating antitumor efficacy through radiation and sustained intratumoral delivery of Anti-CD40 and Anti-PDL1. Int. J. Radiat. Oncol. Biol. Phys. 110 (2), 492–506. 10.1016/j.ijrobp.2020.07.2326 32768562 PMC8547413

[B124] Lu D. Li W. Tan J. Li Y. Mao W. Zheng Y. (2024). STING agonist delivered by neutrophil membrane-coated gold nanoparticles exerts synergistic tumor inhibition with radiotherapy. ACS Appl. Mater Interfaces 16 (40), 53474–53488. 10.1021/acsami.4c09825 39316508

[B125] Lucchini E. Rusconi C. Levis M. Ricci F. Santoro A. Ricardi U. (2021). Immune checkpoint inhibitors in combination with radiotherapy as salvage treatment for relapsed/refractory classical Hodgkin lymphoma: a retrospective analysis in 12 patients. Hematol. Rep. 13 (2), 9080. 10.4081/hr.2021.9080 34221295 PMC8215529

[B126] Lugade A. A. Moran J. P. Gerber S. A. Rose R. C. Frelinger J. G. Lord E. M. (2005). Local radiation therapy of B16 melanoma tumors increases the generation of tumor antigen-specific effector cells that traffic to the tumor. J. Immunol. 174 (12), 7516–7523. 10.4049/jimmunol.174.12.7516 15944250

[B127] Luke J. J. Lemons J. M. Karrison T. G. Pitroda S. P. Melotek J. M. Zha Y. (2018). Safety and clinical activity of pembrolizumab and multisite stereotactic body radiotherapy in patients with advanced solid tumors. J. Clin. Oncol. 36 (16), 1611–1618. 10.1200/jco.2017.76.2229 29437535 PMC5978468

[B128] Lussier D. M. Alspach E. Ward J. P. Miceli A. P. Runci D. White J. M. (2021). Radiation-induced neoantigens broaden the immunotherapeutic window of cancers with low mutational loads. Proc. Natl. Acad. Sci. U. S. A. 118 (24), e2102611118. 10.1073/pnas.2102611118 34099555 PMC8214694

[B129] Mao X. Xu J. Wang W. Liang C. Hua J. Liu J. (2021). Crosstalk between cancer-associated fibroblasts and immune cells in the tumor microenvironment: new findings and future perspectives. Mol. Cancer 20 (1), 131. 10.1186/s12943-021-01428-1 34635121 PMC8504100

[B130] Martin A. L. Powell C. Nagy M. Z. Innamarato P. Powers J. Nichols D. (2023). Anti-4-1BB immunotherapy enhances systemic immune effects of radiotherapy to induce B and T cell-dependent anti-tumor immune activation and improve tumor control at unirradiated sites. Cancer Immunol. Immunother. 72 (6), 1445–1460. 10.1007/s00262-022-03325-y 36469096 PMC10992043

[B131] Martineau R. Susini S. Marabelle A. (2024). Fc effector function of immune checkpoint blocking antibodies in oncology. Immunol. Rev. 328 (1), 334–349. 10.1111/imr.13427 39663733 PMC11659940

[B132] Matsumoto Y. Mabuchi S. Kozasa K. Kuroda H. Sasano T. Yokoi E. (2017). The significance of tumor-associated neutrophil density in uterine cervical cancer treated with definitive radiotherapy. Gynecol. Oncol. 145 (3), 469–475. 10.1016/j.ygyno.2017.02.009 28552395

[B133] Matsumura S. Demaria S. (2010). Up-regulation of the pro-inflammatory chemokine CXCL16 is a common response of tumor cells to ionizing radiation. Radiat. Res. 173 (4), 418–425. 10.1667/rr1860.1 20334513 PMC2857712

[B134] Matsumura S. Wang B. Kawashima N. Braunstein S. Badura M. Cameron T. O. (2008). Radiation-induced CXCL16 release by breast cancer cells attracts effector T cells. J. Immunol. 181 (5), 3099–3107. 10.4049/jimmunol.181.5.3099 18713980 PMC2587101

[B135] Mattes M. D. Eubank T. D. Almubarak M. Wen S. Marano G. D. Jacobson G. M. (2021). A prospective trial evaluating the safety and systemic response from the concurrent use of radiation therapy with checkpoint inhibitor immunotherapy in metastatic non-small cell lung cancer. Clin. Lung Cancer 22 (4), 268–273. 10.1016/j.cllc.2021.01.012 33608212 PMC8310528

[B136] McBride S. Sherman E. Tsai C. J. Baxi S. Aghalar J. Eng J. (2021). Randomized phase II trial of nivolumab with stereotactic body radiotherapy *versus* nivolumab alone in metastatic head and neck squamous cell carcinoma. J. Clin. Oncol. 39 (1), 30–37. 10.1200/jco.20.00290 32822275 PMC8462641

[B137] McGranahan N. Rosenthal R. Hiley C. T. Rowan A. J. Watkins T. B. K. Wilson G. A. (2017). Allele-specific HLA loss and immune escape in lung cancer evolution. Cell 171 (6), 1259–1271.e1211. 10.1016/j.cell.2017.10.001 29107330 PMC5720478

[B138] McLaughlin M. Patin E. C. Pedersen M. Wilkins A. Dillon M. T. Melcher A. A. (2020). Inflammatory microenvironment remodelling by tumour cells after radiotherapy. Nat. Rev. Cancer 20 (4), 203–217. 10.1038/s41568-020-0246-1 32161398

[B139] Mondini M. Loyher P. L. Hamon P. Gerbé de Thoré M. Laviron M. Berthelot K. (2019). CCR2-Dependent recruitment of tregs and monocytes following radiotherapy is associated with TNFα-Mediated resistance. Cancer Immunol. Res. 7 (3), 376–387. 10.1158/2326-6066.Cir-18-0633 30696630

[B140] Morad G. Helmink B. A. Sharma P. Wargo J. A. (2021). Hallmarks of response, resistance, and toxicity to immune checkpoint blockade. Cell 184 (21), 5309–5337. 10.1016/j.cell.2021.09.020 34624224 PMC8767569

[B141] Morisada M. Clavijo P. E. Moore E. Sun L. Chamberlin M. Van Waes C. (2018). PD-1 blockade reverses adaptive immune resistance induced by high-dose hypofractionated but not low-dose daily fractionated radiation. Oncoimmunology 7 (3), e1395996. 10.1080/2162402x.2017.1395996 29399393 PMC5790397

[B142] Munn L. L. Jain R. K. (2019). Vascular regulation of antitumor immunity. Science 365 (6453), 544–545. 10.1126/science.aaw7875 31395771 PMC7321824

[B143] Muroyama Y. Nirschl T. R. Kochel C. M. Lopez-Bujanda Z. Theodros D. Mao W. (2017). Stereotactic radiotherapy increases functionally suppressive regulatory T cells in the tumor microenvironment. Cancer Immunol. Res. 5 (11), 992–1004. 10.1158/2326-6066.Cir-17-0040 28970196 PMC5793220

[B144] Nagarsheth N. Wicha M. S. Zou W. (2017). Chemokines in the cancer microenvironment and their relevance in cancer immunotherapy. Nat. Rev. Immunol. 17 (9), 559–572. 10.1038/nri.2017.49 28555670 PMC5731833

[B145] Ngiow S. F. McArthur G. A. Smyth M. J. (2015). Radiotherapy complements immune checkpoint blockade. Cancer Cell 27 (4), 437–438. 10.1016/j.ccell.2015.03.015 25873170

[B146] Ngwa W. Irabor O. C. Schoenfeld J. D. Hesser J. Demaria S. Formenti S. C. (2018). Using immunotherapy to boost the abscopal effect. Nat. Rev. Cancer 18 (5), 313–322. 10.1038/nrc.2018.6 29449659 PMC5912991

[B147] Nishiga Y. Drainas A. P. Baron M. Bhattacharya D. Barkal A. A. Ahrari Y. (2022). Radiotherapy in combination with CD47 blockade elicits a macrophage-mediated abscopal effect. Nat. Cancer 3 (11), 1351–1366. 10.1038/s43018-022-00456-0 36411318 PMC9701141

[B148] Nishikawa H. Koyama S. (2021). Mechanisms of regulatory T cell infiltration in tumors: implications for innovative immune precision therapies. J. Immunother. Cancer 9 (7), e002591. 10.1136/jitc-2021-002591 34330764 PMC8327843

[B149] Olivo Pimentel V. Marcus D. van der Wiel A. M. Lieuwes N. G. Biemans R. Lieverse R. I. (2021). Releasing the brakes of tumor immunity with anti-PD-L1 and pushing its accelerator with L19-IL2 cures poorly immunogenic tumors when combined with radiotherapy. J. Immunother. Cancer 9 (3), e001764. 10.1136/jitc-2020-001764 PMC7944996 33688020

[B150] Oweida A. J. Darragh L. Phan A. Binder D. Bhatia S. Mueller A. (2019). STAT3 modulation of regulatory T cells in response to radiation therapy in head and neck cancer. J. Natl. Cancer Inst. 111 (12), 1339–1349. 10.1093/jnci/djz036 30863843 PMC6910208

[B151] Pandiyan P. Zheng L. Ishihara S. Reed J. Lenardo M. J. (2007). CD4+CD25+Foxp3+ regulatory T cells induce cytokine deprivation-mediated apoptosis of effector CD4+ T cells. Nat. Immunol. 8 (12), 1353–1362. 10.1038/ni1536 17982458

[B152] Park S. S. Dong H. Liu X. Harrington S. M. Krco C. J. Grams M. P. (2015). PD-1 restrains radiotherapy-induced abscopal effect. Cancer Immunol. Res. 3 (6), 610–619. 10.1158/2326-6066.Cir-14-0138 25701325 PMC4827718

[B153] Park K. Vansteenkiste J. Lee K. H. Pentheroudakis G. Zhou C. Prabhash K. (2020). Pan-asian adapted ESMO clinical practice guidelines for the management of patients with locally-advanced unresectable non-small-cell lung cancer: a KSMO-ESMO initiative endorsed by CSCO, ISMPO, JSMO, MOS, SSO and TOS. Ann. Oncol. 31 (2), 191–201. 10.1016/j.annonc.2019.10.026 31959336

[B154] Patel R. R. He K. Barsoumian H. B. Chang J. Y. Tang C. Verma V. (2021). High-dose irradiation in combination with non-ablative low-dose radiation to treat metastatic disease after progression on immunotherapy: results of a phase II trial. Radiother. Oncol. 162, 60–67. 10.1016/j.radonc.2021.06.037 34237343 PMC11905861

[B155] Patin E. C. Dillon M. T. Nenclares P. Grove L. Soliman H. Leslie I. (2022). Harnessing radiotherapy-induced NK-cell activity by combining DNA damage-response inhibition and immune checkpoint blockade. J. Immunother. Cancer 10 (3), e004306. 10.1136/jitc-2021-004306 35314434 PMC8938703

[B156] Peng W. Chen J. Q. Liu C. Malu S. Creasy C. Tetzlaff M. T. (2016). Loss of PTEN promotes resistance to T cell-mediated immunotherapy. Cancer Discov. 6 (2), 202–216. 10.1158/2159-8290.Cd-15-0283 26645196 PMC4744499

[B157] Perez B. A. Kim S. Wang M. Karimi A. M. Powell C. Li J. (2021). Prospective single-arm phase 1 and 2 study: Ipilimumab and nivolumab with thoracic radiation therapy after platinum chemotherapy in extensive-stage small cell lung cancer. Int. J. Radiat. Oncol. Biol. Phys. 109 (2), 425–435. 10.1016/j.ijrobp.2020.09.031 33002543 PMC8465780

[B158] Peters S. Felip E. Dafni U. Tufman A. Guckenberger M. Álvarez R. (2021). Progression-free and overall survival for concurrent nivolumab with standard concurrent chemoradiotherapy in locally advanced stage IIIA-B NSCLC: results from the European thoracic oncology platform NICOLAS phase II trial (European thoracic oncology platform 6-14). J. Thorac. Oncol. 16 (2), 278–288. 10.1016/j.jtho.2020.10.129 33188912

[B159] Pieper A. A. Rakhmilevich A. L. Spiegelman D. V. Patel R. B. Birstler J. Jin W. J. (2021). Combination of radiation therapy, bempegaldesleukin, and checkpoint blockade eradicates advanced solid tumors and metastases in mice. J. Immunother. Cancer 9 (6), e002715. 10.1136/jitc-2021-002715 34172518 PMC8237721

[B160] Pilones K. A. Hensler M. Daviaud C. Kraynak J. Fucikova J. Galluzzi L. (2020). Converging focal radiation and immunotherapy in a preclinical model of triple negative breast cancer: contribution of VISTA blockade. Oncoimmunology 9 (1), 1830524. 10.1080/2162402x.2020.1830524 33150045 PMC7583495

[B161] Postmus P. E. Kerr K. M. Oudkerk M. Senan S. Waller D. A. Vansteenkiste J. (2017). Early and locally advanced non-small-cell lung cancer (NSCLC): ESMO clinical practice guidelines for diagnosis, treatment and follow-up. Ann. Oncol. 28 (Suppl. l_4), iv1–iv21. 10.1093/annonc/mdx222 28881918

[B162] Postow M. A. Callahan M. K. Barker C. A. Yamada Y. Yuan J. Kitano S. (2012). Immunologic correlates of the abscopal effect in a patient with melanoma. N. Engl. J. Med. 366 (10), 925–931. 10.1056/NEJMoa1112824 22397654 PMC3345206

[B163] Postow M. A. Callahan M. K. Wolchok J. D. (2015). Immune checkpoint blockade in cancer therapy. J. Clin. Oncol. 33 (17), 1974–1982. 10.1200/jco.2014.59.4358 25605845 PMC4980573

[B164] Pujol J. L. (2022). Durvalumab induces sustained survival benefit after concurrent chemoradiotherapy in stage III non-small-cell lung cancer. J. Clin. Oncol. 40 (12), 1271–1274. 10.1200/jco.22.00204 35239424

[B165] Pyonteck S. M. Akkari L. Schuhmacher A. J. Bowman R. L. Sevenich L. Quail D. F. (2013). CSF-1R inhibition alters macrophage polarization and blocks glioma progression. Nat. Med. 19 (10), 1264–1272. 10.1038/nm.3337 24056773 PMC3840724

[B166] Qin A. Rengan R. Lee S. Santana-Davila R. Goulart B. H. L. Martins R. (2020). A pilot study of atezolizumab plus hypofractionated image guided radiation therapy for the treatment of advanced non-small cell lung cancer. Int. J. Radiat. Oncol. Biol. Phys. 108 (1), 170–177. 10.1016/j.ijrobp.2019.10.047 31756415

[B167] Qin V. M. Haynes N. M. D'Souza C. Neeson P. J. Zhu J. J. (2021). CAR-T plus radiotherapy: a promising combination for immunosuppressive tumors. Front. Immunol. 12, 813832. 10.3389/fimmu.2021.813832 35095911 PMC8790144

[B168] Reits E. A. Hodge J. W. Herberts C. A. Groothuis T. A. Chakraborty M. Wansley E. K. (2006). Radiation modulates the peptide repertoire, enhances MHC class I expression, and induces successful antitumor immunotherapy. J. Exp. Med. 203 (5), 1259–1271. 10.1084/jem.20052494 16636135 PMC3212727

[B169] Rodríguez-Ruiz M. E. Vanpouille-Box C. Melero I. Formenti S. C. Demaria S. (2018). Immunological mechanisms responsible for radiation-induced abscopal effect. Trends Immunol. 39 (8), 644–655. 10.1016/j.it.2018.06.001 30001871 PMC6326574

[B170] Roger A. Finet A. Boru B. Beauchet A. Mazeron J. J. Otzmeguine Y. (2018). Efficacy of combined hypo-fractionated radiotherapy and anti-PD-1 monotherapy in difficult-to-treat advanced melanoma patients. Oncoimmunology 7 (7), e1442166. 10.1080/2162402x.2018.1442166 30034949 PMC6053300

[B171] Rohaan M. W. Wilgenhof S. Haanen J. (2019). Adoptive cellular therapies: the current landscape. Virchows Arch. 474 (4), 449–461. 10.1007/s00428-018-2484-0 30470934 PMC6447513

[B172] Ross H. J. Kozono D. Wang X. F. Urbanic J. J. Williams T. M. Nelson G. D. (2024). Atezolizumab before and after chemoradiation for unresectable stage III non-small cell lung cancer: a phase II nonrandomized controlled trial. JAMA Oncol. 10 (9), 1212–1219. 10.1001/jamaoncol.2024.1897 39052256 PMC11273282

[B173] Rudd C. E. Taylor A. Schneider H. (2009). CD28 and CTLA-4 coreceptor expression and signal transduction. Immunol. Rev. 229 (1), 12–26. 10.1111/j.1600-065X.2009.00770.x 19426212 PMC4186963

[B174] Rudqvist N. P. Pilones K. A. Lhuillier C. Wennerberg E. Sidhom J. W. Emerson R. O. (2018). Radiotherapy and CTLA-4 blockade shape the TCR repertoire of tumor-infiltrating T cells. Cancer Immunol. Res. 6 (2), 139–150. 10.1158/2326-6066.Cir-17-0134 29180535 PMC6020019

[B175] Salomon N. Vascotto F. Selmi A. Vormehr M. Quinkhardt J. Bukur T. (2020). A liposomal RNA vaccine inducing neoantigen-specific CD4(+) T cells augments the antitumor activity of local radiotherapy in mice. Oncoimmunology 9 (1), 1771925. 10.1080/2162402x.2020.1771925 32923128 PMC7458669

[B176] Sawant D. V. Yano H. Chikina M. Zhang Q. Liao M. Liu C. (2019). Adaptive plasticity of IL-10(+) and IL-35(+) T(reg) cells cooperatively promotes tumor T cell exhaustion. Nat. Immunol. 20 (6), 724–735. 10.1038/s41590-019-0346-9 30936494 PMC6531353

[B177] Schaue D. Comin-Anduix B. Ribas A. Zhang L. Goodglick L. Sayre J. W. (2008). T-cell responses to survivin in cancer patients undergoing radiation therapy. Clin. Cancer Res. 14 (15), 4883–4890. 10.1158/1078-0432.Ccr-07-4462 18676762 PMC2748652

[B178] Schaue D. Ratikan J. A. Iwamoto K. S. McBride W. H. (2012). Maximizing tumor immunity with fractionated radiation. Int. J. Radiat. Oncol. Biol. Phys. 83 (4), 1306–1310. 10.1016/j.ijrobp.2011.09.049 22208977 PMC3337972

[B179] Sharabi A. B. Nirschl C. J. Kochel C. M. Nirschl T. R. Francica B. J. Velarde E. (2015). Stereotactic radiation therapy augments antigen-specific PD-1-Mediated antitumor immune responses *via* cross-presentation of tumor antigen. Cancer Immunol. Res. 3 (4), 345–355. 10.1158/2326-6066.Cir-14-0196 25527358 PMC4390444

[B180] Sheng H. Huang Y. Xiao Y. Zhu Z. Shen M. Zhou P. (2020). ATR inhibitor AZD6738 enhances the antitumor activity of radiotherapy and immune checkpoint inhibitors by potentiating the tumor immune microenvironment in hepatocellular carcinoma. J. Immunother. Cancer 8 (1), e000340. 10.1136/jitc-2019-000340 32461345 PMC7254123

[B181] Sia J. Hagekyriakou J. Chindris I. Albarakati H. Leong T. Schlenker R. (2021). Regulatory T cells shape the differential impact of radiation dose-fractionation schedules on host innate and adaptive antitumor immune defenses. Int. J. Radiat. Oncol. Biol. Phys. 111 (2), 502–514. 10.1016/j.ijrobp.2021.05.014 34023423

[B182] Spigel D. R. Faivre-Finn C. Gray J. E. Vicente D. Planchard D. Paz-Ares L. (2022). Five-year survival outcomes from the PACIFIC trial: durvalumab after chemoradiotherapy in stage III non-small-cell lung cancer. J. Clin. Oncol. 40 (12), 1301–1311. 10.1200/jco.21.01308 35108059 PMC9015199

[B183] Spurr L. F. Martinez C. A. Kang W. Chen M. Zha Y. Hseu R. (2022). Highly aneuploid non-small cell lung cancer shows enhanced responsiveness to concurrent radiation and immune checkpoint blockade. Nat. Cancer 3 (12), 1498–1512. 10.1038/s43018-022-00467-x 36443406

[B184] Stafford J. H. Hirai T. Deng L. Chernikova S. B. Urata K. West B. L. (2016). Colony stimulating factor 1 receptor inhibition delays recurrence of glioblastoma after radiation by altering myeloid cell recruitment and polarization. Neuro Oncol. 18 (6), 797–806. 10.1093/neuonc/nov272 26538619 PMC4864255

[B185] Storozynsky Q. Hitt M. M. (2020). The impact of radiation-induced DNA damage on cGAS-STING-Mediated immune responses to cancer. Int. J. Mol. Sci. 21 (22), 8877. 10.3390/ijms21228877 33238631 PMC7700321

[B186] Strati P. Spiotto M. T. (2023). Incorporating immunotherapy with radiotherapy for lymphomas. Lymphatics 1 (3), 273–286. 10.3390/lymphatics1030018 39917366 PMC11800356

[B187] Sucker A. Zhao F. Pieper N. Heeke C. Maltaner R. Stadtler N. (2017). Acquired IFNγ resistance impairs anti-tumor immunity and gives rise to T-cell-resistant melanoma lesions. Nat. Commun. 8, 15440. 10.1038/ncomms15440 28561041 PMC5460020

[B188] Sugita M. Yamazaki T. Alhomoud M. Martinet J. Latouche J. B. Golden E. (2023). Radiation therapy improves CAR T cell activity in acute lymphoblastic leukemia. Cell Death Dis. 14 (5), 305. 10.1038/s41419-023-05829-6 37142568 PMC10160073

[B189] Sun P. Wang Y. Yang H. Chen C. Nie M. Sun X. Q. (2022). Combination of Anti-PD-1 antibody, anlotinib and pegaspargase “sandwich” with radiotherapy in localized natural Killer/T cell lymphoma. Front. Immunol. 13, 766200. 10.3389/fimmu.2022.766200 35237257 PMC8882643

[B190] Sundahl N. De Wolf K. Kruse V. Meireson A. Reynders D. Goetghebeur E. (2018). Phase 1 dose escalation trial of ipilimumab and stereotactic body radiation therapy in metastatic melanoma. Int. J. Radiat. Oncol. Biol. Phys. 100 (4), 906–915. 10.1016/j.ijrobp.2017.11.029 29485070

[B191] Tabi Z. Spary L. K. Coleman S. Clayton A. Mason M. D. Staffurth J. (2010). Resistance of CD45RA- T cells to apoptosis and functional impairment, and activation of tumor-antigen specific T cells during radiation therapy of prostate cancer. J. Immunol. 185 (2), 1330–1339. 10.4049/jimmunol.1000488 20548027

[B192] Talebi F. Gregucci F. Ahmed J. Ben Chetrit N. B D. B. Chan T. A. (2025). Updates on radiotherapy-immunotherapy combinations: proceedings of 8th annual ImmunoRad conference. Oncoimmunology 14 (1), 2507856. 10.1080/2162402x.2025.2507856 PMC12101595 40401900

[B193] Tang C. Welsh J. W. de Groot P. Massarelli E. Chang J. Y. Hess K. R. (2017). Ipilimumab with stereotactic ablative radiation therapy: phase I results and immunologic correlates from peripheral T cells. Clin. Cancer Res. 23 (6), 1388–1396. 10.1158/1078-0432.Ccr-16-1432 27649551 PMC5355002

[B194] Tekguc M. Wing J. B. Osaki M. Long J. Sakaguchi S. (2021). Treg-expressed CTLA-4 depletes CD80/CD86 by trogocytosis, releasing free PD-L1 on antigen-presenting cells. Proc. Natl. Acad. Sci. U. S. A. 118 (30), e2023739118. 10.1073/pnas.2023739118 34301886 PMC8325248

[B195] Thariat J. Hannoun-Levi J. M. Sun Myint A. Vuong T. Gérard J. P. (2013). Past, present, and future of radiotherapy for the benefit of patients. Nat. Rev. Clin. Oncol. 10 (1), 52–60. 10.1038/nrclinonc.2012.203 23183635

[B196] Theelen W. Peulen H. M. U. Lalezari F. van der Noort V. de Vries J. F. Aerts J. (2019). Effect of pembrolizumab after stereotactic body radiotherapy vs pembrolizumab alone on tumor response in patients with advanced non-small cell lung cancer: results of the PEMBRO-RT phase 2 randomized clinical trial. JAMA Oncol. 5 (9), 1276–1282. 10.1001/jamaoncol.2019.1478 31294749 PMC6624814

[B197] Theelen W. Chen D. Verma V. Hobbs B. P. Peulen H. M. U. Aerts J. (2021). Pembrolizumab with or without radiotherapy for metastatic non-small-cell lung cancer: a pooled analysis of two randomised trials. Lancet Respir. Med. 9 (5), 467–475. 10.1016/s2213-2600(20)30391-x 33096027

[B198] Tinganelli W. Durante M. Hirayama R. Krämer M. Maier A. Kraft-Weyrather W. (2015). Kill-painting of hypoxic tumours in charged particle therapy. Sci. Rep. 5, 17016. 10.1038/srep17016 26596243 PMC4657060

[B199] Topalian S. L. Taube J. M. Anders R. A. Pardoll D. M. (2016). Mechanism-driven biomarkers to guide immune checkpoint blockade in cancer therapy. Nat. Rev. Cancer 16 (5), 275–287. 10.1038/nrc.2016.36 27079802 PMC5381938

[B200] Trappetti V. Potez M. Fernandez-Palomo C. Volarevic V. Shintani N. Pellicioli P. (2022). Microbeam radiation therapy controls local growth of radioresistant melanoma and treats out-of-field locoregional metastasis. Int. J. Radiat. Oncol. Biol. Phys. 114 (3), 478–493. 10.1016/j.ijrobp.2022.06.090 35934161

[B201] Tsai C. S. Chen F. H. Wang C. C. Huang H. L. Jung S. M. Wu C. J. (2007). Macrophages from irradiated tumors express higher levels of iNOS, arginase-I and COX-2, and promote tumor growth. Int. J. Radiat. Oncol. Biol. Phys. 68 (2), 499–507. 10.1016/j.ijrobp.2007.01.041 17398016

[B202] Tsai C.J. Yang J. T. Shaverdian N. Patel J. Shepherd A. F. Eng J. (2024). Standard-of-care systemic therapy with or without stereotactic body radiotherapy in patients with oligoprogressive breast cancer or non-small-cell lung cancer (consolidative Use of Radiotherapy to Block [CURB] oligoprogression): an open-label, randomised, controlled, phase 2 study. Lancet 403 (10422), 171–182. 10.1016/s0140-6736(23)01857-3 38104577 PMC10880046

[B203] Twyman-Saint Victor C. Rech A. J. Maity A. Rengan R. Pauken K. E. Stelekati E. (2015). Radiation and dual checkpoint blockade activate non-redundant immune mechanisms in cancer. Nature 520 (7547), 373–377. 10.1038/nature14292 25754329 PMC4401634

[B204] Vanpouille-Box C. Diamond J. M. Pilones K. A. Zavadil J. Babb J. S. Formenti S. C. (2015). TGFβ is a master regulator of radiation therapy-induced antitumor immunity. Cancer Res. 75 (11), 2232–2242. 10.1158/0008-5472.Can-14-3511 25858148 PMC4522159

[B205] Vanpouille-Box C. Alard A. Aryankalayil M. J. Sarfraz Y. Diamond J. M. Schneider R. J. (2017). DNA exonuclease Trex1 regulates radiotherapy-induced tumour immunogenicity. Nat. Commun. 8, 15618. 10.1038/ncomms15618 28598415 PMC5472757

[B206] Vanpouille-Box C. Demaria S. Formenti S. C. Galluzzi L. (2018). Cytosolic DNA sensing in organismal tumor control. Cancer Cell 34 (3), 361–378. 10.1016/j.ccell.2018.05.013 30216189

[B207] Varlotto J. M. Sun Z. Ky B. Upshaw J. Fitzgerald T. J. Diehn M. (2022). A review of concurrent chemo/radiation, immunotherapy, radiation planning, and biomarkers for locally advanced non-small cell lung cancer and their role in the development of ECOG-ACRIN EA5181. Clin. Lung Cancer 23 (7), 547–560. 10.1016/j.cllc.2022.06.005 35882620

[B208] Vatner R. E. Formenti S. C. (2015). Myeloid-derived cells in tumors: effects of radiation. Semin. Radiat. Oncol. 25 (1), 18–27. 10.1016/j.semradonc.2014.07.008 25481262

[B209] Veglia F. Sanseviero E. Gabrilovich D. I. (2021). Myeloid-derived suppressor cells in the era of increasing myeloid cell diversity. Nat. Rev. Immunol. 21 (8), 485–498. 10.1038/s41577-020-00490-y 33526920 PMC7849958

[B210] Verbrugge I. Hagekyriakou J. Sharp L. L. Galli M. West A. McLaughlin N. M. (2012). Radiotherapy increases the permissiveness of established mammary tumors to rejection by immunomodulatory antibodies. Cancer Res. 72 (13), 3163–3174. 10.1158/0008-5472.Can-12-0210 22570253

[B211] Walle T. Kraske J. A. Liao B. Lenoir B. Timke C. von Bohlen Und Halbach E. (2022). Radiotherapy orchestrates natural killer cell dependent antitumor immune responses through CXCL8. Sci. Adv. 8 (12), eabh4050. 10.1126/sciadv.abh4050 35319989 PMC8942354

[B212] Wang H. Yao Z. Kang K. Zhou L. Xiu W. Sun J. (2024a). Preclinical study and phase II trial of adapting low-dose radiotherapy to immunotherapy in small cell lung cancer. Med 5 (10), 1237–1254.e1239. 10.1016/j.medj.2024.06.002 38964333

[B213] Wang L. Lynch C. Pitroda S. P. Piffkó A. Yang K. Huser A. K. (2024b). Radiotherapy and immunology. J. Exp. Med. 221 (7), e20232101. 10.1084/jem.20232101 38771260 PMC11110906

[B214] Wang Y. Li C. J. Zhang T. Wang J. Y. Liu W. Y. Deng L. (2025). Circulating genomic biomarkers predict chemoradiotherapy resistance and immunotherapy response in locally advanced non-small-cell lung cancer. VIEW 6 (3), 20240145. 10.1002/VIW.20240145

[B215] Ware M. B. El-Rayes B. F. Lesinski G. B. (2020). Mirage or long-awaited oasis: reinvigorating T-cell responses in pancreatic cancer. J. Immunother. Cancer 8 (2), e001100. 10.1136/jitc-2020-001100 32843336 PMC7449491

[B216] Weichselbaum R. R. Liang H. Deng L. Fu Y. X. (2017). Radiotherapy and immunotherapy: a beneficial liaison? Nat. Rev. Clin. Oncol. 14 (6), 365–379. 10.1038/nrclinonc.2016.211 28094262

[B217] Welsh J. Menon H. Chen D. Verma V. Tang C. Altan M. (2020a). Pembrolizumab with or without radiation therapy for metastatic non-small cell lung cancer: a randomized phase I/II trial. J. Immunother. Cancer 8 (2), e001001. 10.1136/jitc-2020-001001 33051340 PMC7555111

[B218] Welsh J. W. Heymach J. V. Guo C. Menon H. Klein K. Cushman T. R. (2020b). Phase 1/2 trial of pembrolizumab and concurrent chemoradiation therapy for limited-stage SCLC. J. Thorac. Oncol. 15 (12), 1919–1927. 10.1016/j.jtho.2020.08.022 32916308 PMC10600713

[B219] Wennerberg E. Spada S. Rudqvist N. P. Lhuillier C. Gruber S. Chen Q. (2020). CD73 blockade promotes dendritic cell infiltration of irradiated tumors and tumor rejection. Cancer Immunol. Res. 8 (4), 465–478. 10.1158/2326-6066.Cir-19-0449 32047024 PMC7125001

[B220] Widau R. C. Parekh A. D. Ranck M. C. Golden D. W. Kumar K. A. Sood R. F. (2014). RIG-I-like receptor LGP2 protects tumor cells from ionizing radiation. Proc. Natl. Acad. Sci. U. S. A. 111 (4), E484–E491. 10.1073/pnas.1323253111 24434553 PMC3910628

[B221] Wisdom A. J. Hong C. S. Lin A. J. Xiang Y. Cooper D. E. Zhang J. (2019). Neutrophils promote tumor resistance to radiation therapy. Proc. Natl. Acad. Sci. U. S. A. 116 (37), 18584–18589. 10.1073/pnas.1901562116 31462499 PMC6744874

[B222] Witzig T. E. Gordon L. I. Cabanillas F. Czuczman M. S. Emmanouilides C. Joyce R. (2002). Randomized controlled trial of yttrium-90-labeled ibritumomab tiuxetan radioimmunotherapy *versus* rituximab immunotherapy for patients with relapsed or refractory low-grade, follicular, or transformed B-cell non-hodgkin's lymphoma. J. Clin. Oncol. 20 (10), 2453–2463. 10.1200/jco.2002.11.076 12011122

[B223] Woo S. R. Fuertes M. B. Corrales L. Spranger S. Furdyna M. J. Leung M. Y. (2014). STING-Dependent cytosolic DNA sensing mediates innate immune recognition of immunogenic tumors. Immunity 41 (5), 830–842. 10.1016/j.immuni.2014.10.017 25517615 PMC4384884

[B224] Wu C. J. Tsai Y. T. Lee I. J. Wu P. Y. Lu L. S. Tsao W. S. (2018). Combination of radiation and interleukin 12 eradicates large orthotopic hepatocellular carcinoma through immunomodulation of tumor microenvironment. Oncoimmunology 7 (9), e1477459. 10.1080/2162402x.2018.1477459 30228946 PMC6140549

[B225] Xu J. Escamilla J. Mok S. David J. Priceman S. West B. (2013). CSF1R signaling blockade stanches tumor-infiltrating myeloid cells and improves the efficacy of radiotherapy in prostate cancer. Cancer Res. 73 (9), 2782–2794. 10.1158/0008-5472.Can-12-3981 23418320 PMC4097014

[B226] Yamamoto J. Takahashi Y. Minami K. Tamari K. Katsuki S. Takenaka W. (2022). High dose local photon irradiation is crucial in Anti-CTLA-4 antibody therapy to enhance the abscopal response in a murine pancreatic carcinoma model. Cancers (Basel) 14 (9), 2087. 10.3390/cancers14092087 35565217 PMC9101709

[B227] Yamashita H. Takenaka R. Omori M. Imae T. Okuma K. Ohtomo K. (2015). Involved-field radiotherapy (IFRT) *versus* elective nodal irradiation (ENI) in combination with concurrent chemotherapy for 239 esophageal cancers: a single institutional retrospective study. Radiat. Oncol. 10, 171. 10.1186/s13014-015-0482-9 26269033 PMC4554303

[B228] Yin L. Xue J. Li R. Zhou L. Deng L. Chen L. (2020). Effect of low-dose radiation therapy on abscopal responses to hypofractionated radiation therapy and Anti-PD1 in mice and patients with non-small cell lung cancer. Int. J. Radiat. Oncol. Biol. Phys. 108 (1), 212–224. 10.1016/j.ijrobp.2020.05.002 32417411

[B229] Yoon Y. N. Choe M. H. Kong M. Chung W. K. Kim J. S. Lim Y. J. (2022). Dynamic alterations in PD-1/PD-L1 expression level and immune cell profiles based on radiation response status in mouse tumor model. Front. Oncol. 12, 989190. 10.3389/fonc.2022.989190 36479076 PMC9720306

[B230] Yuan S. Sun X. Li M. Yu J. Ren R. Yu Y. (2007). A randomized study of involved-field irradiation *versus* elective nodal irradiation in combination with concurrent chemotherapy for inoperable stage III nonsmall cell lung cancer. Am. J. Clin. Oncol. 30 (3), 239–244. 10.1097/01.coc.0000256691.27796.24 17551299

[B231] Yuan Z. Fromm A. Ahmed K. A. Grass G. D. Yang G. Q. Oliver D. E. (2017). Radiotherapy rescue of a nivolumab-refractory immune response in a patient with PD-L1-Negative metastatic squamous cell carcinoma of the lung. J. Thorac. Oncol. 12 (9), e135–e136. 10.1016/j.jtho.2017.04.029 28502722

[B232] Zhang X. Niedermann G. (2018). Abscopal effects with hypofractionated schedules extending into the effector phase of the tumor-specific T-Cell response. Int. J. Radiat. Oncol. Biol. Phys. 101 (1), 63–73. 10.1016/j.ijrobp.2018.01.094 29534901

[B233] Zhao Y. Zhang T. Wang Y. Lu D. Du J. Feng X. (2021). ICAM-1 orchestrates the abscopal effect of tumor radiotherapy. Proc. Natl. Acad. Sci. U. S. A. 118 (14), e2010333118. 10.1073/pnas.2010333118 33785590 PMC8040592

[B234] Zhou L. Zhang X. Li H. Niu C. Yu D. Yang G. (2018). Validating the pivotal role of the immune system in low-dose radiation-induced tumor inhibition in lewis lung cancer-bearing mice. Cancer Med. 7 (4), 1338–1348. 10.1002/cam4.1344 29479834 PMC5911597

[B235] Zhou Q. Chen M. Jiang O. Pan Y. Hu D. Lin Q. (2022). Sugemalimab *versus* placebo after concurrent or sequential chemoradiotherapy in patients with locally advanced, unresectable, stage III non-small-cell lung cancer in China (GEMSTONE-301): interim results of a randomised, double-blind, multicentre, phase 3 trial. Lancet Oncol. 23 (2), 209–219. 10.1016/s1470-2045(21)00630-6 35038429

[B236] Zientara N. Giles E. Le H. Short M. (2022). A scoping review of patient selection methods for proton therapy. J. Med. Radiat. Sci. 69 (1), 108–121. 10.1002/jmrs.540 34476905 PMC8892419

