# Supplementary material for: Beyond local control: radiotherapy–immunotherapy combinations from in situ vaccination to systemic immune remodeling
Source: Front Genet. 2026 Sep 17;17:1927654. doi: 10.3389/fgene.2026.1927654 (PMC13626747; doi:10.3389/fgene.2026.1927654)
Supplement: Supplementary file 1 [file Table1.docx]

| **Table S1. Summary of Key Clinical Trials of Radiotherapy Combined with Immunotherapy** | | | | | | | | | | | |
| --- | --- | --- | --- | --- | --- | --- | --- | --- | --- | --- | --- |
| **Study** | **Population** | **Design** | **N** | **Prior Therapy (including ICI exposure)** | **Immunotherapy** | **RT Dose & Fractionation** | **Regimen** | **No. of Irradiated Lesions** | **Efficacy Outcomes** | **≥Grade 3 TRAEs** | **Conclusion** |
| **Non-Small Cell Lung Cancer** | | | | | | | | | | | |
| Antonia SJ, et al. (2017)/(2018). N Engl J Med. | Unresectable stage III NSCLC; no progression after cCRT; WHO PS 0–1; any PD-L1 | International, multicenter, randomized, double-blind, PBO-controlled, phase III trial | 713 (709 treated; DURVA 473, PBO 236) | cCRT (platinum-based chemo + RT) completed; 26.8% received induction chemotherapy; no prior ICI exposure | DURVA 10 mg/kg IV q2w × up to 12 months | 54–66 Gy (conventional fractionation) | cCRT → DURVA consolidation (initiated 1–42 days after cCRT completion) | N/A | DURVA vs PBO  ORR 30.0% vs 17.8% (P<0.001);  mPFS 17.2 vs 5.6 mo (HR 0.51, P=0.0025);  24-month OS rate 66.3% vs 55.6% (HR 0.68) | Grade ≥3 AEs 30.5% vs 26.1%; grade ≥3 pneumonitis/radiation pneumonitis 3.4% vs 2.6%; discontinuation due to AEs 15.4% vs 9.8% | The PACIFIC trial established DURVA consolidation after cCRT as the global standard of care for unresectable stage III NSCLC, with significant OS benefit (24-month OS 66.3% vs 55.6%, HR 0.68; P=0.0025) and manageable safety; 5-year OS update (Spigel et al. 2022) confirmed durable survival benefit |
| Formenti SC, et. al. (2018). Nat Med. | Chemo-refractory metastatic NSCLC; ECOG 0–2; EGFR-mutant (enriched in PD) | Phase II, single-arm | 39 (21 evaluable) | All ≥1 prior line (chemo-refractory); 1 had prior immunotherapy (type unspecified); 41% brain metastases (treated) | IPI 3 mg/kg IV q3w × 4 cycles | 6 Gy × 5 fx (Phase I, n=10) or 9.5 Gy × 3 fx (Phase II, n=29) | Concurrent: RT (within 24h after 1st fraction) → IPI | 1 | ORR 18% (7/39; 33% in evaluable);  DCR 31%;  mOS 7.4 months (DC 20.4 months) | Toxicity profile consistent with IPI monotherapy; DLT 2 (grade 3 diarrhea/colitis) | RT + IPI induced abscopal responses in chemo-refractory NSCLC; early post-RT IFNβ increase and TCR clonal expansion were the strongest predictors; neoantigen-specific CD8 T cell expansion (KPNA2 mutation) was demonstrated in one CR patient; supports the in situ vaccine hypothesis. |
| Bauml JM, et al. (2019). JAMA Oncol. | Oligometastatic NSCLC (post-LAT); ECOG 0–1; no prior ICI | Phase II, single-arm | 45 (51 enrolled) | LAT completed (surgery, SBRT, chemoradiotherapy, etc.); no prior ICI | PEMBRO 200 mg IV q3w × 8 cycles (up to 16 if no progression) | Not fixed (varied by LAT modality) | LAT to all lesions (within 4–12 weeks) → PEMBRO (consolidation/adjuvant) | N/A | mPFS (from LAT) 19.1 months (historical control 6.6 months, P=0.005);  mOS 41.6 months;  24-month OS rate 77.5% | Grade ≥3 TRAEs 7 (15.6%);  grade ≥3 pneumonitis 3 (6.7%);  no grade 5 TRAEs;  no new safety signals | PEMBRO after LAT improved PFS in oligometastatic NSCLC (19.1 vs 6.6 months, P=0.005), with no QoL decline; warrants randomized validation. |
| Theelen W, et al. (2019). JAMA Oncol. | Advanced NSCLC; ECOG 0–1; any PD-L1 | Phase II, randomized, open-label | 76 (monotherapy 40, combination 36) | ≥1 prior chemotherapy; prior RT >6 months allowed (34%);no prior ICI | PEMBRO 200 mg IV q3w until progression | SBRT: 8 Gy × 3 fx | SBRT → PEMBRO (within 7 days post-SBRT) | 1 | combination vs monotherapy:  12-week ORR 36% vs 18% (P=0.07);  mPFS 6.6 vs 1.9 mo (HR 0.71, P=0.19);  mOS 15.9 vs 7.6 mo (HR 0.66, P=0.16);  PD-L1-negative subgroup: PFS HR 0.49 (P=0.03), OS HR 0.48 (P=0.046) | Grade ≥3 TRAEs 17% (similar between arms) | SBRT followed by PEMBRO was well tolerated; ORR doubled (36% vs 18%, P=0.07) but did not meet the prespecified endpoint; PD-L1-negative subgroup showed significant PFS/OS benefits; supports the hypothesis that SBRT may convert ‘cold’ tumors to ‘hot’; warrants larger validation trials. |
| Qin A, et al. (2020). Int J Radiat Oncol Biol Phys. | Advanced NSCLC; ECOG 0–2 | Phase II, single-arm, exploratory | 12 | Progression after platinum-based chemotherapy or treatment-naïve; no prior ICI | ATEZO 1200 mg IV q3w until progression | HIGRT: 3 fx (≥8 Gy/fx) or 5 fx (≥6 Gy/fx) | ATEZO initiated, with HIGRT added no later than C2 | 1 | ORR 25% (3/12; all PR);  DCR 50%;  mPFS 2.3 months;  mOS 6.9 months;  2 PR patients had OS >36 months | Grade 3 irAEs: 5 (41.7%);  no grade ≥4 AEs | HIGRT + ATEZO was feasible in advanced NSCLC, with ORR 25% and DCR 50%; the incidence of grade 3 irAEs was comparable to that reported with ATEZO monotherapy; limited by small sample size, warranting further validation. |
| Welsh J, et al. (2020). J Immunother Cancer. | Metastatic NSCLC; ECOG 0–1; any PD-L1 | Phase I/II, randomized, open-label | 100 (phase I: 20; phase II: 80; 72 evaluable for response) | Prior chemo/RT allowed; only 1 had prior ICI | PEMBRO 200 mg IV q3w × up to 32 cycles (phase I: dose-escalation 100–200 mg) | SBRT: 50 Gy/4 fx;  conventional RT: 45 Gy/15 fx | Concurrent: RT (started day 1) + PEMBRO  PEMBRO alone; salvage RT allowed after 2nd dose in monotherapy arm | 1–2 (mostly 1) | Overall ORR 22% (combination) vs 25% (monotherapy), P=0.99;  out-of-field ORR best with SBRT+ICI (38%), conventional RT+ICI only 10%;  low PD-L1 (1-49%) mPFS 20.8 mo (combination) vs 4.6 mo (monotherapy), P=0.001 | No grade 5 TRAEs; phase I grade 3: 30% (6/20); phase II grade 3: 21% (17/80); 2 grade 4 events (MI, VT) in SBRT + ICI arm | Concurrent immunoradiotherapy was safe; overall ORR/PFS showed no significant differences; low PD-L1 subgroup showed significant PFS benefit with RT (20.8 vs 4.6 mo, P=0.001); SBRT better preserved lymphocytes than conventional RT; warrants larger biomarker-driven trials |
| Durm GA, et al. (2020). Cancer. | Unresectable stage III NSCLC; no progression after cCRT; any PD-L1 | Phase II, single-arm (vs historical control) | 93 (92 evaluable for efficacy) | cCRT (platinum-based chemo + RT)completed; no prior ICI | PEMBRO 200 mg IV q3w × up to 12 months; median 13.5 cycles | 59.4–66.6 Gy (conventional fractionation) | cCRT → PEMBRO consolidation (initiated 28–56 days after CRT completion) | N/A | mTMDD 30.7 mo (vs cCRT historical control 12 mo, P<0.0001);  mPFS 18.7 mo;  mOS 35.8 mo;  1-/2-/3-year OS rates 81.2%/62.0%/48.5%;  PD-L1 not predictive | Pneumonia ≥ grade 2 17.2% (≥ grade 3: 6.5%);  grade ≥3 TRAEs 12.9% | Consolidation PEMBRO after cCRT improved TMDD, PFS, and OS vs historical controls; outcomes similar to PACIFIC (DURVA); supports PD-1/PD-L1 consolidation as standard of care for unresectable stage III NSCLC; PD-L1 not predictive of benefit |
| Theelen W, et al. (2021). Lancet Respir Med. | Metastatic NSCLC | Pooled analysis of two RCTs (PEMBRO-RT phase II; MDACC phase I/II); open-label | 148 (PEMBRO 76, PEMBRO+RT 72) | 75% chemotherapy; no prior ICI | PEMBRO 200 mg IV q3w | PEMBRO-RT: 24 Gy/3 fx (sequential) MDACC: 50 Gy/4 fx or 45 Gy/15 fx (concurrent) | Combination: RT + PEMBRO (sequential: within 1 week after the last RT; or concurrent); monotherapy: PEMBRO | Most irradiated sites: lung metastases (39%), intrathoracic LN (21%), lung primary (17%) | Combination vs monotherapy:  Best ARR 41.7% vs 19.7% (OR 2.96, p=0.0039);  mPFS 9.0 vs 4.4 mo (HR 0.67, p=0.045);  mOS 19.2 vs 8.7 mo (HR 0.67, p=0.0004) | No new safety concerns identified (pooled analysis) | In metastatic NSCLC, PEMBRO + RT significantly improves abscopal response rate, PFS, and OS without new safety signals; RT may convert "cold" tumors to "hot"; results warrant phase III validation |
| Jabbour SK, et al. (2021). JAMA Oncol. | Unresectable stage III NSCLC; ECOG 0–1; any PD-L1 | Phase II, nonrandomized (2-cohort), open-label | 214 (cohort A: 112 squamous/nonsquamous; cohort B: 102 nonsquamous) | Treatment-naïve; no prior ICI | PEMBRO 200 mg IV q3w × 17 cycles total | 60 Gy/30 fx; conventional fractionation | Cohort A: carboplatin + paclitaxel + PEMBRO × 1 cycle → concurrent RT + weekly carboplatin + paclitaxel × 6 weeks + PEMBRO × 2 cycles → PEMBRO × 14 cycles;  Cohort B: cisplatin + pemetrexed + PEMBRO × 3 cycles (RT in C2–3) → PEMBRO × 14 cycles | Primary tumor and involved lymph nodes | Cohort A vs B:  ORR 70.5% vs 70.6%;  DOR ≥12 mo 79.7% vs 75.6%;  1-year PFS/OS 67.1%/81.3% vs 71.6%/87.0%;  mPFS/OS not reached | Grade ≥3 pneumonitis:  A 8.0% (9/112) vs B 6.9% (7/102);  grade 3–5 TRAEs: A 64.3% (72/112) vs B 50.0% (51/102);  fatal TRAEs: A 4 (3.6%; pneumonitis), B 1 (1.0%; ILD) | Concurrent PEMBRO + cCRT achieved ~70% ORR in stage III NSCLC (median PFS/OS not reached), with responses observed regardless of histology and PD-L1 expression; grade ≥3 pneumonitis <10%, but grade 3–5 TRAEs were higher (50–64%); phase 3 validation is ongoing. |
| Peters S, et al. (2021). J Thorac Oncol. | Unresectable stage III NSCLC; ECOG 0–1 | Phase II, single-arm | 79 (77 treated) | Treatment-naïve; no prior chemo/RT/immunotherapy | NIV0 360 mg IV q3w × 4 cycles → 480 mg IV q4w consolidation × up to 1 year | 66 Gy/33 fx; conventional fractionation | cCRT (platinum-based chemo + RT) + NIV0 × 4 cycles (2nd and 3rd doses concurrent with cCRT) → NIV0 consolidation × up to 1 year | Primary tumor and involved lymph nodes | ORR 73.4%;  mPFS 12.7 mo;  1-year PFS 53.7% (prespecified target 60%, not met);  mOS 38.8 mo;  2-year OS 63.7%;  stage IIIA vs IIIB 2-year OS 81% vs 56% (P=0.037) | Grade ≥3 pneumonitis 11.4% (9/79); grade 3–4 TRAEs 11.6%; fatal TRAEs 7 (NIV0-related); 89% discontinuations during consolidation | Concurrent NIV0 + cCRT was feasible in stage III NSCLC; 1-year PFS 53.7% (prespecified 60% threshold not met), but median OS 38.8 months and 2-year OS 63.7% were numerically superior to historical controls; grade ≥3 pneumonitis 11.4%, 89% discontinuations during consolidation, highlighting tolerability challenges; phase 3 trials are warranted. |
| Bestvina CM, et al. (2022). J Thorac Oncol. 2022 | Stage IV NSCLC; ECOG 0–1; any PD-L1; brain metastases allowed | Randomized, open-label, phase I (timing exploration) | 37 (concurrent 18, sequential 19) | Treatment-naïve (no prior chemo/immunotherapy for metastatic disease); prior adjuvant/neoadjuvant chemotherapy allowed | NIV0 3 mg/kg IV q2w + IPI 1 mg/kg IV q6w × up to 2 years | Peripheral lung/liver/abdominal–pelvic: 45 Gy/3 fx; central lung/mediastinal: 50 Gy/5 fx; osseous/spinal: 30 Gy/3 fx | Concurrent: ICI initiated → SBRT within 2 weeks; Sequential: SBRT completed → ICI within 1–7 days | 1–5 (median 3–4); up to 4 isocenters | ORR 45.9%;  irradiated/nonirradiated lesion ORR 70.2%/33.3%;  DCR 62.2%;  mPFS 5.8 months;  mOS not reached (17-month follow-up) | Concurrent: no DLT; Sequential: central lung DLT rate 50% (2/4) → dose reduction; 1 grade 5 hemoptysis; overall grade ≥3 AEs 29.7% | Concurrent SBRT + dual ICI did not increase toxicity; both regimens were safe and effective; dual ICI + SBRT is feasible in widely metastatic NSCLC; supports further study of the concurrent approach. |
| Spigel DR, et al. (2022). J Clin Oncol. | Unresectable stage III NSCLC; no progression after cCRT; WHO PS 0–1; any PD-L1 | International, multicenter, randomized, double-blind,PBO-controlled phase III trial | 713 (709 treated: DURVA 473, PBO 236) | 100% received cCRT (≥2 cycles platinum-based; 60–66 Gy/30–33 fx); no prior ICI | DURVA 10 mg/kg IV q2w × up to 12 months | 60–66 Gy/30–33 fx | cCRT → DURVA consolidation (initiated 1–42 days after cCRT completion) | N/A | DURVA vs PBO  mPFS 16.9 vs 5.6 mo (HR 0.55);  5-y PFS 33.1% vs 19.0%;  mOS 47.5 vs 29.1 mo (HR 0.72);  5-y OS 42.9% vs 33.4%;  ORR 29.8% vs 18.3% | DURVA vs PBO  All-causality G3/4: 30.5% vs 26.1%;  AE-related discontinuation: 15.4% vs 9.8%;  most common: pneumonitis/radiation pneumonitis;  no new safety signals | Landmark phase III trial (5-year follow-up): Consolidation immunotherapy has become the global standard of care for this population. Its fundamental contribution is the first demonstration that this strategy provides durable long-term survival benefit, with significantly improved 5-year survival rates, suggesting that a subset of patients may achieve "clinical cure." |
| Zhou Q, et al. (2022). Lancet Oncol. | Locally advanced unresectable stage III NSCLC; no progression after cCRT or sCRT; ECOG 0–1; excluding EGFR/ALK/ROS1+ | Phase III, randomized, double-blind,PBO-controlled | 381 (SUGE 255, PBO 126) | 100% received cCRT or sCRT (≥2 cycles platinum-based); no prior ICI | SUGE 1200 mg IV q3w × up to 24 months (consolidation) | cCRT or sCRT: total dose 54–66 Gy (≥60 Gy in 83–84% of patients) | cCRT/sCRT → SUGE consolidation (initiated 1–42 days after CRT completion) | N/A | SUGE vs PBO  mPFS: 9.0 vs 5.8 mo (HR 0.64, p=0.0026);  12-mo PFS rate: 45.4% vs 25.6%;  OS: immature (HR 0.44, 95% CI 0.27–0.73) | SUGE vs PBO  Incidence 9% vs 6%;  Key toxicity ≥G3 pneumonitis 3% vs <1%;  Discontinuation due to AEs 11% vs 5%;  Treatment-related deaths 4 (2%) in SUGE group | Validation study in a Chinese population: SUGE consolidation significantly prolongs PFS (HR 0.64) with manageable safety; complements PACIFIC by demonstrating efficacy in both cCRT and sCRT (for patients unfit for or without access to cCRT), further expanding the population benefitting from consolidation immunotherapy; OS data immature, longer follow-up needed |
| Garassino MC, et al. (2022). J Thorac Oncol. | Unresectable stage III NSCLC; no progression after sCRT; ECOG 0–2; any PD-L1 | Phase II, open-label, single-arm | 117 | Sequential CRT (≤1 cycle overlap allowed); no prior ICI | DURVA 1500 mg IV q4w × up to 24 months | 60 Gy ± 10% (54–66 Gy); conventional fractionation | sCRT → DURVA consolidation (median 26 days after RT completion) | N/A | Median PFS 10.9 mo (95% CI: 7.3–15.6);  12-mo PFS rate 49.6%;  12-mo OS rate 84.1%;  24-mo OS rate 69.8%;  ORR 17.1% (95% CI 11.1–25.8) | Grade 3/4 PRAEs within 6 months 4.3% (5/117); AE-related discontinuation 21.4% | This extension study in a frailer population extends the established standard of care (PACIFIC regimen) to the clinically more common but understudied ‘sequential CRT’ frail population, demonstrating its feasibility and potential in this setting. The phase III PACIFIC-5 trial is ongoing. |
| Ross HJ, et al. (2024). JAMA Oncol. | Treatment-naïve, unresectable stage III NSCLC | Single-arm, phase II | 62 | Treatment-naïve; no prior chemotherapy, RT, or ICI | Induction: ATEZO 1200 mg IV q3w ×4 cycles → CRT + consolidation chemotherapy (ATEZO paused) → Consolidation/Maintenance: ATEZO 1200 mg IV q3w, from first ATEZO dose to complete 12 mo total | 60 Gy/30 fx | Induction ATEZO 4 cycles → RT + weekly carboplatin/paclitaxel (concurrent, ATEZO paused) → Consolidation chemotherapy 2 cycles (ATEZO paused) → ATEZO maintenance until 1 year total (from first dose) | Primary tumor and involved lymph nodes | 12-week DCR 74.2% (80% CI 65.7–81.4);  mPFS 30.0 mo (95% CI 15.8–NE);  24-mo OS rate 73.7% (95% CI 63.4–85.7);  mOS NR;  ORR 66.2% | ≥G3 TRAEs 30 (48.4%); ≥G3 irAEs 17 (27.4%); Grade 5 pneumonitis (n=1), Grade 4 Guillain-Barré syndrome (n=1) | The "induction-consolidation" immunotherapy strategy shows promising long-term PFS and OS in unresectable stage III NSCLC; notable toxicity (48.4% ≥G3 TRAEs) warrants careful management, but results provide a key rationale for phase III evaluation of this intensified regimen |
| Altorki NK, et al. (2021). Lancet Oncol. | Resectable early-stage NSCLC (clinical stages I–IIIA); ECOG 0–1 | Randomized, controlled, phase II, open-label | 60 (30 per group) | Treatment-naïve | DURVA 1120 mg IV infusion over 60 min q3w ×2 cycles | SBRT: 8 Gy ×3 fractions, 3 consecutive daily fractions | Combination: SBRT (before first DURVA) → DURVA ×2 cycles → surgery if no systemic progression; Monotherapy: DURVA ×2 cycles → surgery if no systemic progression | Primary tumor | MPR rate 53.3% (combination) vs 6.7% (monotherapy) (p<0.0001);  pCR rate (among MPR responders in combination group) 50% (8/16);  Surgical resection rate 87% (26/30) in both groups | Any-cause G3–4 AEs combination 20% (6/30) vs monotherapy 17% (5/30); No treatment-related deaths | Neoadjuvant DURVA + SBRT is well tolerated and safe, with significantly higher MPR rate (53.3% vs 6.7%) than DURVA alone; this neoadjuvant strategy warrants validation in a larger phase III trial |
| **Melanoma** | | | | | | | | | | | |
| Grimaldi AM, et al. (2014). Oncoimmunology. | Advanced melanoma (81% M1c) | Retrospective analysis | 21 | 95% received ≥1 prior systemic therapy (including chemotherapy); progressed after IPI therapy | IPI 3 mg/kg IV q3w ×4 cycles (95% completed 4 cycles; 1 patient received retreatment of 4 cycles) | WBRT: 30 Gy/10 fx (multiple brain metastases); SRT: 20 Gy/1 fx or 24 Gy/1 fx (1–3 brain metastases); Extracranial RT: 20 Gy/5 fx, 30 Gy/10 fx (bone/lymph nodes), 30 Gy/10 fx or 50 Gy/25 fx (skin/soft tissue) | Progression on IPI → sequential RT (median interval 5 months) | RT sites: brain 13 (WBRT 9, SRT 4); bone 4; lymph nodes 2; skin 2 | ARR 52% (11/21);  Local response 13 (62%);  Local→abscopal association 11/13 (85%);  mOS 22.4 mo (with AR) vs 8.3 mo (without AR);  mPFS (post-IPI) 4 mo | Not reported | Sequential RT after progression on IPI can induce abscopal responses and significantly prolong survival |
| Twyman-Saint Victor C, et al. (2015). Nature. | Stage IV melanoma with multiple metastases | Phase I clinical trial | 22 | Heavily pretreated (0–≥3 prior lines); 1 patient had prior immunotherapy (type unspecified) | IPI 3 mg/kg IV q3w × 4 cycles | Stratum 1 (lung/bone): 8 Gy × 2 or 8 Gy × 3; Stratum 2 (liver/subcutaneous): 6 Gy × 2 or 6 Gy × 3 (SBRT) | Single lesion irradiated, followed 3–5 days later by IPI q3w × 4 doses | 1 | Unirradiated lesions PR 18% (4/22), SD 18%, PD 64%;  mPFS 3.8 months;  mOS 10.7 months | Comparable to IPI monotherapy | RT + IPI was safe and feasible; abscopal effects in 18%; PD-L1 upregulation as dominant resistance mechanism, supporting triple combination (RT + anti-CTLA-4 + anti-PD-L1/PD-1). |
| Chandra RA, et al. (2015). Oncoimmunology. | Metastatic melanoma; 30% BRAF-mutant | Retrospective cohort study | 47 | Prior therapy included chemotherapy, targeted therapy, surgery, etc. (heterogeneous); no details on prior ICI exposure reported | IPI: 3 mg/kg IV q3w × 4 cycles (43 pts), with 10 pts receiving q12w maintenance; 4 pts received 10 mg/kg investigational regimen; median 4 cycles (range 1–18) | Overall: median 30 Gy (range 8–66)/median 3 Gy/fx (range 2–25); SRS/SRT: 20 Gy (range 18–25); WBRT: 30 Gy (range 30–37.5)/3 Gy/fx (range 2.5–3); Bone/soft tissue palliative RT: 30 Gy/3–4 Gy/fx | Conventional/hypofractionated RT + ipilimumab (variable timing) | SRS for ≤4 brain metastases; WBRT for multiple brain metastases | Abscopal effect evidence: Index lesion shrinkage increased from 11% pre-RT to 25% post-RT (p=0.03);  mOS 28 months;  5-year OS 20% | Not reported | Ipilimumab combined with RT can induce abscopal effects in metastatic melanoma, but this remains a rare clinical event; fractionated RT (particularly with fraction size ≤3 Gy) is more likely to induce out-of-field responses than single-fraction ablative RT; prospective studies are needed to validate optimal RT parameters |
| Sundahl N, et al. (2018). Int J Radiat Oncol Biol Phys. | Metastatic melanoma (stage IV) | Phase I, single-arm, dose-escalation | 13 (12 irradiated) | 6 (46%) treatment-naïve; 1 (8%) had prior NIV0; 5 (38%) had prior RT | IPI 3 mg/kg IV q3w × 4 cycles | 8 Gy × 3 (n=5), 10 Gy × 3 (n=1), 12 Gy × 3 (n=6) | 2 cycles IPI → SBRT (before 3rd cycle) → 2 cycles IPI | 1 | Local control rate 92% (11/12);  nonirradiated lesion clinical benefit 23% (3/13);  mOS 74 weeks | Grade 3/4 IPI-related AEs 25% (3/12; grade 4 colitis n=2, grade 3 diarrhea n=1); no RT-related DLT; MTD not reached | IPI + SBRT (up to 36 Gy/3 fx) was safe and feasible; toxicity profile comparable to IPI monotherapy; local control rate 92%, nonirradiated lesion clinical benefit 23%; MTD not reached, supporting further investigation |
| Roger A, et al. (2018). Oncoimmunology. | Advanced melanoma (AJCC IIIC–IV); 64% M1c; 60% LDH↑; 64% ECOG>0; 24% brain metastases | Prospective cohort (retrospective analysis of prospective data); single-center | 25 | 64% second-line or beyond; 28% BRAF-mutant (all received BRAFi+MEKi); 20% prior IPI; 32% prior chemotherapy | NIVO 3 mg/kg IV q2w (20 pts); PEMBRO 2 mg/kg IV q3w (5 pts); continued until PD or unacceptable toxicity | Hypofractionated RT (17 pts, median 26 Gy/3–5 fx); brain SRS (5 pts); conventional 30 Gy/10 fx (2 pts) | Emergency group (n=15): RT started at median 24 days (range -11 to 59) after anti-PD-1 initiation; Late RT group (n=10): RT started at median 5.4 months (range 3.8–11.2) after anti-PD-1 initiation (continued anti-PD-1 beyond PD) | Limited to single organ (24 pts); brain SRS for 1–5 lesions | Median follow-up 16.9 mo;  ORR (irradiated/non-irradiated) 36%/36%;  response correlation between fields (r=0.89, p<0.0001);  mPFS emergency 3.0 mo, late 16.2 mo;  mMSS emergency 9.9 mo, late 18.9 mo;  5 CR pts remained disease-free for median 9.5 mo after anti-PD-1 discontinuation | Incidence: 4% (1 grade 3 diarrhea) | A clinical study exploring combination timing: This study provides evidence that radiotherapy combined with anti-PD-1 therapy can induce abscopal effects. Key finding: clinical benefit was observed when RT was combined either early (rapid progression) or late (after PD) during anti-PD-1 treatment, with the late-combination group showing significantly superior PFS compared to the early-combination group. |
| Anvari A, et al. (2023). Hematol Oncol Stem Cell Ther. | Melanoma brain metastases (MBM) | Systematic review and meta-analysis (44 studies included) | 2498 (44 studies) | Prior treatment varied (chemotherapy/targeted therapy/ICI/WBRT, inconsistent across studies) | IPI (33 studies); PEMBRO (15 studies); NIVO (14 studies); IPI+NIVO combination (8 studies) | SRS (commonly 20 Gy/single fraction); WBRT (commonly 30 Gy/10 fractions) | ICI + RT; concurrent or sequential; <3 months interval defined as concurrent | Median 2–3 (range 1–30); SRS mostly for ≤5 lesions; WBRT for multiple lesions | Pooled ORR 38.6% (95% CI: 21.6–55.6);  Pooled OS benefit: combination vs ICI alone (HR 0.69); combination vs RT alone (HR 0.60);  mOS 14.44 months | Safety comparison: Combination therapy did not significantly increase the risk of ≥Grade 3 neurologic adverse events or radiation necrosis | This meta-analysis demonstrates that ICI combined with radiotherapy significantly improves overall survival in patients with melanoma brain metastases, without increasing high-grade CNS toxicity, providing high-level evidence for this challenging patient population. |
| **Small Cell Lung Cancer** | | | | | | | | | | | |
| Welsh JW, et al. (2020). J Thorac Oncol. | Limited-stage SCLC (36/40) and other neuroendocrine tumors (4/40); ECOG 0–2 | Phase I/II, single-arm, open-label | 40 | 32.5% received induction chemotherapy; 61% received PCI; ICI-naïve | PEMBRO 200 mg IV q3w (phase I: 100–200 mg) | 45 Gy/30 fx (1.5 Gy/fx, BID) | cCRT (etoposide + cisplatin/carboplatin × 4 cycles + RT) + PEMBRO (started concurrently with CRT, continued as consolidation/maintenance after CRT completion, up to 16 cycles) → PCI (optional) | Primary tumor and involved lymph nodes | ORR (irRC) 79%;  mPFS 19.7 mo;  mOS 39.5 mo;  2-year OS 65.8% | 1 DLT (grade 3 pericarditis); no grade 5; grade 4: 3; grade 3 pneumonitis: 3; esophagitis 42.5% (grade 1–2) | Concurrent PEMBRO + CRT was well tolerated in LS-SCLC (no grade 5 toxicity; grade 3 pneumonitis 8%), with encouraging efficacy (median OS 39.5 mo, 2-year OS 65.8%); provides a safety foundation for ongoing phase III trials. |
| Cheng Y, et al. (2024). N Engl J Med. | Limited-stage SCLC (stages I–III); no progression after cCRT | Phase III, randomized, double-blind,PBO-controlled | 730 (D 264, D+T 200, PBO 266) | 100% received cCRT (≥4 cycles platinum-based + etoposide); no prior ICI | D group: DURVA 1500 mg IV q4w × up to 24 months;  D+T group: DURVA 1500 mg + Tremelimumab 75 mg IV q4w ×4 cycles, then DURVA monotherapy maintenance up to 24 months | 45 Gy/30 fx (BID) or 60–66 Gy/30–33 fx (QD); PCI allowed but not mandatory | cCRT → consolidation immunotherapy (initiated 1–42 days after cCRT completion) | **N/A** | D vs PBO  mOS 55.9 vs 33.4 mo (HR 0.73, 98.321% CI 0.54–0.98, p=0.01);  mPFS 16.6 vs 9.2 mo (HR 0.76, 97.195% CI 0.59–0.98, p=0.02);  D+T group results remain blinded | D vs PBO  G3/4 AEs 24.4% vs 24.2%; Discontinuation due to AEs 16.4% vs 10.6%; ≥G3 pneumonitis/radiation pneumonitis 3.1% vs 2.6%; Fatal AEs 2.7% vs 1.9% | First phase III trial to demonstrate significant OS and PFS benefit with consolidation immunotherapy in LS-SCLC; D regimen is safe and tolerable; has the potential to become the new standard of care. |
| Perez BA, et al. (2021). Int J Radiat Oncol Biol Phys. | Extensive-stage SCLC; stable disease or response after platinum-based chemotherapy | Phase I/II, single-arm | 21 | Platinum-based chemotherapy completed (4–6 cycles); no prior ICI; 28.6% had brain metastases (19% received WBRT) | IPI 3 mg/kg + NIV0 1 mg/kg IV q3w × 4 cycles → NIV0 480 mg IV q4w maintenance × up to 1 year | 30 Gy/10 fx | Platinum-based chemotherapy → TRT → 2 weeks later → IPI + NIVO × 4 cycles → NIVO maintenance | Initially involved primary tumor and regional lymph nodes | 6-month PFS rate 24% (prespecified 44% target not met); mPFS 4.5 mo; mOS 11.7 mo; 12-month OS rate 48% | Grade ≥3 irAEs 52.4% (11/21); grade 3 pneumonitis 19%; grade 3 diarrhea 19%; no treatment-related deaths; 24% discontinued due to AEs | TRT followed by sequential IPI+NIVO did not improve 6-month PFS (comparable to historical control), but 12-month OS (48%) was numerically superior to historical expectations; high-grade irAEs were common; baseline CD8⁺ T-cell infiltration and peripheral CD8⁺ T-cell reduction may predict benefit |
| **Lymphoma** | | | | | | | | | | | |
| Frank MJ, et al. (2018). Cancer Discov. | Low-grade B-cell lymphoma (predominantly follicular) | Phase I/II, multicenter, open-label, dose-escalation trial | 29 | Treatment-naïve | Intratumoral SD-101 (TLR9 agonist): 1, 2, 4, or 8 mg weekly × 5 injections | 4 Gy (2 Gy × 2 days) | Low-dose RT (4 Gy/2 fx) to Lesion A, followed by intratumoral SD-101 injection on the same day, weekly × 5 | 1 (Lesion A); distant lesions (Lesion B) assessed for abscopal effect | ORR 27.6% (8/29; CR 1, PR 7);  distant lesion volume reduction 82.8% (24/29) | Incidence: 27.6% (8/29); predominantly flu-like symptoms and neutropenia; no grade 4 or serious adverse events | Low-dose RT + intratumoral TLR9 agonist in situ vaccination is safe and induces systemic responses in untreated indolent lymphoma; low baseline Tregs, proliferating CD8⁺ T, and Granzyme B⁺ CD8⁺ T cells predict better response |
| Witzig TE, et al. (2002). J Clin Oncol. | Relapsed/refractory low-grade B-cell NHL (predominantly follicular) | Phase III, randomized, controlled, multicenter; open-label | 143 (^90^Y group 73, Control group 70) | Median 2 prior lines (range 1–6); CHOP/CVP predominant; 49% resistant to last chemotherapy; no prior RIT/SCT; no prior ICI | ^90^Y group: RTX 250 mg/m² IV d1,d8 + 90Y-IT 0.4 mCi/kg IV d8 (max 32 mCi);  Control group: RTX 375 mg/m² IV qw ×4 weeks | RIT (⁹⁰Y-IT): β-emitter (2.3 MeV), half-life 64h, soft-tissue range 5mm; estimated normal organ ≤20 Gy, bone marrow ≤3 Gy | ^90^Y group: RTX (d1,d8) + ¹¹¹In-IT 5 mCi (d1, imaging/dosimetry) + 90Y-IT (d8);  Control group: RTX 375 mg/m² qw ×4 | **N/A** | ^90^Y group vs Control group: ORR: 80% vs 56% (P=0.002); CR rate: 30% vs 16% (P=0.04); Durable response ≥6 months: 64% vs 47% (P=0.03); mTTP: 11.2 vs 10.1 months (P=0.173); mTTNT: NR vs 13.1 months (P=0.084) | Primary toxicity: reversible myelosuppression; Grade 3–4 neutropenia 57%, thrombocytopenia 60% | ^90^Y-IT significantly improves ORR and CR rate versus RTX in relapsed/refractory indolent/follicular NHL, with acceptable safety; TTP was not significantly prolonged, but durable response rate was significantly improved; RIT provides an effective chemo-free option for chemotherapy-refractory/elderly patients and may serve as induction prior to subsequent chemotherapy or transplant |
| Lucchini E, et al. (2021). Hematol Rep. | R/R cHL | Multicenter retrospective analysis | 12 | Heavily pretreated: 58% ≥4 prior lines; 50% ASCT; 92% BV; no prior ICI | NIV0 (9, 75%) or PEMBRO (3, 25%) at standard doses; median 15 administrations | Median 30 Gy (range 30–40 Gy); conventional fractionation (1.8–2.0 Gy/fx); median duration 22 days | Concurrent (n=4) or sequential (n=8) RT; within 8 weeks before/after ICI | Involved sites (mediastinal, cervical, inguinal, etc.) | ORR 100% (12/12);  CR 58.3% (7/12), PR 41.7% (5/12);  at median follow-up 18 months: 92% (11/12) achieved CR | 2 (17%) grade 3 interstitial pneumonia; no treatment interruption | ICI combined with RT appears feasible and effective as a bridge to transplant in heavily pretreated R/R cHL (ORR 100%, CR 58%; 18-month CR 92%); however, evidence is limited by the retrospective design and small sample size, warranting prospective validation. |
| Sun P, et al. (2022). Front Immunol. | Treatment-naïve, localized (stage I–II) NK/T-cell lymphoma | Retrospective analysis | 8 | Treatment-naïve; no prior ICI | Anti-PD-1 antibody (PEMBRO/NIV0/sintilimab) + anlotinib (12 mg D1–14) + pegaspargase (2500 U/m² D1) | IMRT: PTV-GTV 54–56 Gy/25–26 fx; PTV-CTV1 50–50.7 Gy/25–26 fx; PTV-CTV2 45 Gy/25–26 fx | 3 cycles of immuno-targeted-chemotherapy → IMRT → 3 additional cycles ("sandwich" regimen) | Primary tumor and involved lymph node regions | Best response ORR 100% (CR 87.5%, PR 12.5%);  3-year PFS/OS both 100% (median follow-up 35.5 months) | Only 1 (12.5%) grade 3 TRAE (hyperbilirubinemia); no grade 4/5 TRAEs | Demonstrates promising antitumor activity and favorable safety profile in localized NK/T-cell lymphoma; however, evidence is limited (retrospective, single-arm, n=8), warranting prospective validation in larger cohorts. |
| **Other Solid Tumors** | | | | | | | | | | | |
| Tang C, et al. (2017). Clin Cancer Res. | Metastatic solid tumors (multiple types); liver/lung lesions amenable to SABR | Phase I, dose-finding (5 parallel non-randomized groups) | 35 (31 evaluable for response) | Heavily pretreated: 89% (31/35) had prior systemic therapy (median 2 lines); 63% (22/35) prior RT; 2 had prior immunotherapy (1 CTLA-4 inhibitor) | IPI 3 mg/kg IV q3w × 4 doses | 50 Gy/4 fx (Groups 1–4); 60 Gy/10 fx (Group 5) | Concurrent (day after 1st IPI) or sequential (week after 2nd IPI) RT; | 1 | Out-of-field irPR 10% (3/31);  clinical benefit (out-of-field irSD ≥6 months or irPR/CR) 23% (7/31);  mPFS 3.2 months; mOS 10.2 months | Grade 3 TRAEs 34% (12/35); colitis 11% (4/35); DLT 6% (2/35; liver group); no grade 4/5 TRAEs | IPI + SABR (50 Gy/4 fx or 60 Gy/10 fx) was safe and feasible; out-of-field clinical benefit rate 23%; liver irradiation induced greater systemic T-cell activation than lung; increased CD8⁺ T cells and 4-1BB/PD1 expression on CD8⁺ T cells may predict clinical benefit. |
| Luke JJ, et al. (2018). J Clin Oncol. | Advanced/metastatic solid tumors (multiple types) | Phase I, single-arm | 73 (79 enrolled) | Heavily pretreated: median 5 prior lines (IQR 3–8); 5 (6.8%) had prior anti-PD-1 | PEMBRO 200 mg IV q3w, median 4 cycles | Peripheral lung/liver/abdominal–pelvic: 45 Gy/3 fx; central lung/mediastinal/cervical: 50 Gy/5 fx; osseous/spinal: 30 Gy/3 fx | Multisite SBRT → PEMBRO (sequential, initiated within 7 days after SBRT) | 2–4 (94.5% had 2) | ORR 13.2% (9/68);  nonirradiated lesion ORR 13.5% (7/52);  mPFS 3.1 months;  mOS 9.6 months | DLT rate 9.7% (6/62); grade 3: pneumonitis (n=3), colitis (n=2), hepatic toxicity (n=1); no RT dose reduction | Multisite SBRT followed by PEMBRO was well tolerated (DLT rate 9.7%); post-SBRT IFN-γ-associated gene expression correlated with nonirradiated tumor response (ρ = -0.78, p = 0.023), supporting further investigation of this combination. |
| Kelly RJ, et al. (2021). N Engl J Med. | Esophageal/gastroesophageal junction cancer; residual pathological disease after neoadjuvant CRT + R0 resection | Phase III, randomized, double-blind,PBO-controlled | 794 (NIVO 532, PBO 262) | 100% received neoadjuvant CRT (investigator choice of regimen, platinum-based + fluoropyrimidine or taxane); no prior ICI | NIVO 240 mg IV q2w ×16 wks → NIVO 480 mg IV q4w (total treatment up to 1 year) | Neoadjuvant CRT: common regimens 41.4–50.4 Gy/23–28 fx (investigator choice) | Neoadjuvant CRT + R0 resection (residual pathological disease) → adjuvant NIVO/PBO (initiated 4–16 weeks after surgery) | N/A | NIVO vs PBO  mDFS 22.4 vs 11.0 mo (HR 0.69, 96.4% CI 0.56–0.86, p<0.001);  Distant metastasis–free survival 28.3 vs 17.6 mo (HR 0.74);  Consistent benefit across all prespecified subgroups (including PD-L1, histology, nodal status) | NIVO vs PBO  G3/4 TRAEs 13% vs 6%;  Discontinuation due to AEs 9% vs 3%;  No treatment-related deaths | First phase III trial to demonstrate significant DFS benefit with adjuvant immunotherapy after neoadjuvant CRT in esophageal cancer; NIVO reduces recurrence/death risk by 31%; acceptable safety profile; supports NIVO as new standard of care for adjuvant therapy in this population |
| McBride S, et al. (2021). J Clin Oncol. | Metastatic HNSCC (including nasopharyngeal carcinoma); ECOG 0–2 | Phase II, randomized, open-label | 62 (NIVO 30, NIVO+SBRT 32) | 88.7% had prior chemotherapy; ICI-naïve | NIVO 3 mg/kg IV q2w × up to 96 weeks | SBRT: 9 Gy × 3 fx (every other day) | NIVO initiated → SBRT between 1st and 2nd NIVO dose → NIVO continued (sequential) | 1 | Combination vs monotherapy  nonirradiated lesion ORR 29.0% vs 34.5% (P=0.86);  mPFS 2.6 vs 1.9 mo (P=0.79);  mOS 13.9 vs 14.2 mo (P=0.75);  (no statistically significant differences) | Combination vs monotherapy: 9.7% vs 13.3% (P=0.70); adding SBRT did not significantly increase toxicity | SBRT plus NIVO did not improve ORR, PFS, or OS in unselected metastatic HNSCC, with no abscopal effect observed; the combination was safe; virus-negative patients may warrant further investigation. |
| Lee NY, et al. (2021). Lancet Oncol. | Locally advanced squamous cell carcinoma of the head and neck (LA-SCCHN); previously untreated; PD-L1 unselected; ECOG 0–1 | Phase III, randomized, double-blind,PBO-controlled, international multicenter | 697 (AVE 350, PBO 347) | Treatment-naïve; no prior ICI | AVE 10 mg/kg IV q2w | IMRT 70 Gy/35 fx (7 weeks) | AVE/PBO lead-in (single dose 7 days pre-RT) → cCRT (cisplatin q3w + IMRT) + AVE/PBO q2w (concurrent) → AVE/PBO maintenance q2w × up to 12 months | Primary tumor + regional lymph nodes | AVE vs PBO  mPFS NR vs NR (HR 1.21, 95% CI 0.93–1.57, favoring PBO, p=0.92);  median follow-up 14.6 vs 14.8 mo;  primary endpoint not met;  OS data not reported | AVE vs PBO  ≥G3 TRAEs 74% vs 71%; serious TRAEs 36% vs 32%;  treatment-related deaths 2 (1%) vs 1 (<1%) | First phase III trial to evaluate cCRT + immunotherapy in LA SCCHN, but did not meet the primary PFS endpoint (HR 1.21, favoring PBO); suggests that cCRT + immunotherapy did not show benefit in this population and was associated with increased toxicity, contrasting with positive trials such as PACIFIC; provides important negative reference for future exploration of timing/regimen selection for immunotherapy combined with chemoradiotherapy |
| Tsai CJ, et al. (2024). Lancet. | Oligoprogressive metastatic breast cancer (47) / NSCLC (59); ≤5 progressive lesions; ≥1 prior line of systemic therapy; 86% NSCLC without driver mutations; 1/3 breast cancer triple-negative | Randomized, open-label, phase II controlled trial | 106 (SBRT 55, SOC 51) | All received ≥1 line of systemic therapy; 80% of NSCLC had prior immunotherapy, 21% of breast cancer had prior immunotherapy (types not reported) | Per SOC (investigator's choice, not a study intervention) | Most common: 9–10 Gy ×3 fx or 10 Gy ×5 fx | SBRT (all oligoprogressive lesions) + continued SOC vs SOC alone | Median 2 (range 1–5) | SBRT vs SOC:  mPFS (overall): 7.2 vs 3.2 mo (HR 0.53, p=0.003);  Subgroup: NSCLC 10.0 vs 2.2 mo (HR 0.41, p=0.004); breast cancer 4.4 vs 4.2 mo (HR 0.78, p=0.20);  OS: no significant difference in either group (including subgroups);  ctDNA decreased only in NSCLC | SBRT group: radiation pneumonitis (n=1), pain exacerbation (n=1); SOC group: not separately reported | First randomized evidence: SBRT + continued SOC prolongs PFS in oligoprogressive NSCLC (HR 0.41), with no benefit in breast cancer; 80% of NSCLC patients had prior immunotherapy history, providing reference value for radiotherapy-immunotherapy combination strategies |
| **Abbreviations:** AE, adverse event; AEs, adverse events; ARR, abscopal response rate; ASCT, autologous stem cell transplantation; ATEZO, atezolizumab; AVE, avelumab; BID, twice daily; BV, brentuximab vedotin; cCRT, concurrent chemoradiotherapy; cHL, classical Hodgkin lymphoma; CI, confidence interval; CR, complete response; CRT, chemoradiotherapy; CT, computed tomography; CTLA-4, cytotoxic T-lymphocyte-associated protein 4; DC, disease control; DCR, disease control rate; DFS, disease-free survival; DLT, dose-limiting toxicity; DOR, duration of response; DURVA, durvalumab; ECOG, Eastern Cooperative Oncology Group; EGFR, epidermal growth factor receptor; fx, fraction(s); GTV, gross tumor volume; Gy, gray; HIGRT, hypofractionated image-guided radiotherapy; HNSCC, head and neck squamous cell carcinoma; HR, hazard ratio; ICI, immune checkpoint inhibitor; IFNβ, interferon-beta; IMRT, intensity-modulated radiotherapy; IPI, ipilimumab; irAE, immune-related adverse event; irRC, immune-related response criteria; ITT, intention-to-treat; IV, intravenous; LAT, local ablative therapy; LDH, lactate dehydrogenase; LN, lymph node; LS-SCLC, limited-stage small cell lung cancer; MBM, melanoma brain metastases; mo, month(s); MPR, major pathological response; MSS, melanoma-specific survival; MTD, maximum tolerated dose; N, number; N/A, not applicable; NE, not estimable; NHL, non-Hodgkin lymphoma; NIVO, nivolumab; NR, not reached; NSCLC, non-small cell lung cancer; OR, odds ratio; ORR, overall response rate; OS, overall survival; PBO, placebo; PCI, prophylactic cranial irradiation; pCR, pathological complete response; PD, progressive disease; PD-1, programmed cell death protein 1; PD-L1, programmed cell death ligand 1; PEMBRO, pembrolizumab; PFS, progression-free survival; PR, partial response; PS, performance status; PTV, planning target volume; q2w, every 2 weeks; q3w, every 3 weeks; q4w, every 4 weeks; q6w, every 6 weeks; q12w, every 12 weeks; QD, once daily; R/R, relapsed/refractory; RBC, regional brain control; RECIST, Response Evaluation Criteria in Solid Tumors; RIT, radioimmunotherapy; RT, radiotherapy; RTX, rituximab; SBRT, stereotactic body radiotherapy; SCLC, small cell lung cancer; sCRT, sequential chemoradiotherapy; SCT, stem cell transplantation; SD, stable disease; SOC, standard of care; SRS, stereotactic radiosurgery; SRT, stereotactic radiotherapy; SUGE, sugemalimab; TCR, T cell receptor; TMDD, time to metastatic disease or death; TRAE, treatment-related adverse event; TRT, thoracic radiotherapy; TTP, time to progression; TTNT, time to next treatment; vs, versus; WBRT, whole-brain radiotherapy; WHO, World Health Organization; 90Y-IT, yttrium-90 ibritumomab tiuxetan; 111In-IT, indium-111 ibritumomab tiuxetan.  Notes: 1.Key survival data (e.g., OS) for some studies (e.g., Zhou Q, 2022; Ross HJ, 2024) were immature at the time of reporting (interim analyses). 2.Safety note: ≥Grade 3 TRAEs refer to grade ≥3 adverse events judged by investigators as related to study treatment. Definitions and attribution criteria for adverse event collection may vary across studies; caution is warranted when comparing across trials. 3.Combination timing definitions: Concurrent—overlapping administration of RT and immunotherapy; Sequential—immunotherapy initiated after completion of RT; Consolidation—treatment administered after definitive therapy (e.g., concurrent chemoradiotherapy) to prolong response. 4.Data source: Data in this table are extracted from original study publications to summarize key findings. For detailed information and updated results, please refer to the original articles. 5.Response assessment: Response assessments of irradiated and non-irradiated lesions were based on RECIST criteria and/or immune-related response criteria (irRC) as specified in each original study. Definitions of measurable lesions (e.g., index lesions, all target lesions) varied across studies; caution is warranted when comparing abscopal response rates across trials. 6.PD-L1 expression: PD-L1 expression was assessed using various assays (e.g., 22C3, SP263, 28-8, SP142) with different cutoff values across studies; direct comparisons should be interpreted with caution. PD-L1 status was not always required for enrollment in some studies. | | | | | | | | | | | |
